# Single-molecule quantitation of RNA-binding protein occupancy and stoichiometry defines a role for Yra1 (Aly/REF) in nuclear mRNP organization

**DOI:** 10.1016/j.celrep.2023.113415

**Published:** 2023-11-14

**Authors:** Ryuta Asada, Andrew Dominguez, Ben Montpetit

**Affiliations:** 1 Department of Viticulture and Enology, University of California, Davis, Davis, CA 95616, USA; 2 Biochemistry, Molecular, Cellular, and Developmental Biology Graduate Group, University of California, Davis, Davis, CA 95616, USA; 3 Lead contact

## Abstract

RNA-binding proteins (RBPs) interact with mRNA to form supramolecular complexes called messenger ribonucleoprotein (mRNP) particles. These dynamic assemblies direct and regulate individual steps of gene expression; however, their composition and functional importance remain largely unknown. Here, we develop a total internal reflection fluorescence-based single-molecule imaging assay to investigate stoichiometry and co-occupancy of 15 RBPs within mRNPs from *Saccharomyces cerevisiae*. We show compositional heterogeneity of single mRNPs and plasticity across different growth conditions, with major co-occupants of mRNPs containing the nuclear cap-binding complex identified as Yra1 (1–10 copies), Nab2 (1–6 copies), and Npl3 (1–6 copies). Multicopy Yra1-bound mRNPs are specifically co-occupied by the THO complex and assembled on mRNAs biased by transcript length and RNA secondary structure. Yra1 depletion results in decreased compaction of nuclear mRNPs demonstrating a packaging function. Together, we provide a quantitative framework for gene- and condition-dependent RBP occupancy and stoichiometry in individual nuclear mRNPs.

## In brief

Asada et al. characterize the stoichiometry and co-occupancy of RBPs related to mRNA biogenesis and export in single mRNPs, identifying gene- and condition-dependent plasticity. Of these RBPs, Yra1 demonstrates highly variable stoichiometry and is required for mRNP compaction, highlighting the role of varied RBP composition in mRNP organization.

## Graphical Abstract

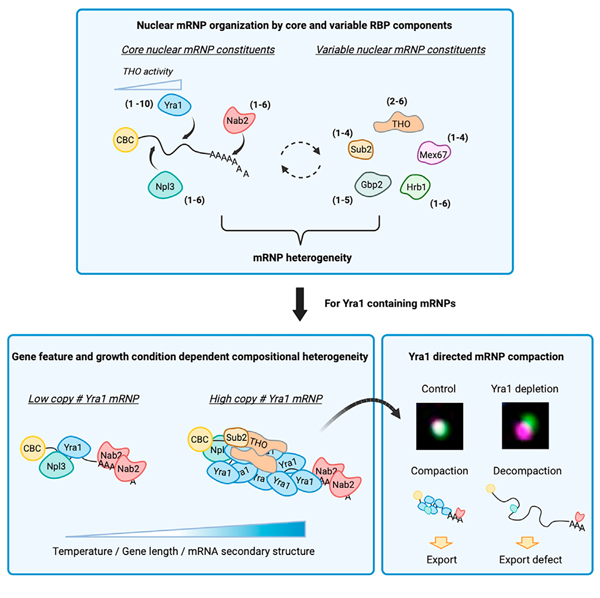

## INTRODUCTION

Regulated gene expression emerges from the summation of transcription, mRNA processing, export, localization, translation, and decay. These events are directed by dynamic interactions involving RNAs, RNA-binding proteins (RBPs), and associated factors in the form of messenger ribonucleoprotein (mRNP) particles.^1^ RBPs engage pre-mRNAs co-transcriptionally to facilitate transcript maturation (e.g., 5′ capping, splicing, cleavage, and polyadenylation).^2–4^ Progress along the gene expression pathway from nucleus to cytoplasm is further accompanied by the gain and loss of RBPs from mRNPs to direct steps within the gene expression pathway that includes export, translation, and decay.^3–5^ In *S. cerevisiae*, core RBPs engaged in mRNP biogenesis and export have been identified and well characterized (summarized in Figure S1), which are also well conserved in metazoans. Recent proteome-wide analyses of mRNA-bound RBPs have identified thousands of RBPs in both yeast and human cells,^6–17^ emphasizing the complexity and diversity of post-transcriptional gene regulation organized by RBP interaction networks.

Transcriptome-wide approaches have cataloged the mRNAs bound by RBPs including the binding position of RBPs along transcripts.^18–20^ These data have provided insights into the *in vivo* RNA-binding patterns of mRNP biogenesis and export factors (Figure S1); however, they represent collective interactions across mRNPs at all stages of gene expression. This limits the conclusions that can be made about individual mRNPs, since it is not possible to infer from these data if two RBPs mapping to the same transcripts were ever present in the same mRNP. This leaves questions about the functional role of mRNP heterogeneity across transcripts generated from different genes, or the same gene in the context of regulated gene expression, unaddressed. As such, it remains a major challenge to understand how mRNP architecture (i.e., the RBPs present in an mRNP, their stoichiometry, and overall topological organization) facilitates regulated gene expression at the level of the functional unit, which is an individual RNA-protein complex.

**Figure 1.**
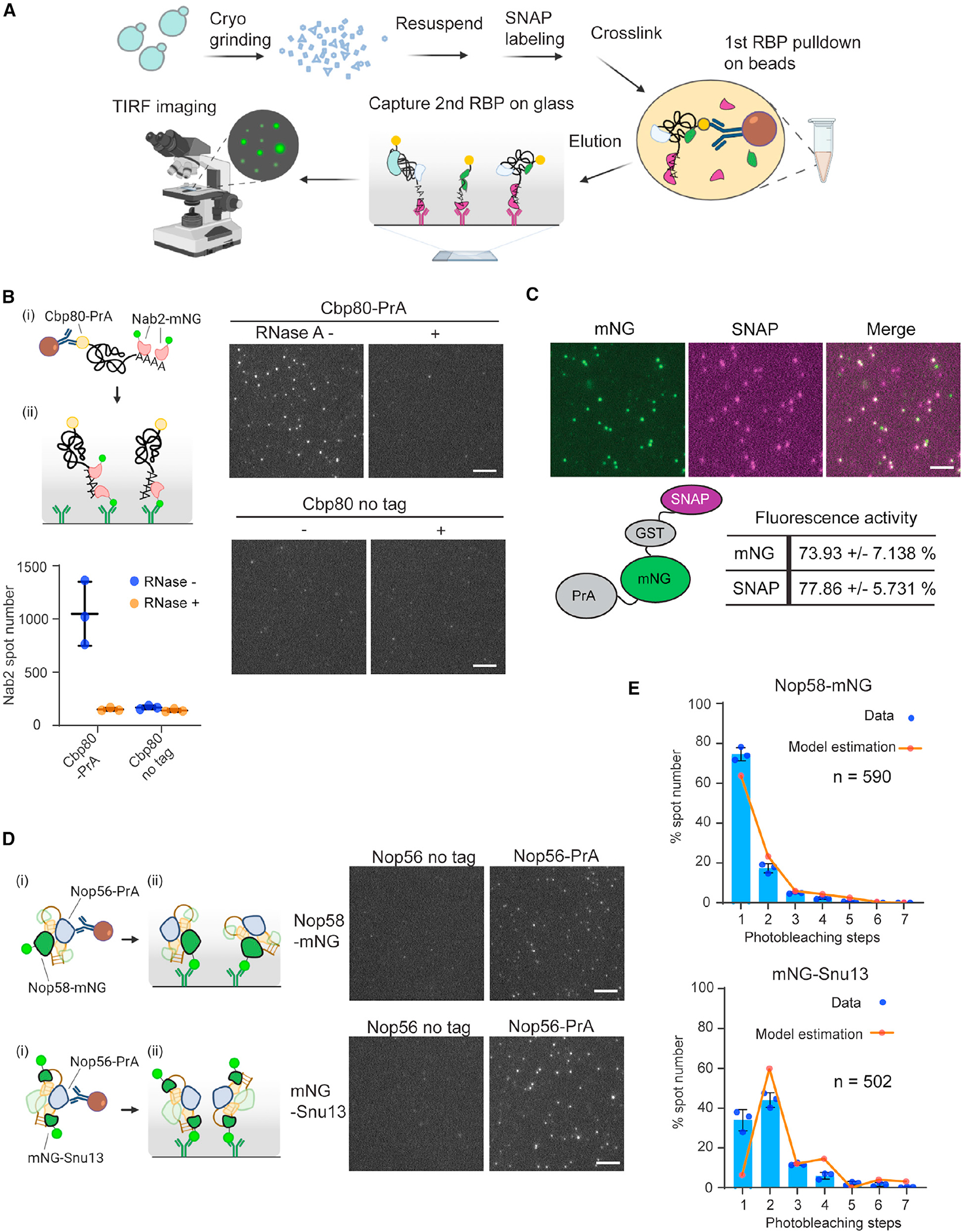
mRNP-SiMPull for characterization of *in vivo* mRNP composition (A) Schematic representation of mRNP-SiMPull procedure for isolating mRNPs from yeast cells and single-molecule imaging of RBP components. (B) Cartoon schematic of (i) Nab2-mNG imaging in mRNP-SiMPull with IgG-beads targeting nuclear cap-binding complex component Cbp80-PrA followed by (ii) mRNP capture via mNG antibody on the glass surface. Representative TIRF images of Nab2-mNG obtained by mRNP-SiMPull from cell lysates expressing Cbp80-PrA or untagged Cbp80 (no tag) with or without RNase A treatment. Graph shows the number of detected spots in triplicate experiments with mean and standard deviation (error bar). (C) A PrA-mNG-GST-SNAPf-3HA fusion protein was used to determine fluorescent reporter activity in the mRNP-SiMPull procedure. Images show PrA-mNG-GST-SNAPf-3HA captured on the TIRF slide via hemagglutinin (HA) antibody after labeling with SNAP-surface 549. Co-localization of mNG and SNAPf tag spots was calculated with the mean and standard deviations shown from triplicate experiments. (D) Nop58-mNG and mNG-Snu13 imaging with Nop56-PrA pull-down in mRNP-SiMPull. Pull-down was performed by (i) IgG-beads targeting Nop56-PrA followed by (ii) mNG antibody capture of snoRNP complexes on the glass surface. Representative TIRF images of Nop58-mNG and mNG-Snu13 analyzed in a Nop56-PrA pull-down and by mRNP-SiMPull. (E) Stoichiometry distribution of Nop58 and Snu13 in Nop56 pull-down analyzed by photobleaching steps analysis. Blue bars show mean data with standard deviation with dots showing individual data points in triplicate experiments. Orange line displays the expected complex stoichiometry distribution following correction for fluorescent reporter activity using finite mixture modeling. Image scale bars, 5 μm.

One aspect of mRNP architecture to consider is the spatial organization of the mRNA itself. It is known that the positioning of transcript regions in proximity to each other promotes pre-mRNA splicing and translation regulation.^21,22^ For example, recent single-molecule fluorescence *in situ* hybridization (smFISH) studies have documented changes in mRNP compaction in relation to transcription, export, and translation status.^23–25^ Proper packaging and compaction of the mRNA is likely to have benefits that include preventing the mRNA from being targeted for decay, promoting efficient export through a nuclear pore complex (NPC), and preventing intra- or intermolecular interactions that interfere with gene expression.^26,27^ Electron microscopy studies have shown that the ~35-kb Balbiani ring mRNA in *C. tentans* is compacted ~200-fold into a nuclear mRNP with a diameter of ~50 nm that changes shape during nuclear export.^28,29^ *S. cerevisiae* mRNPs are similarly compacted into a heterogeneous set of particles with dimensions that correlate with transcript length.^30,31^ In humans, data suggest that the exon junction complex (EJC) and serine and arginine-rich (SR) proteins cooperate to promote mRNA packaging^32,33^; however, a lack of EJC components in *S. cerevisiae* indicates that other RBPs must fulfill this function.^34^ While a molecular understanding of mRNP architecture is lacking, it is expected that one or more RBPs form a structural scaffold that organizes the mRNA.^27^ The identification of RBPs associated with mRNA biogenesis (describedin FigureS1) provides candidates for mRNP packaging.^5,26^ Notably, two recent studies have defined protein-protein and protein-RNA interaction networks in human cells and *S. cerevisiae* that would promote mRNA packaging, with both studies identifying Yra1 (Aly/REF) and the TREX complex as key packaging components.^30,35^ Still, the composition, organization, and heterogeneity among individual nuclear mRNPs remain largely unknown.

Here, a total internal reflection fluorescence (TIRF)-based imaging method termed mRNP single-molecule pull-down (mRNP-SiMPull) was developed to assess RBP stoichiometry and co-occupancy within individual mRNPs isolated from *Saccharomyces cerevisiae*. Using this approach, the stoichiometries of 15 RBPs within cap-binding complex (CBC) containing mRNPs were measured, the co-occupancy of select RBPs was determined, and plasticity in RBP composition was demonstrated in response to altered growth conditions. Our quantitative measures of individual mRNP compositions demonstrate that nuclear mRNPs are highly heterogeneous with a common set of RBP constituents (i.e., CBC, Npl3, Yra1, and Nab2) with varied occupancy and stoichiometry with other RBPs. Of these, Yra1 is a highly variable copy number component displaying gene feature-dependent occupancy that is required for the formation of export-competent compacted nuclear mRNPs.

## RESULTS AND DISCUSSION

### Establishment of the mRNP single-molecule pull-down (mRNP-SiMPull) methodology

A TIRF-based imaging method, mRNP-SiMPull, was established for the quantitative analysis of single mRNPs (Figure 1A), which was motivated by published methods aimed at the quantitation of protein complexes (SiMPull) and reconstituted splicing reactions (CoSMoS).^36,37^ Briefly, yeast cells are broken by cryogrinding, and complexes are isolated from a minimally cross-linked formaldehyde-treated lysate via pull-down of the nuclear CBC (composed of a Cbp80 and Cbp20 heterodimer^38^) using protein A (PrA)-tagged Cbp80 (Cbp80-PrA). Subsequently, mRNPs are eluted from the beads by proteolytic cleavage and loaded onto a glass slide where individuals mRNPs are captured by an antibody directed against a second RBP of interest tagged with mNeonGreen (mNG) or fluorescently labeled SNAP tag (SNAPf). Captured material is imaged using TIRF microscopy. Since fluorescent molecules photobleach stochastically, the counting of photobleaching steps provides RBP stoichiometry information in each detected spot (Figure S2A). Co-localization analysis can be performed using strains that express RBPs differentially tagged with mNG and SNAPf to provide RBP co-occupancy information within individual mRNP complexes. Importantly, each time mRNP-SiMPull is performed, data from a control strain lacking a PrA tagged subunit (Cbp80 or other target RBP) is collected. These data are used to perform a background subtraction, accounting for non-specific binding of fluorescent RBPs and daily variability in extract and slide preparation (see STAR Methods).

To begin to assess RBPs with mRNP-SiMPull, the presence of the nuclear poly(A)-RNA-binding protein (PABP) Nab2^39^ was assayed within Cbp80-containing mRNPs (i.e., isolation of capped and polyadenylated mRNPs). Using a Cbp80-PrA/Nab2-mNG strain, complexes of varied brightness were captured and detected, which were absent from a control strain lacking the PrA tag (Figure 1B). The Nab2-mNG spots were reduced to background levels upon addition of RNase A, indicating that detected signals represent mRNPs (Figure 1B). To confirm physiological relevance, mixing experiments were performed using lysates that individually contained Cbp80-PrA or Nab2-mNG. Upon mixing, detected Nab2-mNG spot numbers were comparable to the untagged control (Figure S2B), which was also the case for another RBP, the export adaptor Yra1 (Figure S2C). These data indicate that spots detected in Cbp80-PrA purifications represent mRNPs generated in the living cell, not RBP-mRNA interactions generated *in vitro* or non-specific interactions captured by crosslinking.

RBP dissociation from isolated mRNPs during the mRNP-SiMPull procedure is a possibility; hence Nab2 stoichiometry was assessed in a normally processed sample (~50 min) and after an extended incubation after immunoprecipitation (IP) (~100 min total). The extended incubation did not alter the Nab2-mNG stoichiometry distribution pattern (Figure S2D), indicating that mRNP architecture information was maintained. It was also observed that Nab2 stoichiometry values were not impacted by the tag used (e.g., mNG vs. SNAPf, Figure S2E), and measured fluorescent intensities of isolated mRNPs correlated with the number of observed photobleaching steps (Figure S2F). These data support the validity of the step-counting data.

To further ensure the accuracy of collected data, which may be impacted by inactive fluorescent reporters, the activities of mNG and SNAPf were assessed using a yeast expressing a PrA-mNG-GST-SNAPf-3xHA fusion protein (Figure 1C). Following the mRNP-SiMPull procedure, detected SNAP labeled spots were 74% ± 7% positive for mNG, suggesting~25% of mNG molecules were inactive, which closely matches what has been reported for GFP.^40^ Similarly, detected mNG spots were 78% ± 6% positive for SNAPf. With these estimates, RBP stoichiometry measurements can be corrected to compensate for the probability of mRNPs containing a non-fluorescent molecule, which was accomplished using a finite mixture model of truncated binomials (see STAR Methods).

To demonstrate the application of mRNP-SiMPull, Nop56-PrA was used with Nop58-mNG or Snu13-mNG to isolate box C/D snoRNP complexes, within which Nop58 and Snu13 exist in a 1:2 ratio.^41^ Detected spots of Snu13 were brighter than Nop58 spots (Figure 1D), and uncorrected photobleaching step analysis data indicated a Snu13 dimer, which upon correction for reporter activity clearly reproduces the expected 1:2 ratio of Nop58 and Snu13 in a box C/D snoRNP complex (Figure 1E). Of note, a fraction of Nop58 and Snu13 spots showed higher stoichiometries, which are expected to represent molecular assemblies related to ribosome biogenesis.^42^

Together, the observed RNase sensitivity of analyzed complexes, requirement that RBPs are co-expressed in the same cell, stability of detected complexes, reproducibility of the data generated by different fluorescent reporters, and measurement of known stoichiometries demonstrate that mRNP-SiMPull provides information on RNPs formed *in vivo*.

### mRNP biogenesis and export factor occupancy in single mRNPs

With the ability to collect data on *in vivo* mRNP compositions, the occupancy of known mRNA biogenesis and export-related factors were tested by mRNP-SiMPull using Cbp80-PrA (member of the CBC) with mNG-tagged RBPs. It is important to note that the CBC remains bound to an mRNA post export until replacement by translation initiation factors,^43,44^ as these data will include mRNPs that represent stages of gene expression in both the nucleus and cytoplasm.

**Figure 2.**
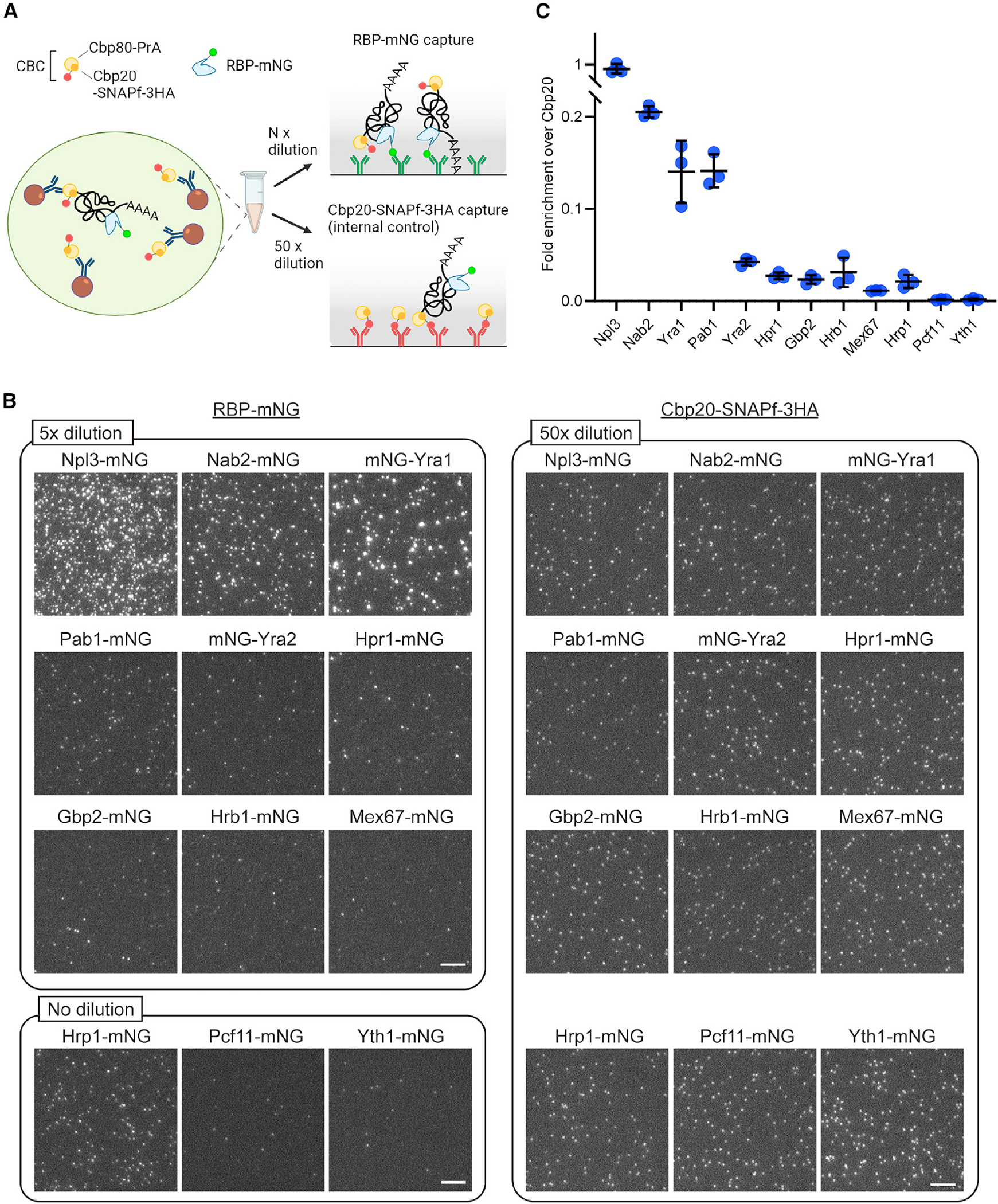
Occupancy of mRNA biogenesis and export-related RBPs in Cbp80-containing mRNPs (A) Cartoon summarizes the procedure to analyze the frequency of target RBPs in the population of CBC-containing mRNPs by mRNP-SiMPull. To perform these assays, RBPs were tagged with mNG in a strain with Cbp20-SNAP-3HA with/without Cbp80-PrA. IgG pull-downs were performed to enrich Cbp80-bound mRNPs and CBC itself. The elute was separately diluted and loaded into the mNG antibody coated (to capture RBP-mNG-bound mRNPs) and HA antibody coated (to capture Cbp20-SNAPf-3HA-bound mRNPs and free CBC complexes) slides. The spot number of RBP-mNG counts normalized by Cbp20-SNAPf-3HA counts was used for the comparison between the different RBPs. (B) Representative images used to determine the frequency of target RBP-containing mRNPs in the population of total Cbp80-bound mRNPs. See Figure S3 for comparison with untagged Cbp80 control strains. Scale bar, 5 μm. (C) Graph showing the spot number of RBP-mNG normalized by Cbp20-SNAPf-3HA value in the same sample. Mean and standard deviation are shown with individual data points from triplicate experiments. It was noted that mNG tagging of Yra1 and Pab1 caused a growth defect that was tag specific (Figure S3A), but these strains were used in this experiment to maintain consistency.

Comparing all RBPs (Figures 2 and S3 and Table S1), the SR-like protein Npl3 showed particularly high enrichment with Cbp80-PrA, approximately 4-fold higher than any other RBP. Upon RNase A treatment, most spots were lost for almost all RBPs tested, while ~50% of Npl3 spots were not RNase A sensitive (Figures 1B and S4). Npl3 was reported to directly bind the CBC,^45^ which likely accounts for the presence of a large fraction of RNase-insensitive Npl3 spots in a Cbp80-PrA pull-down. Still, even considering the RNase-insensitive fraction, Npl3 showed a high level of enrichment compared to other RBPs. This may reflect the existence of Npl3 in cytoplasmic mRNPs and/or non-coding RNPs (e.g., with small nuclear [sn]RNAs, small nucleolar [sno]RNAs, or long non-coding [lnc]RNAs), as Npl3 has functions in translation and binds non-coding RNAs.^46,47^ Based on the interactions of Npl3 with the CBC complex and its role in pre-mRNA capping quality control,^45^ it is also possible that Npl3 would be detected with Cbp80 in nascent and abortive transcripts that lack other RBPs found in mature mRNPs. It is expected that these multi-functional features of Npl3 account for the frequent association of Npl3 with Cbp80 within RNase-sensitive complexes.

Other RBPs strongly enriched with Cbp80 were Yra1 and the poly(A)-RNA-binding proteins Nab2 and Pab1 (Figures 2 and S3 and Table S1). In contrast, Yra2, the THO complex component Hpr1, and the SR-like proteins Gbp2 and Hrb1 were less frequently observed. Of the three cleavage- and polyadenylation-related RBPs, Yth1, Pcf11, and Hrp1 (components in CPF, CFIA, and CFIB complexes, respectively), only Hrp1 showed an appreciable level of enrichment. This raises the possibility that after the cleavage and polyadenylation reaction, Hrp1 remains associated with the mRNA and is exported to the cytoplasm with the mRNP. This is consistent with the observation that Hrp1 shuttles between the nucleus and cytoplasm and has a reported function in nonsense-mediated decay.^48,49^ The essential mRNA export receptor Mex67 was rarely observed. This supports recent works indicating that Mex67/NXF1 does not commonly join an mRNP in the nucleoplasm and is independently recruited to the NPC to mediate mRNP export.^50,51^ Overall, these data indicate that Npl3, Yra1, Nab2, and Pab1 commonly occupy Cbp80-containing mRNPs and that RBP occupancy is in line with protein expression levels^52^; i.e., highly expressed nuclear RBPs functioning in mRNA processing are more frequently bound to mRNPs (Table S1).

### RBP stoichiometry in single mRNPs

**Figure 3.**
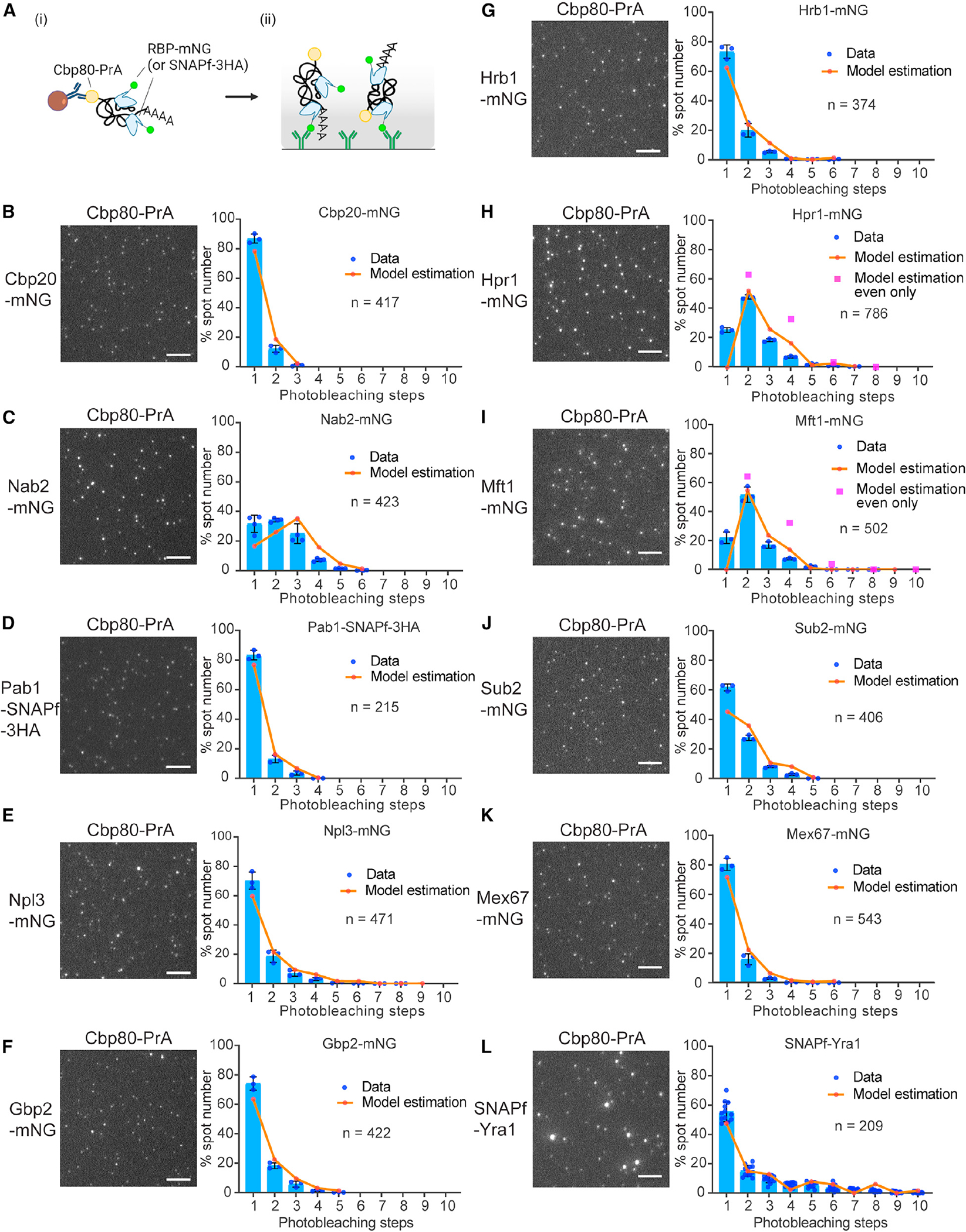
mRNA biogenesis and export-related RBP stoichiometry in CBC-containing mRNPs (A) Cartoon depicts the pull-down procedure for RBP-mNG- or -SNAPf-containing mRNPs in mRNP-SiMPull. Pull-down was performed by (i) IgG-beads followed by (ii) mRNP capturing via mNG, HA (for SNAPf-3HA), or Yra1 antibody on the glass surface. (B–L) Representative TIRF images of target RBPs (B: nuclear cap-binding complex component, Cbp20, C: nuclear poly A binding protein, Nab2, D: cytoplasmic poly A binding protein, Pab1, E: SR-like protein, Npl3, F: SR-like protein, Gbp2, G: SR-like protein, Hrb1, H: THO complex component, Hpr1, I: THO complex component, Mft1, J: RNA helicase, Sub2, K: mRNA export receptor, Mex67, L: mRNA export adapter protein, Yra1) obtained by mRNP-SiMPull from cell lysates co-expressing Cbp80-PrA (see Figure S5A for control images with untagged Cbp80 strains). Graphs display stoichiometry distributions determined by photobleaching steps analysis. Blue bars show mean data with standard deviation with dots showing individual data points in replicate experiments (12 and three replicates for L and the others, respectively). Orange line displays the expected stoichiometry distribution following correction for fluorescent reporter activity using finite mixture modeling. For Hpr1 (H) and Mft1 (I), magenta squares represent model estimation assuming a dimer as the base unit. Average number (n) of spots analyzed per replicate experiment is indicated on each graph. Image scale bars, 5 μm.

To address RBP stoichiometry in single mRNPs, copy number measures were obtained by photobleaching step analysis (Figure 3A), with the addition of the DEAD-box protein Sub2 and THO complex subunit Mft1 to the RBPs tested. In these assays, SNAP-tagged Yra1 and Pab1 were used due to an observed growth defect caused by mNG tagging (Figure S3A). All resulting stoichiometry data were corrected for reporter activity using a finite mixture model of truncated binomials (see STAR Methods). As expected for Cbp20, which forms a 1:1 complex with Cbp80,^53^ Cbp20 spots were uniformly dim, and ~80% of spots showed one-step photobleaching (Figures 3B and S5A). The cleavage and polyadenylation factors (Yth1, Pcf11, and Hrp1) also commonly had one molecule per mRNP (Figures S5B–S3D).

For the nuclear PABP Nab2, photobleaching data showed that one to six copies were detected within mRNPs (Figures 3C and S5A). This aligns with Nab2 binding ~25 adenines (As) *in vitro* and the measured length distribution of mRNA poly(A)-tails in yeast that have a mean and median poly(A)-tail length of 40 and 37 As, with lengths up to 140 As.^54,55^ Nab2 has also been shown to bind within the body of mRNAs and to form a dimer,^19,56^ which may contribute to these stoichiometries. In contrast, the mostly cytoplasmic PABP, Pab1, was most often present as one molecule per mRNP (Figures 3D and S5A). While Nab2 is the major nuclear PABP, the presence of Pab 1 on CBC-containing mRNPs isconsistent with Pab1 shuttling between nucleus and cytoplasm and contributing to poly(A)-tail length control and export.^57,58^ Moreover, the CBC remains bound to the mRNA post export until replacement by translation initiation factors for the pioneer round of translation,^43,44^ which may reflect the observed CBC-containing Pab1-bound mRNPs. It is reported that cytoplasmic degradation of translated mRNAs by the poly(A)-nuclease Pan complex requires more than two Pab1 molecules on the poly(A)-tail.^59^ The presence of a single copy of Pab1 in Cbp80-containing mRNPs suggests a mechanism that could distinguish and protect recently exported mRNAs from decay and favor translation.

The three SR-like proteins, Npl3, Gbp2, and Hrb1, showed similar stoichiometry distributions and were most often present as one copy in an mRNP; however, ~30%–40% of mRNPs contained two to six copies of these RBPs (Figures 3E–3G and S5A). This stoichiometric variation may reflect gene-specific mRNP compositions linked to SR-like protein functions in splicing (Npl3) and quality control (Gbp2 and Hrb1).^60–62^ In addition, it is known that Npl3 self-association is modulated by Npl3 methylation, and an Npl3-Npl3 interaction is required for monosome formation to activate translation,^63,64^ which may contribute to the observed stoichiometry.

THO complex components Hpr1 and Mft1 most frequently showed two-step photobleaching (Figures 3H, 3I, and S5A), with statistical modeling indicating that no observed mRNPs contained a single copy of Hpr1 or Mft1. This corresponds with reported structural data showing that the yeast THO complex forms a homodimer^65–67^ and confirms the THO complex is present on mRNA as a dimer *in vivo*. By limiting the statistical model to multiples of two, the data suggest that the THO complex is present as a single homodimer two-thirds of the time, with the remaining mRNPs most frequently having two homodimers present. This range of THO complex stoichiometry corresponds with estimates of the human tetrameric THO complex, which was recently modeled as one to three copies per mRNP.^35^ In the case of Sub2, a THO complex binding partner,^66–68^ an approximately equal ratio of mRNPs with one or two copies of Sub2 was indicated by the data (Figures 3J and S5A).

In the case of the major mRNA export factor, Mex67 (NXF1/TAP in humans),^69–71^ multiple adapter RBPs are known to aid association with the mRNP.^62,72–76^ Models likewise suggest multiple copies of Mex67 would be required for efficient transport through an NPC^5^; however, Mex67 stoichiometry was most often one per mRNP (Figures 3K and S5A). These data are in line with recent works that suggest Mex67 is not a stable component of mRNPs and associates transiently late in biogenesis with an mRNP at or near NPCs to mediate export.^50,51^ The majority of Mex67-bound Cbp80-containing mRNPs also contained Mtr2 (Figure S5F), proving the model that Mex67 is loaded into mRNPs as a Mex67-Mtr2 heterodimer.^70^

Finally, Yra1 showed a large variation in stoichiometry with ~50% of spots showing 2–10 copies (Figures 3L and S5A). The observed number of Yra1 molecules cannot be explained by assemblies containing multiple mRNPs, as recently reported in yeast,^77^ since most spots displayed Cbp20-mNG spot intensities consistent with a single copy of the CBC (Figure S5G). Previous work established that Yra1 does not actively shuttle between nucleus and cytoplasm^72,73^ and that Yra1 is removed from an mRNP prior to export via ubiquitination by Tom1^74^ and is further regulated by Dbp2.^78^ When mRNP-SiMPull was performed using a *tom1Δ* strain, or following depletion of Dbp2, photobleaching step analysis indicated Yra1 stoichiometries remained like the controls (Figure S6). These data indicate that the majority of mRNPs being analyzed are upstream of Tom1 function and that loss of Tom1 or Dbp2 activity does not perturb Yra1 loading into an mRNP. Yra2, a paralog of Yra1,^79^ showed a stoichiometry of mostly one molecule per mRNP (Figure S5E), consistent with Yra1 and Yra2 having distinct functions in mRNA biogenesis and export. Overall, these RBP stoichiometry and co-occupancy data provide an important quantitative framework to be considered with emerging structural data to inform models of mRNP architecture and mechanisms of nuclear export.

### Multicopy Yra1 mRNPs contain Nab2, Npl3, and Hpr1

**Figure 4.**
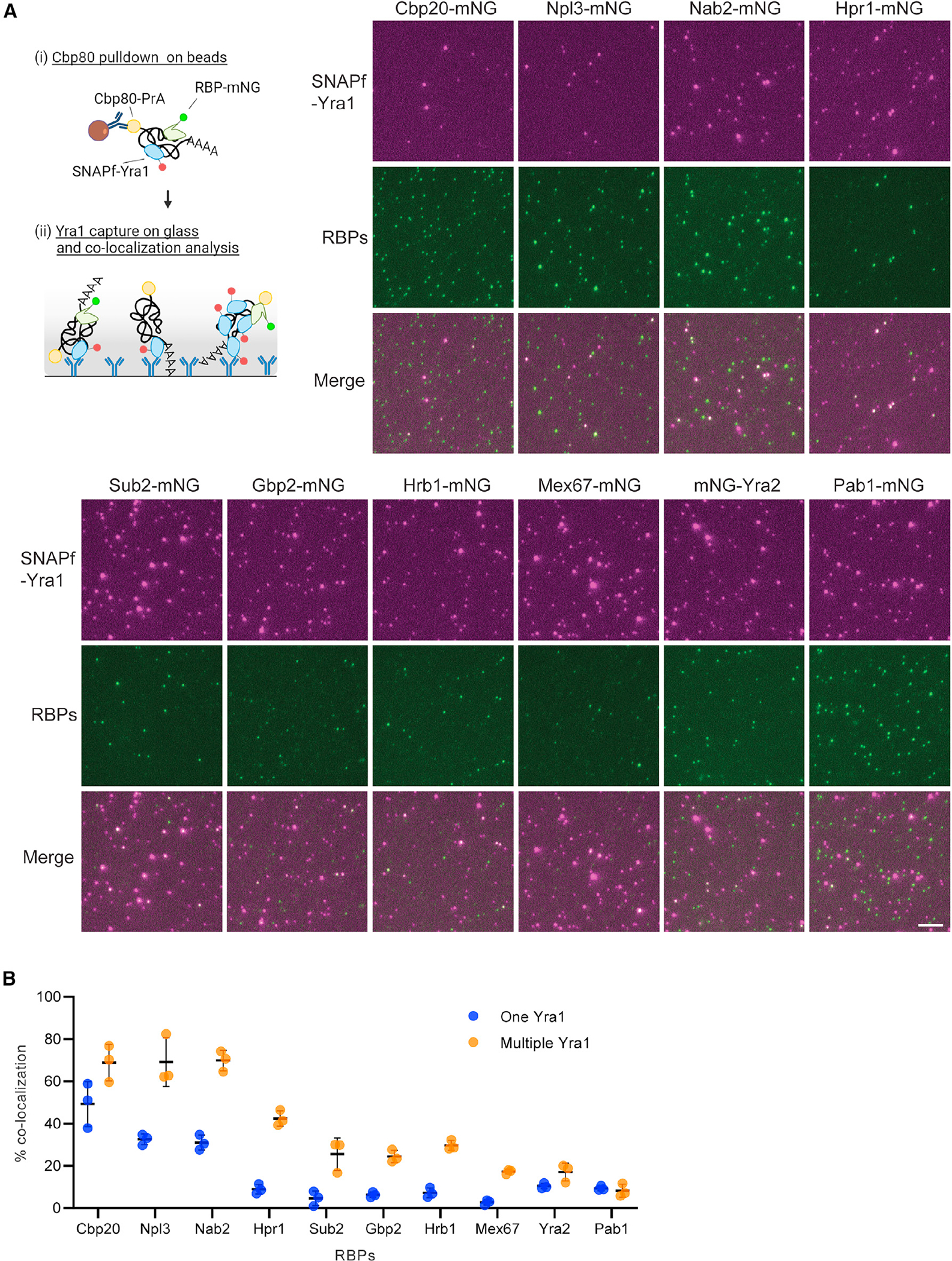
Co-localization analysis of Yra1 with other RBPs (A) Cartoon depicting the pull-down procedure for co-localization analysis of SNAPf-Yra1 and RBP-mNG by two-color mRNP-SiMPull. Pull-down was performed by (i) IgG-beads followed by (ii) mRNP capture via Yra1 antibody on the glass surface. Representative TIRF images used for co-localization analysis between SNAPf-Yra1 and other mNG-tagged RBPs. Scale bar, 5 μm. (B) Graph shows percent co-localization with one Yra1 vs. multiple Yra1 containing spots for the indicated RBPs. Yra1 spots were separated into two groups (one or multiple Yra1) based on spot intensity (see STAR Methods and Figure S7). The mean and standard deviation of percent co-localization calculated with fluorescent protein activity-uncorrected raw data for three replicate experiments are shown for each. Averaged spot numbers analyzed in each replicate for Cbp20, Npl3, Nab2, Hpr1, Sub2, Gbp2, Hrb1, Mex67, Yra2, and Pab1 images are 266, 202, 330, 359, 446, 281, 271, 309, 257, and 240 for one Yra1 and 126, 86, 195, 171, 233, 146, 165, 267, 181, and 173 for multiple Yra1s, respectively.

The observed range of Yra1 and other RBP stoichiometries reveals mRNP heterogeneity across the transcriptome and stages of gene expression. To further define the compositional nature of isolated Cbp80-containing mRNPs, co-occupancy of Yra1 with other RBPs was analyzed by two-color mRNP-SiMPull. For these assays, Cbp80-PrA pull-down material was loaded on glass slides functionalized with a Yra1 antibody to capture SNAPf-Yra1 complexes and determine co-localization with a mNG-tagged RBP (Figures 4A and S7A). Yra1-containing mRNPs were categorized as single or multicopy using measured intensities (Figures 4B, S7B, and S7C, see STAR Methods). Among these two groups, it was determined that single-copy Yra1 spots had a co-localization frequency of ~50% with Cbp20 (Figures 4 and S7A). Assuming Cbp20 is present as a single copy, combined with the measured mNG reporter activity (78%), these data indicate that approximately ~64% of single-copy Yra1 spots represent capped mRNAs. Co-localization between Cbp20 and multicopy Yra1 spots increased to ~70%, indicating that ~90% (based on reporter activity) of these spots are associated with the CBC.

Npl3 and Nab2 showed an uncorrected co-localization frequency of ~70% with multiple Yra1 spots, which decreased to ~30% for single Yra1 spots (Figure 4B). Hpr1 (~40%), Sub2 (~25%), Gbp2 (~25%), Hrb1 (~30%), and Mex67 (~20%) also showed biased co-localization to multicopy Yra1 spots with these RBPs showing a co-localization of ~10% or less to single Yra1 spots. Both Yra2 and Pab1 showed low levels of co-localization in all spots. In cases other than Cbp20, the data cannot be corrected for reporter activity due to a lack of knowledge about the populations these mRNPs are isolated from and their associated stoichiometry distributions. Consequently, these numbers represent a lower bound of RBP co-occupancy in mRNPs containing Yra1. The measured differences in RBP co-occupancy between single and multicopy Yra1 spots likely result from gene-specific mRNP heterogeneity, capturing mRNPs at different points in the gene expression pathway, and technical limitations of the approach (e.g., presence of free labeled protein). It is expected that multicopy Yra1 mRNPs with CBC, Nab2, Npl3, and THO complex are representative of a sub-population of mature nuclear mRNPs. Indeed, loss of any of these factors severely disrupts multiple aspects of gene expression,^38,39,47,58,60,61,68,72,75,80–82^ causing mRNA export defects and lethality in the case of Yra1 and Nab2.^39,72^

From the two-color mRNP-SiMPull data, spot intensity information was extracted to further investigate if the stoichiometries of different RBPs are correlated with Yra1 stoichiometry. Importantly, there is a strong relationship between intensity and measured stoichiometry within mRNP-SiMPull data (Figure S2F), which supports the validity of using intensity to infer stoichiometry in these analyses. Using this approach, no significant correlations related to copy number were identified among RBPs co-localizing with Yra1 (Figure S8). This suggests that Yra1 does not have a fixed binding partner among the tested RBPs that co-varies with respect to stoichiometry. These data are supported by recent crosslinking mass spectrometry data that showed the most frequent links involving Yra1 occurred between copies of Yra1, as well as a novel mRNP constituent Yhs7,^30^ which was not included in this study. Of the RBPs analyzed here, Npl3 shows some of the highest stoichiometry values after Yra1, and it was recently noted that Npl3 contains positively charged intrinsically disordered regions (IDRs) like Yra1, with IDRs in Yra1 promoting RNA-RNA interactions.^30^ These shared features (i.e., variable stoichiometry and IDRs) raise the possibility that Yra1 and Npl3, as multicopy constituents of mRNPs (this work), similarly promote RNA-RNA interactions on different sub-populations of mRNPs and/or act redundantly within the same mRNPs. Future work will employ RNA aptamers within the mRNP-SiMPull methodology to isolate gene-specific mRNPs, which will allow for an evaluation of gene-specific mRNP packaging networks and heterogeneity that exists involving Yra1, Npl3, and other RBPs.

### THO complex facilitates generation of multicopy Yra1 mRNPs

**Figure 5.**
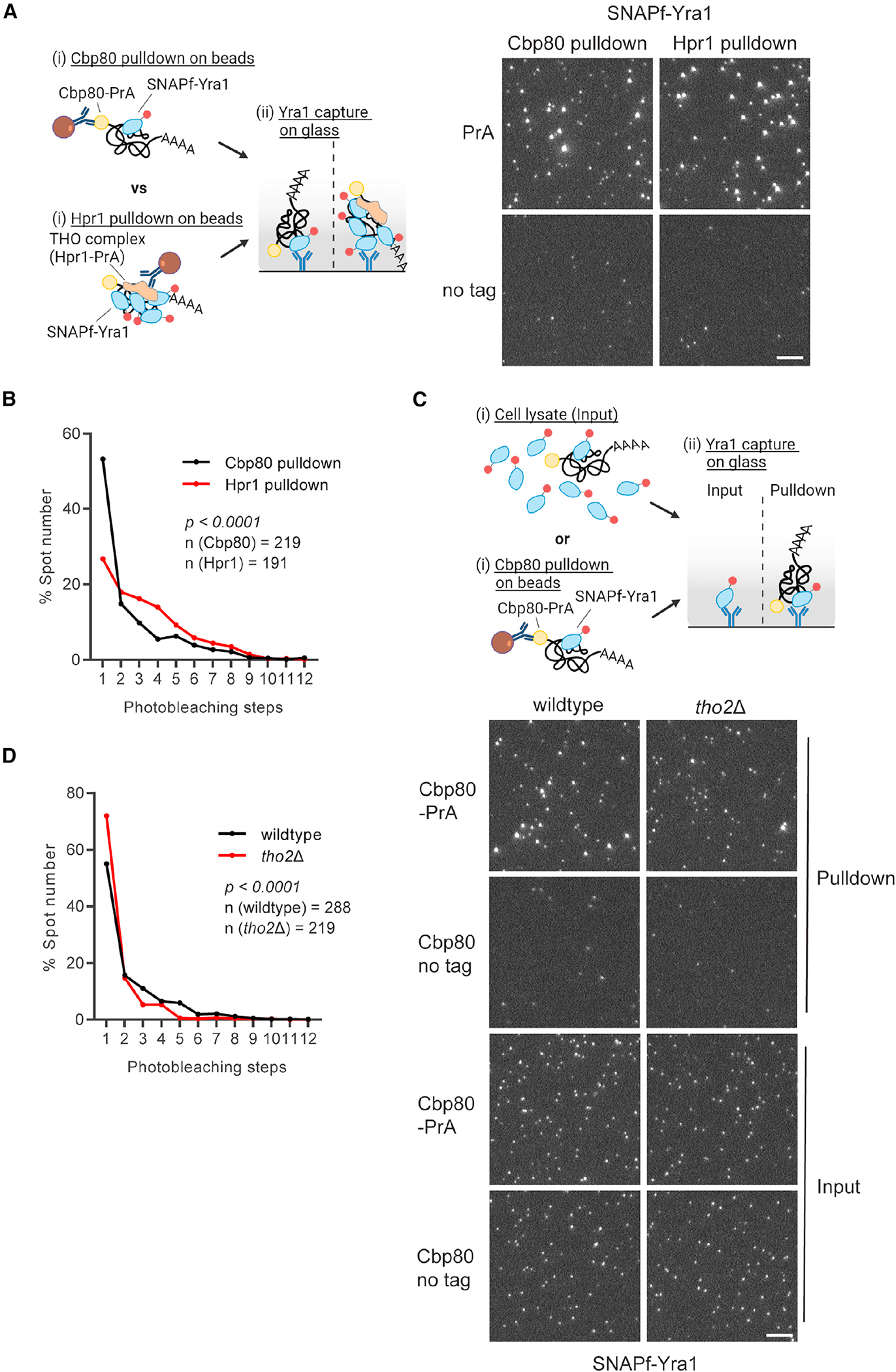
THO-dependent formation of multiple Yra1-containing mRNPs (A) Comparison of Yra1 stoichiometry distribution in Cbp80-PrA and Hpr1-PrA pull-down samples. Cartoon shows the pull-down procedure of mRNP-SiMPull by (i) IgG-beads to target Cbp80-PrA or Hpr1-PrA followed by mRNP capture via Yra1 antibody on the glass surface. Representative TIRF images of SNAPf-Yra1 obtained by mRNP-SiMPull from Cbp80-PrA and Hpr1-PrA pull-downs. (B) Line graph showing uncorrected raw mean photobleaching step data from triplicate experiments for SNAPf-Yra1 comparing Cbp80-PrA to Hpr1-PrA. p values were calculated by a non-parametric Kolmogorov-Smirnoff (KS) two-sample test. (C) Comparison of Yra1 stoichiometry in wild-type and *tho2*Δ strains by mRNP-SiMPull. Cartoon shows the pull-down procedure of mRNP-SiMPull by (i) IgG-beads for targeting Cbp80-PrA followed by (ii) mRNP capturing via Yra1 antibody on the glass surface. Cell lysates were loaded into the Yra1 antibody-coated glass slide for the analysis of input samples. Representative TIRF images of SNAPf-Yra1 obtained by mRNP-SiMPull from cell lysate (Input) and Cbp80-PrA pull-down samples in wild-type and *tho2*Δ strains. (D) Line graph shows uncorrected raw mean photobleaching step data from triplicate experiments for SNAPf-Yra1 comparing wild type to *tho2*Δ in Cbp80-PrA pull-down samples. p value is calculated by a non-parametric Kolmogorov-Smirnoff (KS) two-sample test. Average number (n) of spots analyzed per replicate experiment is indicated on each graph. Scale bar, 5 μm.

Co-localization analysis by two-color mRNP-SiMPull offers strong evidence for distinct types of Yra1-containing mRNPs. Current models suggest a major pathway for Yra1 loading is through the THO complex and Sub2 (as the TREX complex) involving Sub2 ATPase activity.^68,83–85^ The biased co-localization of Hpr1 (THO complex) and Sub2 with multiple Yra1-containing mRNPs (Figure 4) further suggests that TREX functions to load and/or stabilize Yra1 within mRNPs. To investigate this model, Hpr1-PrA was used in place of Cbp80-PrA to isolate mRNPs (Figure 5A), which resulted in a significant enrichment of multicopy Yra1 containing mRNPs (Figure 5B). Upon RNase A treatment of the Hpr1-PrA-associated material, most bright Yra1 spots were lost, confirming these are mRNPs, but dim spots were still present compared to a no-tag control (Figures S9A and S9B). Photobleaching step analysis of these RNase A-resistant spots showed mostly one-step bleaching (Figures S9C and S9D), which is consistent with Yra1 bound to the THO complex (i.e., part of the TREX complex) independent of RNA.^68^

To investigate the role of TREX in loading/stabilizing Yra1 within mRNPs, Yra1 stoichiometry was assessed with Cbp80-PrA in a *tho2*Δ strain, with *THO2* encoding the largest subunit of the THO complex. In a *tho2*Δ strain, photobleaching analysis revealed that multicopy Yra1 mRNPs declined from 45% to 28% of the population (~40% decrease) with a near complete loss of complexes with more than four copies of Yra1 (Figures 5C and 5D). These data demonstrate that THO complex supports multicopy binding of Yra1 to an mRNP, but the presence of Yra1 is not solely dependent on THO, as evidenced by total spot number and the persistence of multicopy Yra1 mRNPs. This corresponds with Yra1 being essential,^86^ while the THO complex is not,^68^ and the description of THO-independent Yra1 recruitment mechanisms that involve Pcf11, the RNA Pol II CTD, and interactions with other RBPs.^30,87,88^ It is also possible that Sub2 continues to engage Yra1 in the absence of the THO complex, but at a reduced efficiency, thus altering Yra1 stoichiometry. Strains carrying temperature-sensitive or auxin-induced degradation alleles of Sub2 were not successfully generated in the presence of tagged versions of Yra1 and Cbp80 that are needed for mRNP-SiMPull, leaving this possibility untested. These data demonstrate that the THO complex, likely in the form of TREX, is present in mRNPs and functions to generate and/or stabilize mRNPs with increased Yra1 stoichiometries.

### Transcript-specific features are correlated with Yra1 copy number

**Figure 6.**
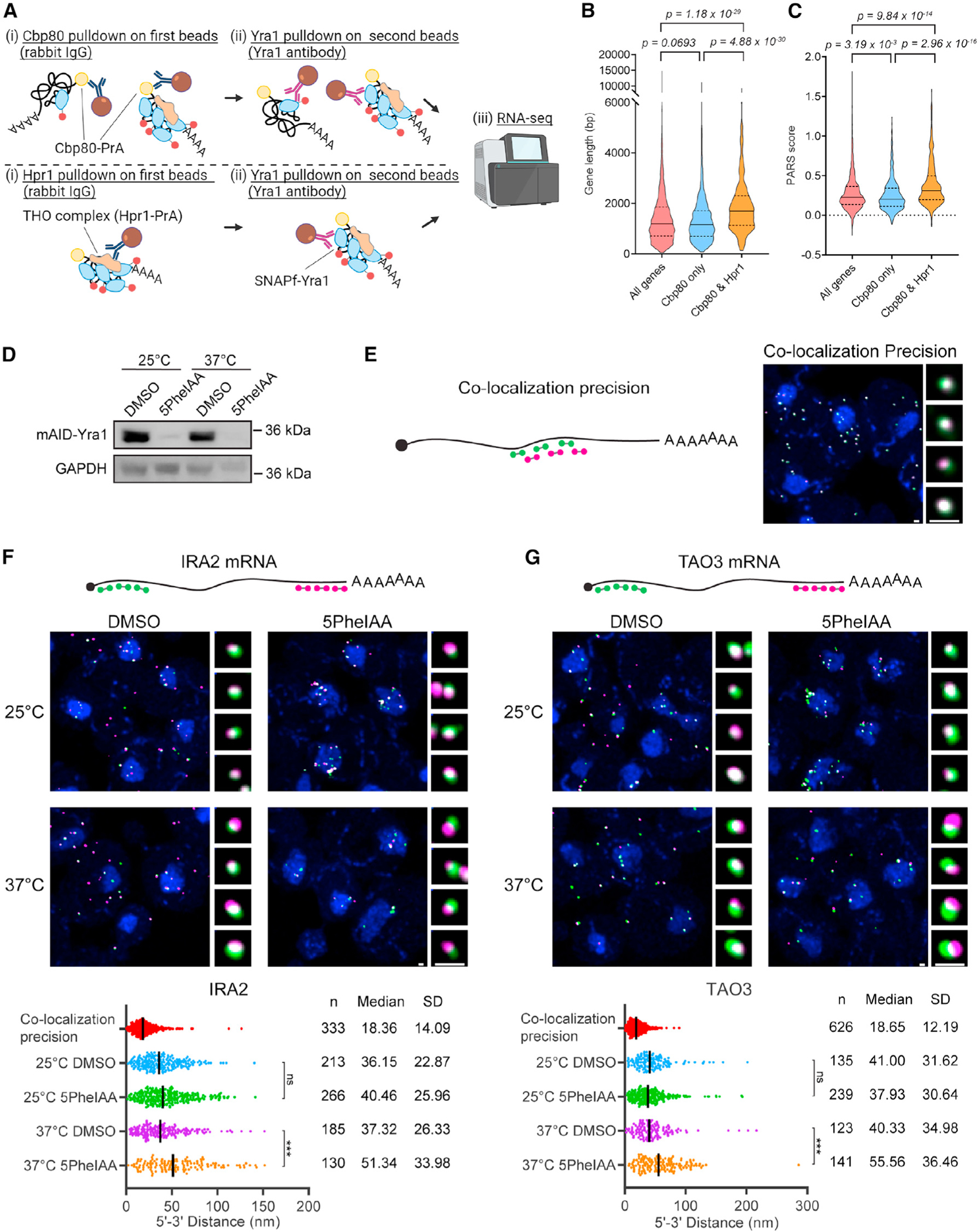
Yra1 is required for mRNP compaction (A) RNA-seq analysis to define mRNAs that form one and multiple Yra1-bound mRNPs. Cartoon shows the pull-down procedure for RNA-seq sample preparation. First pull-down was performed by (i) IgG-beads to target Cbp80-PrA or Hpr1-PrA. In the second pull-down (ii), Yra1-bound mRNPs were purified by Yra1 antibody-conjugated beads from which RNA was extracted for RNA-seq. (B and C) Violin plot showing gene length (bp) and preference to form secondary structure (average PARS score within each gene) of all annotated genes in *S. cerevisiae* vs. significantly enriched in only Cbp80/Yra1 (1,094 genes) or in both Cbp80/Yra1 and Hpr1/Yra1 pull-downs (522 genes). Median and quartile are shown as solid and dotted lines, respectively. p value was calculated by Wilcoxon’s rank-sum test. (D) Western blot shows Yra1 depletion by auxin-induced degron system after 2 h at the indicated temperatures. Protein size marker position is indicated at right side. (E–G) Illustrations show Atto647 and Alexa 594 smFISH probes used to target the same (E) or different (F and G) regions of target mRNAs for distance measurements by super-resolution STED imaging. Representative maximum projection images are shown, including four magnified examples from the nuclear volume for each sample. Scale bar, 400 nm. Dot plots display distances measured with probe sets targeting IRA2 (F) and TAO3 (G) mRNAs. Median and SD (standard deviation) are shown in nanometers. Statistic test was performed using Kolmogorov-Smirnov test. ns, not sensitive. ***p < 0.001.

Previous RNA binding data suggest Yra1 and the THO complex are associated with most gene transcripts, but Yra1 shows a propensity for longer transcripts.^20^ The THO complex has also been linked to the maintenance of genome stability via prevention of R-loop formation,^89^ which reportedly increases with gene length.^90,91^ Combining these findings with the data presented here, a putative model is that Yra1 is loaded on transcripts in a length-biased manner by TREX to support mRNP packaging, which upon disruption increases R-loop formation and genome instability. An expectation of this model is that THO complex-bound mRNPs would be biased to longer transcripts. Thus, RNA-seq analysis was performed on material isolated from two-step pull-downs that targeted Cbp80-PrA vs. Hpr1-PrA in the first step and selected for Yra1 in the second step (i.e., Cbp80/Yra1 vs. Hpr1/Yra1) (Figure 6A). This analysis identified 1,616 (Cbp80/Yra1) and 753 (Hpr1/Yra1) genes that were significantly enriched over a no-tag control sample with 522 genes in common (Figure S10A). The genes in common to both Cbp80/Yra1 and Hpr1/Yra1 two-step pull-downs, which are expected to be enriched for multiple Yra1-containing mRNPs, were significantly longer than the genome average or those only enriched by Cbp80/Yra1 (Figure 6B). Upon performing the same Cbp80/Yra1 pull-down in a *tho2*D strain, a statistically significant loss of longer transcripts was observed (Figure S10B), which is suggestive of a loss of Yra1 from these transcripts and a decreased pull-down efficiency. These data are consistent with transcript length being a feature associated with THO complex-bound mRNPs and increased Yra1 copy number.

Since Yra1 has robust RNA-RNA annealing activity,^86^ RNA secondary structure was also investigated as a feature linked to Yra1 stoichiometry utilizing published parallel analysis of RNA structure (PARS) data.^92^ PARS data in *S. cerevisiae* provides *in vitro* data on the propensity of nucleotides within a transcript to be present in a double- or single-stranded conformation, with positive PARS values indicating nucleotides more commonly in a double-strand RNA conformation. The averaged PARS score in each gene was compared, and it was found that genes enriched in both IPs showed significantly higher PARS score than the genome average or genes enriched only by Cbp80/Yra1 (Figure 6C). This indicates that transcripts enriched by both Cbp80/Yra1 and Hpr1/Yra1 (i.e., multiple Yra1-containing mRNPs) have sequences with a higher potential to form a secondary structure. In addition, genes enriched in both IPs were generally found to be expressed at higher levels, have increased synthesis and decay rates,^93^ and lack introns (Figures S10C–S10F). The anti-correlate observed between the THO complex and spliced mRNAs matches reports of intron-containing mRNAs being less sensitive to loss of THO complex function.^94^ These findings suggest that Yra1 stoichiometry is linked to transcript features important for mRNP packaging, including transcript length and RNA secondary structure.

### Yra1 loading is required for stable compaction of nuclear mRNPs

Nuclear mRNPs form compacted particles.^23,25,30,31,35^ Given the correlation of Yra1 copy number with transcript length and secondary structure, Yra1 RNA-RNA annealing activity,^86^ and the extensive protein-protein and protein-RNA network Yra1 engages in,^30^ it is likely that Yra1 function is related to the maintenance of mRNP compaction. To examine this hypothesis, nuclear mRNP compaction was analyzed by measuring the distance between the 5′ and 3′ region of a target mRNA using smFISH and super-resolution STED imaging. Considering the importance of temperature to RNA folding, these data were collected at 25°C and 37°C with and without auxin-induced depletion^95^ of Yra1 (Figure 6D). Two mRNAs, *IRA2* (9,240 nt) and TAO3 (7,131 nt), were selected as mRNAs that are enriched in the RNA-seq datasets generated from Cbp80/Yra1 and Hpr1/Yra1 pull-downs. To determine the co-localization precision of this approach, a set of differentially labeled alternating smFISH probes targeting an ~2-kb region within a control mRNA (*MDN1*) were used. STED imaging of the overlapping probe sets showed a high degree of co-localization with a median distance between the two labeled spots of ~18 nm, indicating the co-localization precision of this setup (Figures 6E–6G). At 25°C, depletion of Yra1 caused a robust mRNA export defect for both targets, but 5′–3′ spot distances (40 nm for IRA2 and 38 nm for TAO3) were not significantly changed compared to control (36 nm for IRA2 and 41 nm for TAO3; Figures 6F, 6G, S10G, and S10H). In contrast, spatially separated 5′ and 3′ spots were frequently observed upon depletion of Yra1 at 37°C with a median distance (51 nm for IRA2 and 56 nm for TAO3) that was significantly increased compared to control (37 nm for IRA2 and 40 nm for TAO3; Figures 6F, 6G, S10G, and S10H). Notably, in the absence of Yra1 at 37°C, the number of mRNAs per cell was strongly diminished compared to 25°C (Figures 6F and 6G), suggesting changes in mRNP packaging are likely accompanied by increased nuclear decay and/or decreased transcription. These data indicate that with increased temperature, Yra1 becomes essential to maintaining mRNP compaction and gene expression.

**Figure 7.**
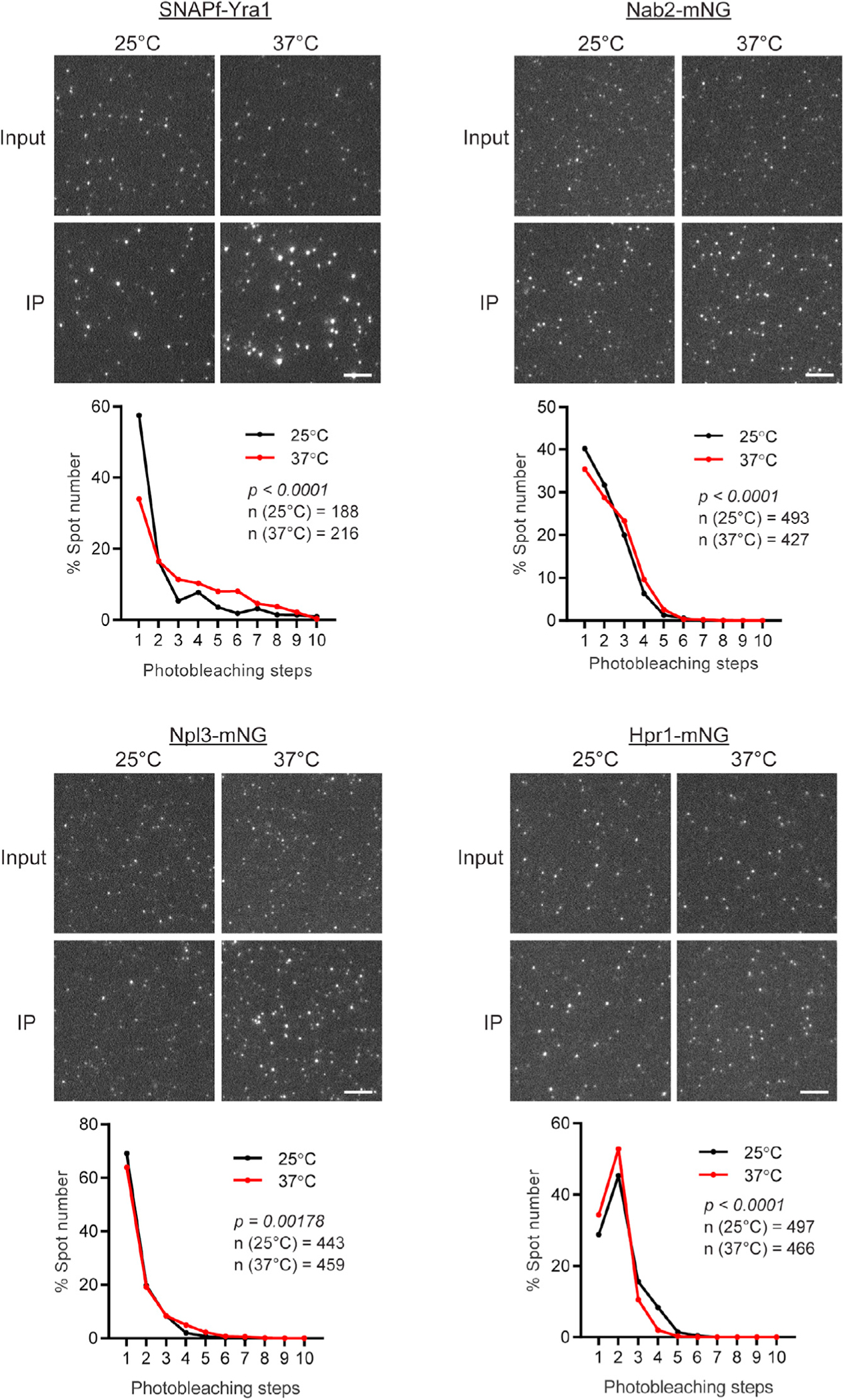
RBP stoichiometry in mRNPs is altered by cell growth temperature Representative TIRF images of SNAPf-Yra1, Nab2-mNG, Npl3-mNG, and Hpr1-mNG obtained by mRNP-SiMPull from cells grown at 25°C or 37°C for 2 h. IP (Cbp80-PrA pull-down) and input (cell lysate sample) images are shown. Scale bar, 5 μm. Line graphs show uncorrected raw mean photobleaching step data from triplicate experiments for 25°C and 37°C. p values were calculated by a non-parametric Kolmogorov-Smirnoff (KS) two-sample tests. Average number (n) of spots analyzed per replicate experiment is indicated on each graph.

Temperature is a significant determinant of RNA annealing, and it isreported that the thermal stability of secondary structure within mRNA is relatively lower than non-coding RNAs.^96^ As such, it is possible that changes in growth temperature need to be buffered by changes in RBP stoichiometry to maintain mRNP packaging and gene expression. A failure to do so may result in an inability to maintain mRNP packaging and proper gene expression, as observed upon depletion of Yra1 at 37°C (Figure 6). To evaluate whether RBP stoichiometries vary with growth temperature, Nab2, Npl3, Yra1, and Hpr1 stoichiometries were compared across yeast cultures grown at 25°C, 30°C, and 37°C (Figures 7 and S11). Of the tested RBPs, photobleaching step analysis indicated the stoichiometry distribution of Yra1 was dramatically altered by temperature, showing a rise in multicopy Yra1 mRNPs as temperature increased, which was accompanied by a ~50% decrease in the population of single-copy Yra1 mRNPs at 37°C. Npl3 and Nab2 copy number were also increased at 37°C, which in the case of Nab2 may reflect the reported lengthening of mRNA poly(A)-tails at 37°C.^55^ In contrast, it was observed that Hpr1 copy number decreased at 37°C, highlighting that not all RBP stoichiometries are increased with temperature. Importantly, material captured on slide directly from lysate had RBP spot intensities that were indistinguishable between temperatures, suggesting increased stoichiometries were not the result of protein aggregation. Transcriptome analyses of yeasts grown at 25°C, 30°C, and 37°C showed that no genes were differentially expressed between 25°C and 30°C, and only 40 and 11 genes were differentially expressed after 2 h of growth at 37°C when compared to 25°C or 30°C cultures (Table S2). This shows that mRNP compositions are changing in response to growth temperature, and these changes are not the result of an altered transcriptome. These data demonstrate that mRNP composition is regulated in response to growth temperature with the same transcripts adopting different RBP compositions to support gene expression.

### Conclusions

Here, quantitative measures of mRNP composition at the single-molecule level are provided by mRNP-SiMPull. These data highlight the plasticity of mRNPs, with compositions that are gene feature dependent and responsive to cellular growth conditions (Figure S12A). The data reveal that Yra1 (Aly/REF) is present in individual mRNPs from 1 to 10 copies, and it interacts with mRNAs in a manner biased by length and RNA secondary structure. Yra1-containing mRNPs also commonly contain the poly(A)-RBP Nab2 and SR-like protein Npl3, and in association with THO/TREX complex, Yra1 stoichiometry is increased (Figure S12B). Given the expectation that the CBC is rapidly replaced post nuclear export by translation initiation factors^44^ and that Yra1 does not shuttle between nucleus and cytoplasm,^72,73^ these data indicate that CBC, Npl3, Nab2, and Yra1 form core components of nuclear mRNPs. Furthermore, at least for *IRA2* and *TAO3*, Yra1 is required for the establishment and/or maintenance of a compacted mRNP structure at 37°C (Figure 6). This role of Yra1 as an organizer of mRNP structure aligns with the original identification of Yra1 as a factor with robust RNA annealing activity.^86^ Given that the mouse ALY gene can complement the lethality of a *YRA1* loss of function mutant in *S. cerevisiae*,^72^ it is likely this function is conserved among Yra1 orthologs of the Opisthokonta supergroup.

Recently, crosslinking mass spectrometry analysis of yeast mRNPs purified via the THO complex identified intermolecular interactions between copies of Yra1 and between Yra1 and the other RBPs including Nab2.^30^ In addition, it was demonstrated by Bonneau et al. that positively charged IDRs in Yra1 and the THO complex subunit Tho2 promote RNA annealing, with IDRs also identified in other RBPs, including Npl3 and Yra2. This work using mRNP-SiMPull has shown that individual capped mRNPs containing the THO complex and Yra1 are also frequently co-occupied by Npl3 and Nab2 (Figures 3, 4, and 5). In humans, recent cryo-EM analyses have similarly suggested a critical role for Aly/REF (Yra1) in organizing mRNPs through multivalent protein-RNA and protein-protein interactions that involve bridging multiple TREX and EJC complexes.^35^ Although yeast lack an EJC,^34^ the central role of Yra1(Aly/REF) in both yeast and humans appears to be conserved. Specifically, both Yra1 and Aly/REF play a critical role in mediating intermolecular RBP interactions to organize compacted mRNPs, which is achieved through forming mRNPs with varying RBP stoichiometries. Combing these findings with data presented here, we propose that Yra1 is a critical conserved mRNP organizer, acting with other RBPs (e.g., CBC, Npl3, Nab2, Sub2, and the THO complex) in a regulated manner to generate compact and export-competent mRNPs.

### Limitations of the study

This study highlights mRNP compositions and heterogeneity linked to transcript features but is limited by the fact that the identity of the transcript within each mRNP analyzed is not known. Future work will need to characterize gene-specific mRNP compositions, which will be influenced by differences in mRNA processing (e.g., splicing) and gene expression regulation (e.g., transcription factor identity, cellular state, and stress). These questions may be addressed by altering the mRNP-SiMPull methodology to use mRNA aptamers (e.g., MS2) in one of the isolation steps to capture gene-specific mRNPs. In addition, it will be important to understand changes in mRNP composition across mRNA biogenesis and export, including transient and low-abundance configurations. By using Cbp80 to enrich nuclear mRNPs, this work reflects complexes across gene expression, including mRNPs post nuclear export, and likely represents the most frequent configurations (i.e., slow kinetic steps) within the process. Similarly, measured RBP stoichiometries reflect heterogeneity resulting from gene-specific difference, capturing mRNPs at different points in the gene expression pathway, which may include nascent transcripts or decay products, and technical limitations of the approach (e.g., presence of free labeled protein). In the future, it may be possible to identify further mRNP intermediates, and extend stoichiometry measurements, across the gene expression pathway by targeting a broader repertoire and combination of RBPs for enrichment. In addition, mutants could be employed to accumulate biogenesis intermediates with the caveat that mutants will also disrupt the gene expression program and generate alternate or aberrant mRNPs due to the induced cellular perturbation.^97–100^ It is expected that future use, and variations, of mRNP-SiMPull could provide these types of data to advance models of post-transcriptional gene regulation.

## STAR★METHODS

### RESOURCE AVAILABILITY

#### Lead contact

Further information and requests for resources and reagents should be directed to and will be fulfilled by the lead contact, Ben Montpetit (benmontpetit@ucdavis.edu).

#### Materials availability

All materials generated in this study such as plasmids and yeast strains are available from the lead contact.

#### Data and code availability

- RNA-seq data have been deposited at GEO and are publicly available as of the date of publication. Accession numbers are listed in the key resources table. Original western blot images have been deposited at Zenodo and are publicly available as of the date of publication. The DOI is listed in the key resources table. Microscopy data reported in this paper will be shared by the lead contact upon request.
- All original code has been deposited at Zenodo and is publicly available as of the date of publication. DOIs are listed in the key resources table.
- Any additional information required to reanalyze the data reported in this paper is available from the lead contact upon request.

### EXPERIMENTAL MODEL AND STUDY PARTICIPANTS DETAILS

Yeast strains used in this study (Table S3) were derived from *S. cerevisiae* BY4741/2. Cells were grown in YPD media at 30°C unless otherwise stated. All strains were validated by a combination of colony PCR, PCR from isolated genomic DNA, western blotting, and microscopy.

### METHOD DETAILS

#### Yeast strains, plasmids and oligos

Transformations were performed by LiOAc based method.^111^ Plasmids and primers used in this study are listed in Tables S4 and S5, respectively. C-terminal tagging was carried out using PCR based integration strategy.^112^ N-terminal tagging was conducted by homologous recombination using plasmid constructs specific to each gene being targeted. For depletion of an essential gene, the auxin induced degron 2 (AID2) system was employed.^95^ For *THO2* and *TOM1* gene deletions, a gene deletion fragment containing a KanMX marker was PCR amplified from the genome of deletion clones within the yeast deletion collection.^113^ Plasmids used in the AID system (BYP7425 and BYP9795) were provided by the National Bio-Resource Project (NBRP), Japan. PCR templates for tagging and primers are listed in Tables S4 and S5.

#### Spot assay

Overnight cultures of yeast cells were diluted to OD_600_ = 0.1 and cultured until OD_600_ = ~1. 5-fold serial dilutions were prepared from OD_600_ = 0.1 for spotting on plates. Plates were incubated at 30°C for 1–2 days.

#### mRNP-single-molecule-pull-down (mRNP-SiMPull)

##### Yeast culture and cryo grinding

Yeast strains were cultured from initial starter cultures in 500 mL of YPD at 30°C to OD_600_ of ~1.0. For the analysis of the *tom1*Δ strain and different growth temperature (for 25°C), cells were cultured at 25°C. To analyze the mRNP composition in 37°C, cell culture grown at 25°C in 250 mL at OD_600_ ~1 were mixed with equal volume of pre-warmed medium and initially incubated in 37°C water bath for 15 min, followed by culturing in 37°C shaker for totally 2 h. To deplete mAID tagged protein, final 1 μM of 5PheIAA (Bio Academia, #30–003) or same volume of DMSO solvent were added to the culture at OD_600_ ~0.6 and it was cultured for 2 h. Cryo grinding was performed as described.^114^ Cells were harvested by centrifugation at 2600 rcf, 5 min, 4°C. After washing with cold DI water twice, cells were resuspended by resuspension buffer (20 mM HEPES-KOH pH7.4, 1.2% PVP-40, 4 μg/mL Pepstatin A, 0.18 mg/mL PMSF, 1 mM DTT). Centrifuged twice at 2600 rcf, 15 min, 4°C to completely remove the buffer. The cell pellets were transferred to 10 mL syringe and press out the cells into the liquid nitrogen to freeze them. Frozen cell pellets were ground using a TissueLyser II (QIAGEN) @ 30 Hz for 2 X 15 s.

##### Preparation of antibody coated TIRF slides

Glass coverslips (No. 1.5H thickness, Merienfeld cat #0107032) were washed as described.^115^ To build TIRF slide chamber, a washed coverslip stored in 95% ethanol was flamed for 30 s and sealed to a glass slide using double sided tape. A poly-L-lysine-PEG-biotin solution (0.5 mg/mL PLL(20)-g[3.5]-PEG(2)/PEG(3.4)-biotin 20% (SuSoS), 20 mM HEPES-KOH pH 7.4) was loaded into the chamber and incubated for 10 min at room temperature. A streptavidin solution (0.2 mg/mL streptavidin (Prozyme, SA10), 20 mM HEPES-KOH pH 7.4, 12.5 mM KOAc, 2 mM MgCl_2_) was then loaded into the biotin coated chamber and incubated for 2 min at room temperature. The streptavidin coated chamber was washed with 20 μL of TIRF wash buffer with BSA/Casein (20 mM HEPES-KOH pH 7.4, 12.5 mM KOAc, 2 mM MgCl_2_, 1 mg/mL BSA, 1 mg/mL Casein). To immobilize antibodies, first, a biotinylated secondary antibody (anti-rabbit-biotin (Vector Lab, BA-1000) or anti-mouse-biotin (Invitrogen, 31800)) in solution (~20 nM antibody, 20 mM HEPES-KOH pH 7.4, 12.5 mM KOAc, 2 mM MgCl_2_, 1 mg/mL BSA, 1 mg/mL Casein) was loaded and incubated for 10 min at room temperature. Then, following washing by 20 μL of TIRF wash buffer with BSA/Casein twice, primary antibody (anti-mNG (chromotek, 32F6), anti-HA (Sigma, H3663) or anti-Yra1 (a kind gift from Dr. Doug Kellogg, UC Santa Cruz) in solution (~10 nM antibody, 20 mM HEPES-KOH pH 7.4, 12.5 mM KOAc, 2 mM MgCl_2_, 1 mg/mL BSA, 1 mg/mL Casein) was loaded and incubated for 20 min. The antibody coated chamber was washed twice by 20 μL of blocking solution (20 mM HEPES-KOH pH 7.4, 12.5 mM KOAc, 2 mM MgCl_2_, 0.5% Pluronic F-127, 0.1 mg/mL BSA, 0.2 mg/mL κ-casein) and then cooled in ice water filled and foil covered tip box in an 8°C fridge until the pulldown sample was ready.

##### mRNP pulldown and TIRF imaging

Frozen yeast cell grindate was suspended in chilled mTBT16 buffer (20 mM HEPES-KOH pH 7.4, 12.5 mM KOAc, 2 mM MgCl_2_, 0.5% Triton X-100, 0.1% Tween 20, 0.18 mg/mL PMSF, 4 μg/mL Pepstatin A, 1 mM DTT, 1:5000 RNase inhibitor murine (NEB, M0314L), 1:5000 Antiform B (Sigma, A5757)) at a concentration of 10 mg per 1 mL buffer. A volume of 150 μL of grindate/buffer suspension was collected into a 1.5 mL tube and SNAP-549 (NEB, S9112S) or −649 (NEB, S9159S) dye was added to 10 μM when required. For the analysis of RNase sensitivity, RNase A (final concentration 10 μg/mL) was added at this step. Cell lysate was clarified by centrifugation for 5 min at 18900 rcf and 8°C. To the 125 μL of cleared lysate, formaldehyde was added to be final concentration 0.2%, incubated for 5 min on ice, and crosslink was stopped by then adding glycine to 100 mM. To the tube, ~187.5 μg of Dynabeads conjugated with rabbit IgG were added after being washed in cold mTBT16 buffer three times. The pulldown reaction was done with rotation at 8°C for 10 min followed by supernatant removal and bead washing with cold mTBT16 buffer without protease inhibitors three times. Washed beads were resuspended in 25 μL of mTBT16 buffer without protease inhibitors and HRV3C protease (AG Scientific, H-1192) was added to a concentration 0.01 mg/mL and incubated with rotating at 8°C for 5 min. The supernatant was diluted in TIRF dilution buffer (20 mM HEPES-KOH pH 7.4, 12.5 mM KOAc, 2 mM MgCl_2_, 0.5% Triton X-100, 0.1% Tween 20, 0.5% Pluronic F-127, 0.1 mg/mL BSA, 0.2 mg/mL κ-casein) supplemented with anti-photobleaching and blinking reagents (0.15 mg/mL Catalase (Sigma, C-40), 0.42 mg/mL Glucose oxidase (Sigma, G2133), 4.5 mg/mL Glucose and 2 mM Trolox (Sigma, 238813)). Diluted IP samples were loaded into the antibody coated glass chamber and incubated in an ice water filled and foil covered tip box within an 8°C fridge for 10 min. The chamber was then washed by 20 μL of TIRF dilution buffer with anti-photobleaching and blinking reagents twice. After wiping the surface of glass coverslip, the slide was set on the microscope and TIRF imaging performed using a Leica 63×1.47 NA TIRF objective on a DMi8 stand connected to an Andor Dragonfly microscope and an iXon Ultra 888 EMCCD camera.

For the cell lysate mixing experiment, two cell grindates were individually prepared, resuspended in mTBT16 buffer, and then mixed in a 1:1 ratio for the pulldown procedure. In case of mixing experiment of Cbp80-PrA and Nab2-mNG, final IP product of mixed sample was diluted to two times higher concentration than the other samples (Cbp80-PrA/Nab2-mNG co-expressed and Nab2-mNG only) to be the same amount of background fluorescently positive protein. For the assessment of mRNP stability, an additional 50 min incubation time at 8°C was added prior to protease cleavage, which was followed by normal elution, processing, and imaging.

To compare RBP frequencies across Cbp80 pulldowns, yeast strains with Cbp20-SNAPf-3HA were used. Cbp20-SNAPf-3HA was partially labeled using 1 μM of SNAP-649 dye and pulldown was performed. The eluted pulldown product was independently diluted for each pulldown to achieve an RBP and Cbp20 spot density that allowed separated spots to be observed, which were loaded into imaging lanes coated with an mNG or HA antibody. TIRF imaging was performed with an exposure time of 250 ms, a camera EM gain of 100, laser power @ 10%, with a 150 mW 488 nm or 140 mW 637 nm excitation laser and filters selected for mNG (525/50 nm) or SNAP-649 (700/75 nm), respectively. Spot detection was carried out using CoSMoS analysis software, imscroll (https://github.com/gelles-brandeis/CoSMoS_analysis), as described.^37,102^ The spot detection parameter was determined individually for each fluorescent protein and imaging setting by comparing fluorescent protein +/− reporter strain images to minimize background noise detection without loss of true fluorescent protein signals. Spot numbers counted in Cbp80-PrA samples were corrected for background signals (e.g., non-specific binding of free RBP-mNG protein molecules) via subtraction of spots detected in a Cbp80 no tag sample. Considering the number of detected spots with the dilution factor, a total number of spots was calculated and then normalized based on the Cbp20-SNAPf-3HA spot number. Three biological replicates were analyzed.

To check if the protein conditions (e.g., aggregation) are same in the different conditions (i.e., mutants or different temperatures) in the cell lysate, input samples (cell lysate) were imaged as well as pulldown samples. After formaldehyde crosslinking, 1 μL of the lysate was aliquoted to a tube containing 99 μL of TIRF dilution buffer and kept on ice until pulldown samples were ready. With additional dilution as needed, the input samples were loaded into the TIRF glass chamber and imaged.

##### Photobleaching step analysis

For stoichiometry estimations, pulldowns were as described except for Sub2-mNG that showed non-specific binding to the beads. To bypass this issue, a strain with Cbp20-SNAPf-3HA was used with Sub2-mNG so that mRNPs were captured on the glass surface via Cbp20-SNAPf-3HA using an HA antibody. For SNAP tag labeling in photobleaching step analysis, SNAP-surface 549 was used. In all cases, pulldown products were individually diluted to get a spot density that minimized chances of overlap in the spot detection process. To collect photobleaching data, a TIRF imaging time series was performed until most of the spots were photobleached, typically 550–2000 frames for mNG and SNAP-549. For mNG tagged RBPs, a 150 mW 488 nm excitation laser and 525/50 nm emission filter were used with 250 ms exposures, an EM gain of 100, and a laser power of 10%. For SNAP-549 labeled RBPs, a 100 mW 561 laser and 600/50nm filter set was used with 250 ms exposures, an EM gain of 100, and a laser power of 10%. Spot detection was as described above with any spots overlapping within a square 7×7 pixel region manually removed to avoid interference with the intensity trace by a neighboring spot. Corrections for stage drift and tracing of spot intensity over time were performed using imscroll as described before.^37,102^ Photobleaching step counting was performed using the AGATHA CPS program as described.^103^ To correct for the detection of background spots (e.g., dust and non-specific RBP binding), spot data from a non-tagged control sample is subtracted from the experimental sample data. After removing zero counts, the percentage of spots in each photobleaching step was calculated. At least three biological replicates were analyzed for each RBP.

##### Co-localization analysis

Yeast strains harboring both a mNG or SNAPf tagged RBP with or without PrA tagged Cbp80 genes were used for pulldown as described above. For co-localization analysis, SNAP surface-649 dye was used for labeling of SNAPf tag. Single frame two-color TIRF imaging was performed using 250 ms exposures, an EM gain of 100, and laser powers of 60% for the 488 nm line and 10% for 637 nm line. A glass slide with fluorescent beads (TetraSpeck, Invitrogen, T7279) was also imaged to correct for aberrations between the two channels. To analyze co-localization, spots position in one of the images was assigned, optical aberrations corrected and mapped to the second channel using the imscroll mapping function, and the intensity of a 2×2 pixel area acquired in the second channel. Using an intensity histogram, manually set thresholds were made to separate empty spots and co-localized spots, which were quantified. As with photobleaching step data, a no-PrA tag control strain was used to generate data, and this was subtracted from the experimental data to control for false positive co-localization events caused by background spots. From these data, the percentage of co-localized spots were calculated for three biological replicates.

To analyze co-localization for one vs. multicopy Yra1 spots, detected SNAPf-Yra1 spots were separated based on spot intensity. To achieve this, a histogram of SNAPf-Yra1 spots was generated and a manually selected cut-off was used to identify the low intensity peak mostly containing one step photobleached molecules (Figures S7B, S7C). Co-localization was analyzed as described above for these two populations.

For the analysis of co-localized RBP spot intensity, first, spot position in both channel of the images was assigned. Optical aberrations in RBP-mNG and SNAPf-Yra1 image were corrected to allow the comparison of spot coordinate with SNAPf-Yra1 channel image as described above. The spot coordinate and 7×7 pixel area intensity were acquired in both channels. For SNAPf-Yra1 spots, the distance to the all assigned RBP-mNG spots was calculated and the closest RBP-mNG spot was defined. The SNAPf-Yra1 spots with a co-localized RBP-mNG spots were identified as spots that have an RBP-mNG spot within 3 pixels. The log and rank transformed spot intensity of the pair of co-localized SNAPf-Yra1 and RBP-mNG spots were plotted and the correlation was tested by generalized additive regression model (GAM) of Yra1 with the other RBPs.

#### RNA sequencing

RNA-sequencing data is available using GEO accession numbers GSE226972 and GSE226974.

##### Affinity purification RNA-seq

Pulldowns of Cbp80-PrA, Hpr1-PrA or no-PrA tagged strains were performed as described up until the step of slide loading. At this point, samples were incubated with magnetic beads (Surebeads protein A) conjugated with anti-Yra1 antibody as the second purification step. For each sample, 20 μL of Surebeads protein A (Bio-Rad, 161–4013) was washed with cold mTBT16 buffer supplemented with 0.5% BSA three times and the anti-Yra1 antibody was attached by adding 1 μL of antibody to 40 μL of washed beads followed by incubation overnight with rotation at 8°C. The conjugated beads were washed by mTBT16/0.5% BSA three times and HRV3C cleaved material (25 μL) mixed with the Yra1 antibody conjugated beads adjusting the volume to 125 μL by adding the mTBT16 buffer. The Yra1 pulldown was performed at 8°C with rotation for 10 min. The beads were washed by mTBT16 buffer without protease inhibitor three times and then resuspended in 400 μL of ProK buffer (50 mM Tris-HCl pH 7.8, 50 mM NaCl, 1 mM EDTA, 0.5% SDS). To extract total RNA from the same grindate, 7.5% volume of post-crosslink cell lysate was also taken and mixed with the ProK buffer. To each sample, 100 μg of proteinase K was added and incubated at 50°C for 2 h on the thermal mixer. Crosslinking was then reversed by incubation at 65°C for 1 h with mixing. RNA was extracted with 125:24:1 Phenol: chloroform: iso-amyl alcohol (PCI) pH 4.3–4.7 and ethanol precipitation with overnight −80°C incubation supplemented with 20 μg of glycogen as a carrier. Contaminating DNA was removed by digestion with Turbo DNase (Invitrogen, AM2238) and the RNA was purified with PCI and ethanol precipitation again. RNA samples were resuspended in molecular grade water and 3′-Tag-seq library preparation was carried out using Lexogen QuantSeq kit. Three biological replicates samples were sequenced with single-end RNA-sequencing at the UC Davis Genome Center on an Aviti and NextSeq sequencer.

From sequencing data, unique molecule identifier (UMI) sequences at the 5′ end were extracted and the following 4 nt fixed sequence and 12 nt random primer sequence were trimmed using UMI-tools. Adapter and poly A tail sequences at 3′ ends were also trimmed using bbduk. The rRNA sequence reads were removed with bbsplit. Mapping of trimmed sequence reads on *S. cerevisiae* genome (GCF_000146045.2_R64) was performed using STAR. After removing PCR duplicates with UMI-tools, the read counts in each gene were determined using htseq-counts with the mode intersection-nonempty. Differentially expression analysis using DESeq2 with Wald test was performed to determine significantly enriched genes in each pulldown. Cbp80-PrA and Hpr1-PrA samples were compared to the no-PrA tagged strain IP data and the genes with log2 fold change (FC) > 1 and adjusted p value <0.05 were assigned as significantly enriched in the pulldowns. For the analysis of mRNA gene features, non-coding RNA genes and mitochondrially encoded genes are removed from the list. Gene length and intron information was extracted from *S. cerevisiae* GTF gene annotation file (GCF_000146045.2_R64). To compare expression levels of significantly enriched genes, TPM values calculated from total RNA sequencing data from the Cbp80-PrA strain were used. For the comparison of synthesis rate and mRNA half-life, published data^93^ were used. For the analysis of the potential to form secondary structure, PARS data^92^ was used. Single nucleotide PARS score was averaged over the entire length of the gene and plotted accordingly.

##### Total RNA sequencing from yeast at different growth temperatures

Total RNA from yeast cells grown at 25, 30°C and 37°C were prepared from cell grindate using an RNA extraction kit (Zymo research, R2014), excluding the kits DNase I treatment process. Purified RNA was treated with Turbo DNase for 30 min at 37°C and cleaned by column purification (Zymo research, R1013). 3′-Tag-seq libraries were prepared as described above and two biological replicates were sequenced at the UC Davis Genome Center using a NextSeq sequencer. Differentially expression analysis was performed using DESeq2 with the cutoff of log2FC > 1 or < −1 and adjusted p value <0.05.

#### Western blotting

For testing the mAID tagged protein depletion in the sample for mRNP-SiMPull, cell lysate was prepared as described above from cell grindate without crosslinking. The same volume of 2× SDS sample buffer was added to the cell lysate, denatured at 95°C for 10 min and run on the SDS-PAGE gel. For testing depletion for smFISH, 2 OD of cells were collected, washed by DI water and resuspended with 100 μL of 2× SDS buffer. The cells were broken and lysed by three cycles of 30 s beads beating and 1 min boiling. Western blotting of 3V5-mAID tagged protein, mAID-Yra1 and GAPDH control was performed using anti-V5 (Invitrogen, R960–25), anti-Yra1 and anti-GAPDH (Thermo Fisher, MA5–15738) antibody.

#### smFISH

Single molecule fluorescence *in-situ* hybridization (smFISH) was performed as described^61^ with Atto647 and Alexa 594 dye conjugated FLAP probes. All probes used in this study are listed in Table S6. Yeast cells which has mAID-YRA1 were grown to OD_600_ ~1.0 in YPD medium at 25°C. The culture was split and mixed with same volume of pre-warmed 25°C or 37°C YPD and 5PheIAA (final 1 mM) or same volume of DMSO (control) was added. For the 25°C culture, the cells were further cultured for 2 h. For the 37°C samples, the culture was first warmed in 37°C water bath for 15 min and then continued the culture in 37°C shaker for totally 2 h ~6.5 OD of cells were fixed with 3% formaldehyde for 20 min at room temperature followed by overnight fixation at 4°C. Cells were washed three times by Buffer B (0.1M potassium phosphate buffer pH6.5, 0.5 mM MgCl_2_, 1.2M sorbitol) with centrifugation at 16200 rcf, 1 min, room temperature. Cells were resuspended in digestion mix (425 μL of Buffer B, 40 μL of 200 mM Vanadyl Ribonucleoside Complex (VRC) and 6 μL of 20 mg/mL Zymolyase 20T per sample) and incubated for >1 h until cell walls were digested. After washing the cells by Buffer B with centrifugation at 0.4 rcf, 3 min, room temperature, cells were resuspended by 70% ethanol and rotate at room temperature for 4 h. Totally 40 pmol of gene specific probes and 50 pmol of fluorescent-dye-conjugated FRAP probes were mixed with NEB buffer 3 and hybridized in the thermal cycler with the cycle of 85°C 3 min, 65°C 3 min and 25°C 5min. Permeabilized cells were resuspended by formamide wash buffer (15% formamide, 1x SSC) and incubated for 20 min at room temperature. Hybridization is performed in the hybridization buffer (1x SSC, 0.34 mg/mL E.coli tRNA, 20% formamide, 50 nM hybridized FLAP probes, 0.2 mg/mL BSA, 4 mM VRC and 10.6% Dextran sulfate) on the roller drum at 37°C overnight. After hybridization, cells were washed by formamide wash buffer twice with incubation at 37°C for 30 min. After washing, cells were resuspended in the resuspension buffer (10 mM Tris pH8.0, 1× SSC) and dropped on a glass coverslip (No. 1.5H thickness, Merienfeld cat #0107032). After washing the coverslip by 50 μL of 1× PBS three times, mounting media with DAPI (50% glycerol, 1% DABCO, 1× PBS, 1.5 μg/mL DAPI) supplemented with Catalase, Glucose oxidase, Glucose and Trolox as described above, was dropped and the coverslip was sealed to the glass slide.

#### STED imaging, deconvolution, and data analysis

Imaging of smFISH sample was performed using Leica TCS SP8 STED 3X microscope with 100 × 1.4 NA oil objective. 1504×1504 pixel images with zoom factor 3.1 (resulting pixel size 24.95 × 24.95 nm) were acquired with speed 400, bidirectional X on, Line average 4, Line accu 1, Frame average 1 and Frame accu 1. In each image, 21 z stacks are acquired with the step size 0.2 μm. DAPI and Atto647 dye were simultaneously imaged and frame sequential imaging of two channels (DAPI-Atto647 and Alexa 594) were performed. For DAPI imaging, confocal imaging using 405 nm excitation laser with 40% laser power and PTM detector with the spectrum window 410–499 nm was performed. For Atto647 STED imaging, 645 nm excitation laser with 40% laser power, 775 nm STED laser with 45% laser power, and HyD detector with the spectrum window 652–730 nm and gating 1.2–6 ns were used. For Alexa 594 STED imaging, 598 nm excitation laser with 40% laser power, 775 nm STED laser with 80% laser power, and HyD detector with the spectrum window 605–656 nm and gating 1.2–6 ns were used. The images were deconvolved using Huygens Professional (Scientific Volume imaging) with the parameters, background 5, Max iteration 10 and SNR 10 for DAPI confocal, Saturation factor 7.03, background 0.5, Max iteration 10 and SNR 1.5 for Atto647 STED and Saturation factor 11.41, background 0.5, Max iteration 10 and SNR 1.5 for Alexa 594 STED with other default parameters. The deconvolved 3D images were used for spot detection. After splitting the channel, spot detection and acquisition of the subpixel coordinates were performed using FISH-quant v2.^109^ The spots which overlaps with DAPI signal were manually selected as nuclear mRNP by making mask as described.^110^ In this mask making step, the spots which is aggregated and not clearly separated were eliminated from the analysis. Using the x and y coordinates, spot distance were was measured as described.^110^ In this measurement, only the spots that have distances <300 nm were considered part of the same mRNA. All experiments were performed in two biological replicates. Data plots in Figures 6 and S10 are of each individual replicate.

### QUANTIFICATION AND STATISTICAL ANALYSIS

Statistical test details including test used, p values and sample size are indicated in the figures, figure legends and/or main text at the relevant location.

#### Correction of reporter activity in photobleaching step analysis

##### Modeling photobleaching counts as a finite mixture model of binominals

RBP stoichiometry estimates in isolated mRNPs will be impacted by reporter activities, which was addressed by modeling photobleaching step count data as a finite mixture model of binomials. In this model it is supposed that there are N objects in a population (all mRNPs in a cell) that come from G sub-populations (# of genes) with $N_{i}$ objects (expression level) in each sub-population i that satisfies:

$$N=\sum_{i=1}^{G}\;N_{i}.$$

It is assumed that each sub-population i have consistent properties (i.e., RBP stoichiometry), call it $m_{i}$. The goal is to estimate $m_{i}$, and thus a sample of objects is taken of size $\tilde{n}$. The $\tilde{n}$ objects are sampled with replacement from the population of N objects where N is unknown. The resulting sample composition of $\tilde{n}$ objects are *unknown* as well. If ${\tilde{n}}_{i}$ denotes the number of objects from the i th subpopulation, then $\tilde{n}=\sum_{i=1}^{G}\;{\tilde{n}}_{i}$. In practice, it is possible for some of the ${\tilde{n}}_{i}$ to be zero if the sample size is too small, such as $\tilde{n}<G$, and/or when any sub-population has a small probability of being drawn (i.e., $\frac{N_{i}}{N}$ small). Therefore, it can be written:

$$\tilde{n}=\sum_{i:{\tilde{n}}_{i}>0}^{G}{\tilde{n}}_{i}=\sum_{j=1}^{\tilde{g}}{\tilde{n}}_{\sigma (j)}$$

where $\tilde{g}$ is the number of non-zero ${\tilde{n}}_{i}$ in the sample, ${\tilde{n}}_{\sigma (1)},\ldots ,{\tilde{n}}_{\sigma (\tilde{g})}$ are the non-zero ${\tilde{n}}_{i}$ and σ(j) denote the indices for the sub-populations that appear in the sample. If properties of the selected objects were known, the following would represent the data $m_{i}$ (sorted for convenience)

$$\left(\underset{{\tilde{n}}_{\sigma (1)}}{\underbrace{m_{\sigma (1)},\ldots ,m_{\sigma (1)}}},\ldots ,\underset{{\tilde{n}}_{\sigma (\tilde{g})}}{\underbrace{m_{\sigma (\tilde{g})},\ldots ,m_{\sigma (\tilde{g})}},}\right)$$

Once $\tilde{n}$ objects are selected (bound to the TIRF slide), data on all $\tilde{n}$ objects are collected. Each object with mσ(j) components (fluorescently tagged RBPs) can fluoresce individually with a probability defined by the fluorophore used (mNG p = 0.74 and SNAPf p = 0.78). It is assumed that the probability of detection is independent within objects and between objects. With this, let ${\tilde{X}}_{i,j}$ denote the random variable of the properties of the object (RBP stoichiometry) from sub-population i and replicate j. Hence, for each subpopulation $i,\;{\tilde{X}}_{i,j}$ follows a binomial distribution with parameters $m_{i}$ and p, the number of components for the that population and the reporter activity, respectively. The probability of detection being independent between objects and within objects translates to the random variables ${\tilde{X}}_{i,j}$ being independent for all populations i and replicates j: If all objects were detected, the data would look like:

$$\left(\underset{{\tilde{n}}_{\sigma \left(1\right)}}{\underset{︸}{{\tilde{X}}_{\sigma \left(1\right),1},\ldots ,{\tilde{X}}_{\sigma \left(1\right),{\tilde{n}}_{\sigma \left(1\right)}}}},...,\underset{{\tilde{n}}_{\sigma \left(\tilde{g}\right)}}{\underset{︸}{{\tilde{X}}_{\sigma (\tilde{g}),1},\ldots ,{\tilde{X}}_{\sigma (\tilde{g}),{\tilde{n}}_{\sigma (\tilde{g})}}}}\right)$$

Since all objects are not observed (i.e., ${\tilde{X}}_{i,j}=0$ is unobservable) the previous set of data is mixed with observable and unobservable random variables. Let n denote the number of non-zero counts. Then $n=\sum_{i=1}^{g}\;n_{i}$ where g is the number of sub-populations that *have at least one detection in the sample* and $n_{i}$ denote the number of observable detections for each sub-population. Let $g\leq \tilde{g}$ since it is possible for some sub-populations to go undetected even though they are present and clearly $n\leq \tilde{n}$ holds for the same reason. If $X_{i,j}$ denotes the random variables for the *observed* detections, then it follows that for each sub-population i the $X_{i,j}$ follow a similar binomial distribution as the ${\tilde{X}}_{i,j}$, with parameters $m_{i}$ and p, but are *truncated* at zero. The data of observed counts would look like (if subpopulation membership was known):

$$\left(\underset{n_{1}}{\underbrace{X_{1,1},\ldots ,X_{1,n_{1}}}},\ldots ,\underset{n_{g}}{\underbrace{X_{g,1},\ldots ,X_{g,n_{g}}}},\right).$$

Since sub-population that lack a label (e.g., RBP with an inactive fluorophore) are not observed, then our observed detections are instead modeled as the bivariate random variables $〚(X{)}_{i},C_{i}$, where $X_{i}$ follows a zero-truncated binomial distribution and the $C_{i}$ is the *unobserved* sub-population label for the observed count. Since $C_{i}$ is the random variable for sub-population membership, it is a discrete random variable with distribution $〚P(C{)}_{i}=j=\pi_{j}$ for each sub-population j and $\sum_{j=1}^{g}\;\pi_{j}=1$. Ideally, the sample size is large enough such that the $\pi_{j}$ satisfy $\pi_{j}\approx \frac{N_{j}}{N}$, the proportion of sub-population j in the overall population (not just the sample). Thus, the marginal distribution of the $X_{i}$, which is the distribution of the observable data, follows a finite mixture model given by

$$P\left(X_{i}=x\right)=\sum_{i=1}^{g}P\left(X_{i}=x|C_{i}=j\right)P\left(C_{i}=j\right)\;\;=\sum_{i=1}^{g}\frac{\left(\begin{array}{l} m_{j}\\ x \end{array}\right)p^{x}{\left(1\;-\;p\right)}^{m_{j}\;-\;x}}{1\;-\;{\left(1\;-\;p\right)}^{m_{j}}}\pi_{j}$$

Where

$$P\left(x;m_{j},p\right)=\left\{\begin{array}{c} \frac{\left(\begin{array}{c} m_{j}\\ x \end{array}\right)p^{x}(1\;-\;p{)}^{m_{j}-x}}{1\;-\;(1\;-\;p{)}^{m_{j}}},x=1,\ldots ,m_{j}\\ 0,\;\text{otherwise} \end{array}\right.$$

is the probability mass function for the zero truncated binomial distribution with number of components $m_{j}$ and reporter activity p.

##### Maximum likelihood estimation of component and sub-population sizes

The previous section established that the observed data $\left(X_{1},\ldots ,X_{n}\right)$ follows a finite mixture model of zero-truncated binomial with unknown component and sub-population sizes i.e., $\left(m_{1},\ldots ,m_{g}\right)$ and $\left(\pi_{1},\ldots ,\pi_{g}\right)$ respectively, for some unknown number of sub-populations present in the sample, g. With the model of the data given, estimates can be obtained for these unknown parameters via Maximum Likelihood estimation, due in part to its optimality properties in many statistical models. The likelihood of the model parameters is given by

$$L\left(m_{1},\ldots ,m_{g},\pi_{1},\ldots ,\pi_{g};X_{1},\ldots ,X_{n}\right)=\prod_{i=1}^{n}\;P\left(X_{i}=x;m_{1},\ldots ,m_{g},\pi_{1},\ldots ,\pi_{g}\right)$$

and the maximum likelihood estimators are given by

$$\left({\hat{m}}_{1},\ldots ,{\hat{m}}_{g},{\hat{\pi }}_{1},\ldots ,{\hat{\pi }}_{g}\right)=\underset{\left(m_{1},\ldots ,m_{g},\pi_{1},\ldots ,\pi_{g}\right)}{argmax}L\left(m_{1},\ldots ,m_{g},\pi_{1},\ldots ,\pi_{g};X_{1},\ldots ,X_{n}\right).$$

The standard approach to maximum likelihood estimation for finite mixtures is the Expectation Maximization (EM) algorithm^116^ since it has convergence guarantees when certain regularity conditions of the statistical model hold. But this approach fails in this case in part by those regularity conditions failing to hold, but also due to the fact a subset of the parameters, the component sizes $\left(m_{1},\ldots ,m_{g}\right)$, determine the support of the distribution. As such, alternative maximization was used to simplify the problem by splitting the maximization into two parts (i) maximizing the likelihood over $m_{1},\ldots ,m_{g}$ with $\pi_{1},\ldots ,\pi_{g}$ fixed and (ii) the same maximization of (i) but vice versa, which results in a tractable problem. Step (i) is solved by a simple grid search for a given value of $\pi_{1},\ldots ,\pi_{g}$ and (ii) is solved by convex optimization with $m_{1},\ldots ,m_{g}$ given from step (i). These two maximization stages are repeated until convergence is reached.

##### Estimating the number of sub-populations through model selection

The maximum likelihood estimation approach of the previous section implicitly relied on the number of sub-populations, g, being fixed but does not provide any insight on its estimation. A naive but intuitive approach would be to carry out a similar maximum likelihood approach on g as well, but that approach leads to failure. Since the models are nested with increasing values of g, that is, a particular model with g components is a special case of a model with g+1 components, the maximum likelihood estimators will ‘overfit’ the data as g increases beyond some threshold. Hence, the estimation of g needs to account for the tradeoff between goodness-of-fit (maximum likelihood) and simplicity (over-fitting or model complexity). The Akaike information criterion (AIC)^117^ was used to solve this issue since it is a well-studied tool for model selection. The AIC value of the model with k sub-populations is given by

$$AIC(k)=2(2k-1)-logL\left({\hat{m}}_{1},\ldots ,{\hat{m}}_{k},{\hat{\pi }}_{1},\ldots ,{\hat{\pi }}_{k}\right)$$

where the first term is twice the number of parameters estimated for the current model (model complexity) while the second term is the value of the negative log likelihood with the maximum likelihood estimates of the fitted model (goodness of fit). The best model, or best estimate of g; is taken as the one that minimizes AIC. Hence, the estimator $\hat{g}={argmin}_{k}AIC(k)$ was used.

#### Two-sample testing of photobleaching steps distributions between conditions

To test for differences between two photobleaching step distributions in different conditions (e.g., wildtype vs. mutant) a two-sample test of the following form was used

$$H_{0}\;:F=G\;vs\;H_{a\;}:F\neq G,$$

where F and G are the finite mixture model distributions from two independent experiments under different conditions. Since both distributions are parametric, that is, F=F(θ) with $\theta =\left(m_{1},\ldots ,m_{g},\pi_{1},\ldots ,\pi_{g}\right)$ and G=G(ψ) with $\psi =\left({\underline{m}}_{1},\ldots ,{\underline{m}}_{\underline{g}},{\underline{\pi }}_{1},\ldots ,{\underline{\pi }}_{\underline{g}}\right)$, the previous null and alternative hypotheses are equivalent to

$$H_{0}\;:\theta =\psi \;vs\;H_{a}\;:\theta \neq \psi .$$

A statistical hypothesis test for this situation can reject the null hypothesis based on (i) the number of sub-populations are different between the two $g\neq \underline{g}$, or (ii) $g=\underline{g}$ but the values of the parameters are different i.e., $\left.\left(m_{1},\ldots ,m_{g}\right)\neq 〚(\underline{m}{)}_{1},\ldots ,{\underline{m}}_{\underline{g}}\right)$ or $\left(\pi_{1},\ldots ,\pi_{g}\right)\neq \left({\underline{\pi }}_{1},\ldots ,{\underline{\pi }}_{\underline{g}}\right)$. One possibility is a two-stage testing procedure where the first test is

$$H_{0}\;:g=\underline{g\;}vs.{\;H}_{a}\;:g\neq \underline{g},$$

and if the test passes ($H_{0}\;:g=\underline{g}$ is not rejected), then next test is

$$H_{0}\;:\theta =\psi \;\text{vs}\;H_{a}\;:\theta \neq \psi ,$$

under the assumption $g=\underline{g}$. However, there is no off-the-shelf testing procedure available (to our knowledge at least) and many of the popular hypothesis testing approaches run into difficulty e.g., the likelihood ratio test is the most natural but runs into problems with finite mixture models in general. Therefore, a non-parametric approach using the Kolmogorov-Smirnov two sample test was employed. Since the test is non-parametric it applies if some regularity conditions on both distributions are met e.g., assumptions of continuity. Because the usual continuity assumption does not hold, we instead obtain p values using the bootstrap. All the calculations were carried out in R (R Core team, 2022).

## Supplementary Material

1

2

3

## Figures and Tables

**KEY RESOURCES TABLE:** 

REAGENT or RESOURCE	SOURCE	IDENTIFIER

Antibodies		

Anti-rabbit-biotin	Vector Lab	Cat#BA-1000; RRID:AB_2313606
Anti-mouse-biotin	Invitrogen	Cat#31800; RRID:AB_228305
Anti-mNG	Chromotek	Cat#32F6; RRID:AB_2827566
Anti-HA	Sigma	Cat#H3663; RRID:AB_262051
Anti-Yra1	A. K. Kashyap et al.^101^	NA
Anti-V5	Invitrogen	Cat#R960–25; RRID:AB_2556564
Anti-GAPDH	Thermo Fisher	Cat#MA5–15738; RRID:AB_10977387

Chemicals, peptides, and recombinant proteins		

5-Phenyl-1H-indole-3-acetic acid (5PheIAA)	Bio Academia	Cat#30–003
PLL(20)-g[3.5]-PEG(2)/PEG(3.4)-biotin 20%	SuSoS	Cat# PLL(20)-g[3.5]-PEG(2)/PEG(3.4)-biotin 20%
Streptavidin	Prozyme	Cat#SA10
BSA	Sigma	Cat#A4503
Casein	Sigma	Cat#C3400
Pluronic F-127	Sigma	Cat#P2443
κ-casein	Sigma	Cat#C0406
RNase inhibitor murine	NEB	Cat#M0314L
Antiform B	Sigma	Cat#A5757
SNAP-Surface 549	NEB	Cat#S9112S
SNAP-Surface 649	NEB	Cat#S9159S
RNase A	Sigma	Cat#R6513
Dynabeads M270 Epoxy	Invitrogen	Cat#14302D
Rabbit IgG	Sigma	Cat#I5006
HRV3C protease	AG Scientific	Cat#H-1192
Catalase	Sigma	Cat#C-40
Glucose oxidase	Sigma	Cat#G2133
Trolox	Sigma	Cat#238813
TetraSpeck	Invitrogen	Cat#T7279
Surebeads protein A	Bio-Rad	Cat#161–4013
Proteinase K	Invitrogen	Cat#4333793
Turbo DNase	Invitrogen	Cat#AM2238
Vanadyl Ribonucleoside Complex	NEB	Cat#S1402S
Zymolyase 20T	US Biological	Cat#Z1000
E.coli tRNA	Sigma	Cat#10109541001
DABCO (1,4-diazabicyclo-2.2.2 octane)	Fisher	Cat#AC112470250
DAPI	Sigma	Cat#D9542

Critical commercial assays		

Lexogen QuantSeq kit	Lexogen	Cat#015
RNA extraction kit	Zymo research	Cat#R2014
RNA clean and concentrator-5 kit	Zymo research	Cat#R1013

Deposited data		

RNA-seq data	This paper	GEO: GSE226972 and GSE226974
RNA-seq data analysis codes	This paper	https://zenodo.org/record/8336841
Codes for co-localization analysis	This paper	https://zenodo.org/record/8336835
Code for plotting and statistical analysis	This paper	https://zenodo.org/record/8336859
Uncropped western blot images	This paper	https://zenodo.org/record/8336748
PARS data	M. Kertesz et al.^92^	https://genie.weizmann.ac.il/pubs/PARS10/pars10_catalogs.html
RATE-seq data (mRNA synthesis rate and half-life)	B. Neymotin et al.^93^	https://rnajournal.cshlp.org/content/suppl/2014/08/08/rna.045104.114.DC1/TableS5.xls

Experimental models: Cell lines		

Yeast strains, see Table S3	This paper	NA

Oligonucleotides		

Primers for yeast strain construction, see Table S5	This paper	NA
smFISH probes, see Table S6	This paper	NA

Recombinant DNA		

Plasmids for yeast strain construction, see Table S4	This paper	NA

Software and algorithms		

imscroll	L. J. Friedman and J. Gelles.^102^	https://github.com/gelles-brandeis/CoSMoS_analysis
AGATHA	H. Kaur et al.^103^	https://github.com/hoskinslab/AGATHA
UMI-tools (version 1.0.1)	T. Smith et al.^104^	https://github.com/CGATOxford/UMI-tools
BBMap (version 38.79)	B. Bushnell.^105^	https://sourceforge.net/projects/bbmap/
STAR (version 2.7.10a)	A. Dobin et al.^106^	https://github.com/alexdobin/STAR
Htseq (version 2.0.2)	G. H. Putri etal.^107^	https://github.com/htseq/htseq
DESeq2 (version 1.38.2)	M. I. Love et al.^108^	https://bioconductor.org/packages/release/bioc/html/DESeq2.html
Huygens Professional	Scientific Volume imaging	NA
FISH-quant v2	A. Imbert et al.^109^	https://github.com/fish-quant/big-fish
ImageJ plugin for making cell and nuclear mask	S. Adivarahan and D. Zenklusen.^110^	https://github.com/zenklusenlab/ImageJ_plugins
MATLAB scripts for smFISH 5’-3’ distance analysis	S. Adivarahan and D. Zenklusen.^110^	https://github.com/zenklusenlab/MolCell_DistanceCalc

Other		

No. 1.5H Glass coverslips	Merienfeld	Cat#0107032

## Highlights

- Establish mRNP-SiMPull to quantitate RBP composition in single mRNPs
- Identify variable RBP compositions in heterogeneous nuclear mRNPs
- Show THO complex supports generation of multicopy Yra1 mRNPs
- Demonstrate role for Yra1 in nuclear mRNP compaction

## INCLUSION AND DIVERSITY

We support inclusive, diverse, and equitable conduct of research.

## References

[1] Gehring NH, Wahle E, and Fischer U (2017). Deciphering the mRNP Code: RNA-Bound Determinants of Post-Transcriptional Gene Regulation. Trends Biochem. Sci. 42, 369–382. 10.1016/j.tibs.2017.02.004. 28268044

[2] Licatalosi DD, and Darnell RB (2010). RNA processing and its regulation: global insights into biological networks. Nat. Rev. Genet. 11, 75–87. 10.1038/nrg2673. 20019688 PMC3229837

[3] Mitchell SF, and Parker R (2014). Principles and properties of eukaryotic mRNPs. Mol Cell 54, 547–558. 10.1016/j.molcel.2014.04.033. 24856220

[4] Singh G, Pratt G, Yeo GW, and Moore MJ (2015). The Clothes Make the mRNA: Past and Present Trends in mRNP Fashion. Annu. Rev. Biochem. 84, 325–354. 10.1146/annurev-biochem-080111-092106. 25784054 PMC4804868

[5] Wende W, Friedhoff P, and Sträßer K (2019). Mechanism and Regulation of Co-transcriptional mRNP Assembly and Nuclear mRNA Export. Adv. Exp. Med. Biol. 1203, 1–31. 10.1007/978-3-030-31434-7_1. 31811629

[6] Hentze MW, Castello A, Schwarzl T, and Preiss T (2018). A brave new world of RNA-binding proteins. Nat. Rev. Mol. Cell Biol. 19, 327–341. 10.1038/nrm.2017.130. 29339797

[7] Mitchell SF, Jain S, She M, and Parker R (2013). Global analysis of yeast mRNPs. Nat. Struct. Mol. Biol. 20, 127–133. 10.1038/nsmb.2468. 23222640 PMC3537908

[8] Beckmann BM, Horos R, Fischer B, Castello A, Eichelbaum K, Alleaume A-M, Schwarzl T, Curk T, Foehr S, Huber W, (2015). The RNA-binding proteomes from yeast to man harbour conserved enigmRBPs. Nat. Commun. 6, 10127. 10.1038/ncomms10127. 26632259 PMC4686815

[9] Castello A, Fischer B, Eichelbaum K, Horos R, Beckmann BM, Strein C, Davey NE, Humphreys DT, Preiss T, Steinmetz LM, (2012). Insights into RNA biology from an atlas of mammalian mRNA-binding proteins. Cell 149, 1393–1406. 10.1016/j.cell.2012.04.031. 22658674

[10] Baltz AG, Munschauer M, Schwanhäusser B, Vasile A, Murakawa Y, Schueler M, Youngs N, Penfold-Brown D, Drew K, Milek M, (2012). The mRNA-bound proteome and its global occupancy profile on protein-coding transcripts. Mol Cell 46, 674–690. 10.1016/j.molcel.2012.05.021. 22681889

[11] Castello A, Fischer B, Frese CK, Horos R, Alleaume A-M, Foehr S, Curk T, Krijgsveld J, and Hentze MW (2016). Comprehensive Identification of RNA-Binding Domains in Human Cells. Mol Cell 63, 696–710. 10.1016/j.molcel.2016.06.029. 27453046 PMC5003815

[12] Perez-Perri JI, Rogell B, Schwarzl T, Stein F, Zhou Y, Rettel M, Brosig A, and Hentze MW (2018). Discovery of RNA-binding proteins and characterization of their dynamic responses by enhanced RNA interactome capture. Nat. Commun. 9, 4408. 10.1038/s41467-018-06557-8. 30352994 PMC6199288

[13] Bao X, Guo X, Yin M, Tariq M, Lai Y, Kanwal S, Zhou J, Li N, Lv Y, Pulido-Quetglas C, (2018). Capturing the interactome of newly transcribed RNA. Nat. Methods 15, 213–220. 10.1038/nmeth.4595. 29431736 PMC5967874

[14] Queiroz RML, Smith T, Villanueva E, Marti-Solano M, Monti M, Pizzinga M, Mirea D-M, Ramakrishna M, Harvey RF, Dezi V, (2019). Comprehensive identification of RNA-protein interactions in any organism using orthogonal organic phase separation (OOPS). Nat. Biotechnol. 37, 169–178. 10.1038/s41587-018-0001-2. 30607034 PMC6591131

[15] Trendel J, Schwarzl T, Horos R, Prakash A, Bateman A, Hentze MW, and Krijgsveld J (2019). The Human RNA-Binding Proteome and Its Dynamics during Translational Arrest. Cell 176, 391–403.e19. 10.1016/j.cell.2018.11.004. 30528433

[16] Urdaneta EC, Vieira-Vieira CH, Hick T, Wessels H-H, Figini D, Moschall R, Medenbach J, Ohler U, Granneman S, Selbach M, (2019). Purification of cross-linked RNA-protein complexes by phenol-toluol extraction. Nat. Commun. 10, 990. 10.1038/s41467-019-08942-3. 30824702 PMC6397201

[17] Garcia-Moreno M, Noerenberg M, Ni S, Järvelin AI, González-Almela E, Lenz CE, Bach-Pages M, Cox V, Avolio R, Davis T, (2019). System-wide Profiling of RNA-Binding Proteins Uncovers Key Regulators of Virus Infection. Mol Cell 74, 196–211.e11. 10.1016/j.molcel.2019.01.017. 30799147 PMC6458987

[18] Van Nostrand EL, Freese P, Pratt GA, Wang X, Wei X, Xiao R, Blue SM, Chen J-Y, Cody NAL, Dominguez D, (2020). A large-scale binding and functional map of human RNA-binding proteins. Nature 583, 711–719. 10.1038/s41586-020-2077-3. 32728246 PMC7410833

[19] Tuck AC, and Tollervey D (2013). A transcriptome-wide atlas of RNP composition reveals diverse classes of mRNAs and lncRNAs. Cell 154, 996–1009. 10.1016/j.cell.2013.07.047. 23993093 PMC3778888

[20] Baejen C, Torkler P, Gressel S, Essig K, Söding J, and Cramer P (2014). Transcriptome maps of mRNP biogenesis factors define premRNA recognition. Mol Cell 55, 745–757. 10.1016/j.molcel.2014.08.005. 25192364

[21] Wilkinson ME, Charenton C, and Nagai K (2020). RNA Splicing by the Spliceosome. Annu. Rev. Biochem. 89, 359–388. 10.1146/annurev-biochem-091719-064225. 31794245

[22] Mangus DA, Evans MC, and Jacobson A (2003). Poly(A)-binding proteins: multifunctional scaffolds for the post-transcriptional control of gene expression. Genome Biol. 4, 223. 10.1186/gb-2003-4-7-223. 12844354 PMC193625

[23] Adivarahan S, Livingston N, Nicholson B, Rahman S, Wu B, Rissland OS, and Zenklusen D (2018). Spatial Organization of Single mRNPs at Different Stages of the Gene Expression Pathway. Mol Cell 72, 727–738.e5. 10.1016/j.molcel.2018.10.010. 30415950 PMC6592633

[24] Khong A, and Parker R (2018). mRNP architecture in translating and stress conditions reveals an ordered pathway of mRNP compaction. J. Cell Biol. 217, 4124–4140. 10.1083/jcb.201806183. 30322972 PMC6279387

[25] Ashkenazy-Titelman A, Atrash MK, Boocholez A, Kinor N, and Shav-Tal Y (2022). RNA export through the nuclear pore complex is directional. Nat. Commun. 13, 5881. 10.1038/s41467-022-33572-7. 36202822 PMC9537521

[26] Müller-McNicoll M, and Neugebauer KM (2013). How cells get the message: dynamic assembly and function of mRNA-protein complexes. Nat. Rev. Genet. 14, 275–287. 10.1038/nrg3434. 23478349

[27] Khong A, and Parker R (2020). The landscape of eukaryotic mRNPs. RNA 26, 229–239. 10.1261/rna.073601.119. 31879280 PMC7025503

[28] Skoglund U, Andersson K, Björkroth B, Lamb MM, and Daneholt B (1983). Visualization of the formation and transport of a specific hnRNP particle. Cell 34, 847–855. 10.1016/0092-8674(83)90542-1. 6556087

[29] Wurtz T, Lönnroth A, and Daneholt B (1990). Biochemical characterization of Balbiani ring premessenger RNP particles. Mol. Biol. Rep. 14, 95–96. 10.1007/BF00360431. 2362583

[30] Bonneau F, Basquin J, Steigenberger B, Schäfer T, Schäfer IB, and Conti E (2023). Nuclear mRNPs are compact particles packaged with a network of proteins promoting RNA-RNA interactions. Genes Dev. 37, 505–517. 10.1101/gad.350630.123. 37399331 PMC10393194

[31] Batisse J, Batisse C, Budd A, Böttcher B, and Hurt E (2009). Purification of nuclear poly(A)-binding protein Nab2 reveals association with the yeast transcriptome and a messenger ribonucleoprotein core structure. J. Biol. Chem. 284, 34911–34917. 10.1074/jbc.M109.062034. 19840948 PMC2787353

[32] Singh G, Kucukural A, Cenik C, Leszyk JD, Shaffer SA, Weng Z, and Moore MJ (2012). The Cellular EJC Interactome Reveals Higher-Order mRNP Structure and an EJC-SR Protein Nexus. Cell 151, 915–916. 10.1016/j.cell.2012.10.032. 30360293

[33] Metkar M, Ozadam H, Lajoie BR, Imakaev M, Mirny LA, Dekker J, and Moore MJ (2018). Higher-Order Organization Principles of Pre-translational mRNPs. Mol Cell 72, 715–726.e3. 10.1016/j.molcel.2018.09.012. 30415953 PMC6239896

[34] Tange TØ, Nott A, and Moore MJ (2004). The ever-increasing complexities of the exon junction complex. Curr. Opin. Cell Biol. 16, 279–284. 10.1016/j.ceb.2004.03.012. 15145352

[35] Pacheco-Fiallos B, Vorländer MK, Riabov-Bassat D, Fin L, O’Reilly FJ, Ayala FI, Schellhaas U, Rappsilber J, and Plaschka C (2023). mRNA recognition and packaging by the human transcription-export complex. Nature 616, 828–835. 10.1038/s41586-023-05904-0. 37020021 PMC7614608

[36] Jain A, Liu R, Ramani B, Arauz E, Ishitsuka Y, Ragunathan K, Park J, Chen J, Xiang YK, and Ha T (2011). Probing cellular protein complexes using single-molecule pull-down. Nature 473, 484–488. 10.1038/nature10016. 21614075 PMC3103084

[37] Hoskins AA, Friedman LJ, Gallagher SS, Crawford DJ, Anderson EG, Wombacher R, Ramirez N, Cornish VW, Gelles J, and Moore MJ (2011). Ordered and dynamic assembly of single spliceosomes. Science 331, 1289–1295. 10.1126/science.1198830. 21393538 PMC3086749

[38] Fortes P, Kufel J, Fornerod M, Polycarpou-Schwarz M, Lafontaine D, Tollervey D, and Mattaj IW (1999). Genetic and physical interactions involving the yeast nuclear cap-binding complex. Mol. Cell Biol. 19, 6543–6553. 10.1128/MCB.19.10.6543. 10490594 PMC84624

[39] Green DM, Marfatia KA, Crafton EB, Zhang X, Cheng X, and Corbett AH (2002). Nab2p is required for poly(A) RNA export in Saccharomyces cerevisiae and is regulated by arginine methylation via Hmt1p. J. Biol. Chem. 277, 7752–7760. 10.1074/jbc.M110053200. 11779864

[40] Ulbrich MH, and Isacoff EY (2007). Subunit counting in membrane-bound proteins. Nat. Methods 4, 319–321. 10.1038/nmeth1024. 17369835 PMC2744285

[41] Yu G, Zhao Y, and Li H (2018). The multistructural forms of box C/D ribonucleoprotein particles. RNA 24, 1625–1633. 10.1261/rna.068312.118. 30254138 PMC6239191

[42] Baßler J, and Hurt E (2019). Eukaryotic Ribosome Assembly. Annu. Rev. Biochem. 88, 281–306. 10.1146/annurev-biochem-013118-110817. 30566372

[43] Visa N, Izaurralde E, Ferreira J, Daneholt B, and Mattaj IW (1996). A nuclear cap-binding complex binds Balbiani ring pre-mRNA cotranscriptionally and accompanies the ribonucleoprotein particle during nuclear export. J. Cell Biol. 133, 5–14. 10.1083/jcb.133.1.5. 8601613 PMC2120770

[44] Fortes P, Inada T, Preiss T, Hentze MW, Mattaj IW, and Sachs AB (2000). The yeast nuclear cap binding complex can interact with translation factor eIF4G and mediate translation initiation. Mol Cell 6, 191–196. 10949040

[45] Klama S, Hirsch AG, Schneider UM, Zander G, Seel A, and Krebber H (2022). A guard protein mediated quality control mechanism monitors 5’-capping of pre-mRNAs. Nucleic Acids Res. 50, 11301–11314. 10.1093/nar/gkac952. 36305816 PMC9638935

[46] Estrella LA, Wilkinson MF, and González CI (2009). The shuttling protein Npl3 promotes translation termination accuracy in Saccharomyces cerevisiae. J. Mol. Biol. 394, 410–422. 10.1016/j.jmb.2009.08.067. 19733178 PMC2783964

[47] Holmes RK, Tuck AC, Zhu C, Dunn-Davies HR, Kudla G, Clauder-Munster S, Granneman S, Steinmetz LM, Guthrie C, and Tollervey D (2015). Loss of the Yeast SR Protein Npl3 Alters Gene Expression Due to Transcription Readthrough. PLoS Genet. 11, e1005735. 10.1371/journal.pgen.1005735. 26694144 PMC4687934

[48] Kessler MM, Henry MF, Shen E, Zhao J, Gross S, Silver PA, and Moore CL (1997). Hrp1, a sequence-specific RNA-binding protein that shuttles between the nucleus and the cytoplasm, is required for mRNA 3’-end formation in yeast. Genes Dev. 11, 2545–2556. 10.1101/gad.11.19.2545. 9334319 PMC316558

[49] Gonzá lez CI, Ruiz-Echevarría MJ, Vasudevan S, Henry MF, and Peltz SW (2000). The yeast hnRNP-like protein Hrp1/Nab4 marks a transcript for nonsense-mediated mRNA decay. Mol Cell 5, 489–499. 10.1016/s1097-2765(00)80443-8. 10882134

[50] Derrer CP, Mancini R, Vallotton P, Huet S, Weis K, and Dultz E (2019). The RNA export factor Mex67 functions as a mobile nucleoporin. J. Cell Biol. 218, 3967–3976. 10.1083/jcb.201909028. 31753862 PMC6891080

[51] Ben-Yishay R, Mor A, Shraga A, Ashkenazy-Titelman A, Kinor N, Schwed-Gross A, Jacob A, Kozer N, Kumar P, Garini Y, (2019). Imaging within single NPCs reveals NXF1’s role in mRNA export on the cytoplasmic side of the pore. J. Cell Biol. 218, 2962–2981. 10.1083/jcb.201901127. 31375530 PMC6719458

[52] Ho B, Baryshnikova A, and Brown GW (2018). Unification of Protein Abundance Datasets Yields a Quantitative Saccharomyces cerevisiae Proteome. Cell Syst 6, 192–205.e3. 10.1016/j.cels.2017.12.004. 29361465

[53] Mazza C, Ohno M, Segref A, Mattaj IW, and Cusack S (2001). Crystal structure of the human nuclear cap binding complex. Mol Cell 8, 383–396. 10.1016/s1097-2765(01)00299-4. 11545740

[54] Viphakone N, Voisinet-Hakil F, and Minvielle-Sebastia L (2008). Molecular dissection of mRNA poly(A) tail length control in yeast. Nucleic Acids Res. 36, 2418–2433. 10.1093/nar/gkn080. 18304944 PMC2367721

[55] Tudek A, Krawczyk PS, Mroczek S, Tomecki R, Turtola M, Matylla-Kulinska K, Jensen TH, and Dziembowski A (2021). Global view on the metabolism of RNA poly(A) tails in yeast Saccharomyces cerevisiae. Nat. Commun. 12, 4951. 10.1038/s41467-021-25251-w. 34400637 PMC8367983

[56] Aibara S, Gordon JMB, Riesterer AS, McLaughlin SH, and Stewart M (2017). Structural basis for the dimerization of Nab2 generated by RNA binding provides insight into its contribution to both poly(A) tail length determination and transcript compaction in Saccharomyces cerevisiae. Nucleic Acids Res. 45, 1529–1538. 10.1093/nar/gkw1224. 28180315 PMC5388407

[57] Brune C, Munchel SE, Fischer N, Podtelejnikov AV, and Weis K (2005). Yeast poly(A)-binding protein Pab1 shuttles between the nucleus and the cytoplasm and functions in mRNA export. RNA 11, 517–531. 10.1261/rna.7291205. 15769879 PMC1370741

[58] Turtola M, Manav MC, Kumar A, Tudek A, Mroczek S, Krawczyk PS, Dziembowski A, Schmid M, Passmore LA, Casañal A, (2021). Three-layered control of mRNA poly(A) tail synthesis in Saccharomyces cerevisiae. Genes Dev. 35, 1290–1303. 10.1101/gad.348634.121. 34385261 PMC8415320

[59] Schäfer IB, Yamashita M, Schuller JM, Schüssler S, Reichelt P, Strauss M, and Conti E (2019). Molecular Basis for poly(A) RNP Architecture and Recognition by the Pan2-Pan3 Deadenylase. Cell 177, 1619–1631.e21. 10.1016/j.cell.2019.04.013. 31104843 PMC6547884

[60] Kress TL, Krogan NJ, and Guthrie C (2008). A single SR-like protein, Npl3, promotes pre-mRNA splicing in budding yeast. Mol Cell 32, 727–734. 10.1016/j.molcel.2008.11.013. 19061647 PMC2677966

[61] Sandhu R, Sinha A, and Montpetit B (2021). The SR-protein Npl3 is an essential component of the meiotic splicing regulatory network in Saccharomyces cerevisiae. Nucleic Acids Res. 49, 2552–2568. 10.1093/nar/gkab071. 33577675 PMC7969001

[62] Hackmann A, Wu H, Schneider U-M, Meyer K, Jung K, and Krebber H (2014). Quality control of spliced mRNAs requires the shuttling SR proteins Gbp2 and Hrb1. Nat. Commun. 5, 3123. 10.1038/ncomms4123. 24452287

[63] Yu MC, Bachand F, McBride AE, Komili S, Casolari JM, and Silver PA (2004). Arginine methyltransferase affects interactions and recruitment of mRNA processing and export factors. Genes Dev. 18, 2024–2035. 10.1101/gad.1223204. 15314027 PMC514182

[64] Baierlein C, Hackmann A, Gross T, Henker L, Hinz F, and Krebber H (2013). Monosome formation during translation initiation requires the serine/arginine-rich protein Npl3. Mol. Cell Biol. 33, 4811–4823. 10.1128/MCB.00873-13. 24100011 PMC3889561

[65] Peña A, Gewartowski K, Mroczek S, Cuéllar J, Szykowska A, Prokop A, Czarnocki-Cieciura M, Piwowarski J, Tous C, Aguilera A, (2012). Architecture and nucleic acids recognition mechanism of the THO complex, an mRNP assembly factor. EMBO J. 31, 1605–1616. 10.1038/emboj.2012.10. 22314234 PMC3321177

[66] Schuller SK, Schuller JM, Prabu JR, Baumgärtner M, Bonneau F, Basquin J, and Conti E (2020). Structural insights into the nucleic acid remodeling mechanisms of the yeast THO-Sub2 complex. Elife 9, e61467. 10.7554/eLife.61467. 33191913 PMC7744097

[67] Xie Y, Clarke BP, Kim YJ, Ivey AL, Hill PS, Shi Y, and Ren Y (2021). Cryo-EM structure of the yeast TREX complex and coordination with the SR-like protein Gbp2. Elife 10, e65699. 10.7554/eLife.65699. 33787496 PMC8043747

[68] Strässer K, Masuda S, Mason P, Pfannstiel J, Oppizzi M, Rodriguez-Navarro S, Rondón AG, Aguilera A, Struhl K, Reed R, (2002). TREX is a conserved complex coupling transcription with messenger RNA export. Nature 417, 304–308. 10.1038/nature746. 11979277

[69] Segref A, Sharma K, Doye V, Hellwig A, Huber J, Lührmann R, and Hurt E (1997). Mex67p, a novel factor for nuclear mRNA export, binds to both poly(A)+ RNA and nuclear pores. EMBO J. 16, 3256–3271. 10.1093/emboj/16.11.3256. 9214641 PMC1169942

[70] Santos-Rosa H, Moreno H, Simos G, Segref A, Fahrenkrog B, Panté N, and Hurt E (1998). Nuclear mRNA export requires complex formation between Mex67p and Mtr2p at the nuclear pores. Mol. Cell Biol. 18, 6826–6838. 10.1128/MCB.18.11.6826. 9774696 PMC109266

[71] Strässer K, Bassler J, and Hurt E (2000). Binding of the Mex67p/Mtr2p heterodimer to FXFG, GLFG, and FG repeat nucleoporins is essential for nuclear mRNA export. J. Cell Biol. 150, 695–706. 10.1083/jcb.150.4.695. 10952996 PMC2175290

[72] Strässer K, and Hurt E (2000). Yra1p, a conserved nuclear RNA-binding protein, interacts directly with Mex67p and is required for mRNA export. EMBO J. 19, 410–420. 10.1093/emboj/19.3.410. 10722314 PMC305578

[73] Stutz F, Bachi A, Doerks T, Braun IC, Séraphin B, Wilm M, Bork P, and Izaurralde E (2000). REF, an evolutionary conserved family of hnRNP-like proteins, interacts with TAP/Mex67p and participates in mRNA nuclear export. RNA 6, 638–650. 10.1017/s1355838200000078. 10786854 PMC1369944

[74] Iglesias N, Tutucci E, Gwizdek C, Vinciguerra P, Von Dach E, Corbett AH, Dargemont C, and Stutz F (2010). Ubiquitin-mediated mRNP dynamics and surveillance prior to budding yeast mRNA export. Genes Dev. 24, 1927–1938. 10.1101/gad.583310. 20810649 PMC2932974

[75] Gilbert W, and Guthrie C (2004). The Glc7p nuclear phosphatase promotes mRNA export by facilitating association of Mex67p with mRNA. Mol Cell 13, 201–212. 10.1016/s1097-2765(04)00030-9. 14759366

[76] Gwizdek C, Iglesias N, Rodriguez MS, Ossareh-Nazari B, Hobeika M, Divita G, Stutz F, and Dargemont C (2006). Ubiquitin-associated domain of Mex67 synchronizes recruitment of the mRNA export machinery with transcription. Proc Natl Acad Sci USA 103, 16376–16381. 10.1073/pnas.0607941103. 17056718 PMC1637590

[77] Nair RR, Zabezhinsky D, Gelin-Licht R, Haas BJ, Dyhr MC, Sperber HS, Nusbaum C, and Gerst JE (2021). Multiplexed mRNA assembly into ribonucleoprotein particles plays an operon-like role in the control of yeast cell physiology. Elife 10, e66050. 10.7554/eLife.66050. 33942720 PMC8137142

[78] Ma WK, Cloutier SC, and Tran EJ (2013). The DEAD-box protein Dbp2 functions with the RNA-binding protein Yra1 to promote mRNP assembly. J. Mol. Biol. 425, 3824–3838. 10.1016/j.jmb.2013.05.016. 23721653 PMC3795964

[79] Zenklusen D, Vinciguerra P, Strahm Y, and Stutz F (2001). The yeast hnRNP-Like proteins Yra1p and Yra2p participate in mRNA export through interaction with Mex67p. Mol. Cell Biol. 21, 4219–4232. 10.1128/MCB.21.13.4219-4232.2001. 11390651 PMC87083

[80] Dermody JL, Dreyfuss JM, Villén J, Ogundipe B, Gygi SP, Park PJ, Ponticelli AS, Moore CL, Buratowski S, and Bucheli ME (2008). Unphosphorylated SR-like protein Npl3 stimulates RNA polymerase II elongation. PLoS One 3, e3273. 10.1371/journal.pone.0003273. 18818768 PMC2538588

[81] Schmid M, Olszewski P, Pelechano V, Gupta I, Steinmetz LM, and Jensen TH (2015). The Nuclear PolyA-Binding Protein Nab2p Is Essential for mRNA Production. Cell Rep. 12, 128–139. 10.1016/j.celrep.2015.06.008. 26119729

[82] Chávez S, Beilharz T, Rondón AG, Erdjument-Bromage H, Tempst P, Svejstrup JQ, Lithgow T, and Aguilera A (2000). A protein complex containing Tho2, Hpr1, Mft1 and a novel protein, Thp2, connects transcription elongation with mitotic recombination in Saccharomyces cerevisiae. EMBO J. 19, 5824–5834. 10.1093/emboj/19.21.5824. 11060033 PMC305808

[83] Strässer K, and Hurt E (2001). Splicing factor Sub2p is required for nuclear mRNA export through its interaction with Yra1p. Nature 413, 648–652. 10.1038/35098113. 11675790

[84] Taniguchi I, and Ohno M (2008). ATP-dependent recruitment of export factor Aly/REF onto intronless mRNAs by RNA helicase UAP56. Mol. Cell Biol. 28, 601–608. 10.1128/MCB.01341-07. 17984224 PMC2223434

[85] Ren Y, Schmiege P, and Blobel G (2017). Structural and biochemical analyses of the DEAD-box ATPase Sub2 in association with THO or Yra1. Elife 6, e20070. 10.7554/eLife.20070. 28059701 PMC5218534

[86] Portman DS, O’Connor JP, and Dreyfuss G (1997). YRA1, an essential Saccharomyces cerevisiae gene, encodes a novel nuclear protein with RNA annealing activity. RNA 3, 527–537. 9149233 PMC1369502

[87] Johnson SA, Cubberley G, and Bentley DL (2009). Cotranscriptional recruitment of the mRNA export factor Yra1 by direct interaction with the 3’ end processing factor Pcf11. Mol Cell 33, 215–226. 10.1016/j.molcel.2008.12.007. 19110458 PMC2659397

[88] MacKellar AL, and Greenleaf AL (2011). Cotranscriptional association of mRNA export factor Yra1 with C-terminal domain of RNA polymerase II. J. Biol. Chem. 286, 36385–36395. 10.1074/jbc.M111.268144. 21856751 PMC3196081

[89] Luna R, Rondón AG, Pérez-Calero C, Salas-Armenteros I, and Aguilera A (2019). The THO Complex as a Paradigm for the Prevention of Cotranscriptional R-Loops. Cold Spring Harb Symp Quant Biol 84, 105–114. 10.1101/sqb.2019.84.039594. 32493765

[90] Sanz LA, Hartono SR, Lim YW, Steyaert S, Rajpurkar A, Ginno PA, Xu X, and Chédin F (2016). Prevalent, Dynamic, and Conserved R-Loop Structures Associate with Specific Epigenomic Signatures in Mammals. Mol Cell 63, 167–178. 10.1016/j.molcel.2016.05.032. 27373332 PMC4955522

[91] San Martin-Alonso M, Soler-Oliva ME, García-Rubio M, García-Muse T, and Aguilera A (2021). Harmful R-loops are prevented via different cell cycle-specific mechanisms. Nat. Commun. 12, 4451. 10.1038/s41467-021-24737-x. 34294712 PMC8298424

[92] Kertesz M, Wan Y, Mazor E, Rinn JL, Nutter RC, Chang HY, and Segal E (2010). Genome-wide measurement of RNA secondary structure in yeast. Nature 467, 103–107. 10.1038/nature09322. 20811459 PMC3847670

[93] Neymotin B, Athanasiadou R, and Gresham D (2014). Determination of in vivo RNA kinetics using RATE-seq. RNA 20, 1645–1652. 10.1261/rna.045104.114. 25161313 PMC4174445

[94] Bonnet A, Bretes H, and Palancade B (2015). Nuclear pore components affect distinct stages of intron-containing gene expression. Nucleic Acids Res. 43, 4249–4261. 10.1093/nar/gkv280. 25845599 PMC4417180

[95] Yesbolatova A, Saito Y, Kitamoto N, Makino-Itou H, Ajima R, Nakano R, Nakaoka H, Fukui K, Gamo K, Tominari Y, (2020). The auxin-inducible degron 2 technology provides sharp degradation control in yeast, mammalian cells, and mice. Nat. Commun. 11, 5701. 10.1038/s41467-020-19532-z. 33177522 PMC7659001

[96] Wan Y, Qu K, Ouyang Z, Kertesz M, Li J, Tibshirani R, Makino DL, Nutter RC, Segal E, and Chang HY (2012). Genome-wide measurement of RNA folding energies. Mol Cell 48, 169–181. 10.1016/j.molcel.2012.08.008. 22981864 PMC3483374

[97] Tudek A, Schmid M, Makaras M, Barrass JD, Beggs JD, and Jensen TH (2018). A Nuclear Export Block Triggers the Decay of Newly Synthesized Polyadenylated RNA. Cell Rep. 24, 2457–2467.e7. 10.1016/j.celrep.2018.07.103. 30157437 PMC6130047

[98] Hilleren P, and Parker R (2001). Defects in the mRNA export factors Rat7p, Gle1p, Mex67p, and Rat8p cause hyperadenylation during 3’-end formation of nascent transcripts. RNA 7, 753–764. 10.1017/s1355838201010147. 11350039 PMC1370127

[99] Jensen TH, Patricio K, McCarthy T, and Rosbash M (2001). A blockto mRNA nuclear export in S. cerevisiae leads to hyperadenylation of transcripts that accumulate at the site of transcription. Mol Cell 7, 887–898. 10.1016/s1097-2765(01)00232-5. 11336711

[100] Aguilar L-C, Paul B, Reiter T, Gendron L, Arul Nambi Rajan A, Montpetit R, Trahan C, Pechmann S, Oeffinger M, and Montpetit B (2020). Altered rRNA processing disrupts nuclear RNA homeostasis via competition for the poly(A)-binding protein Nab2. Nucleic Acids Res. 48, 11675–11694. 10.1093/nar/gkaa964. 33137177 PMC7672433

[101] Kashyap AK, Schieltz D, Yates J, and Kellogg DR (2005). Biochemical and genetic characterization of Yra1p in budding yeast. Yeast 22, 43–56. 10.1002/yea.1185. 15584090

[102] Friedman LJ, and Gelles J (2015). Multi-wavelength single-molecule fluorescence analysis of transcription mechanisms. Methods 86, 27–36. 10.1016/j.ymeth.2015.05.026. 26032816 PMC4577447

[103] Kaur H, Jamalidinan F, Condon SGF, Senes A, and Hoskins AA (2019). Analysis of spliceosome dynamics by maximum likelihood fitting of dwell time distributions. Methods 153, 13–21. 10.1016/j.ymeth.2018.11.014. 30472247 PMC6363122

[104] Smith T, Heger A, and Sudbery I (2017). UMI-tools: modeling sequencing errors in Unique Molecular Identifiers to improve quantification accuracy. Genome Res. 27, 491–499. 10.1101/gr.209601.116. 28100584 PMC5340976

[105] Bushnell B (2014). BBMap: A Fast, Accurate, Splice-Aware Aligner (Lawrence Berkeley National Lab. (LBNL)).

[106] Dobin A, Davis CA, Schlesinger F, Drenkow J, Zaleski C, Jha S, Batut P, Chaisson M, and Gingeras TR (2013). STAR: ultrafast universal RNA-seq aligner. Bioinformatics 29, 15–21. 10.1093/bioinformatics/bts635. 23104886 PMC3530905

[107] Putri GH, Anders S, Pyl PT, Pimanda JE, and Zanini F (2022). Analysing high-throughput sequencing data in Python with HTSeq 2.0. Bioinformatics 38, 2943–2945. 10.1093/bioinformatics/btac166. 35561197 PMC9113351

[108] Love MI, Huber W, and Anders S (2014). Moderated estimation of fold change and dispersion for RNA-seq data with DESeq2. Genome Biol. 15, 550. 10.1186/s13059-014-0550-8. 25516281 PMC4302049

[109] Imbert A, Ouyang W, Safieddine A, Coleno E, Zimmer C, Bertrand E, Walter T, and Mueller F (2022). FISH-quant v2: a scalable and modular tool for smFISH image analysis. RNA 28, 786–795. 10.1261/rna.079073.121. 35347070 PMC9074904

[110] Adivarahan S, and Zenklusen D (2021). Probing the Conformational State of mRNPs Using smFISH and SIM. Methods Mol. Biol. 2209, 267–286. 10.1007/978-1-0716-0935-4_17. 33201475

[111] Gietz RD, and Woods RA (2002). Transformation of yeast by lithium acetate/single-stranded carrier DNA/polyethylene glycol method. Methods Enzymol. 350, 87–96. 10.1016/s0076-6879(02)50957-5. 12073338

[112] Longtine MS, McKenzie A, Demarini DJ, Shah NG, Wach A, Brachat A, Philippsen P, and Pringle JR (1998). Additional modules for versatile and economical PCR-based gene deletion and modification in Saccharomyces cerevisiae. Yeast 14, 953–961. 10.1002/(SICI)1097-0061(199807)14:10&lt;953::AID-YEA293&gt;3.0.CO;2-U. 9717241

[113] Winzeler EA, Shoemaker DD, Astromoff A, Liang H, Anderson K, Andre B, Bangham R, Benito R, Boeke JD, Bussey H, (1999). Functional characterization of the S. cerevisiae genome by gene deletion and parallel analysis. Science 285, 901–906. 10.1126/science.285.5429.901. 10436161

[114] Oeffinger M, Wei KE, Rogers R, DeGrasse JA, Chait BT, Aitchison JD, and Rout MP (2007). Comprehensive analysis of diverse ribonucleoprotein complexes. Nat. Methods 4, 951–956. 10.1038/nmeth1101. 17922018

[115] Tan R, Foster PJ, Needleman DJ, and McKenney RJ (2018). Cooperative Accumulation of Dynein-Dynactin at Microtubule Minus-Ends Drives Microtubule Network Reorganization. Dev. Cell 44, 233–247.e4. 10.1016/j.devcel.2017.12.023. 29401420 PMC6082141

[116] Dempster AP, Laird NM, and Rubin DB (1977). Maximum Likelihood from Incomplete Data via the EM Algorithm. J. R. Stat. Soc. Series B Stat. Methodol. 39, 1–38.

[117] Akaike H (1998). Information Theory and an Extension of the Maximum Likelihood Principle. In Selected Papers of Hirotugu Akaike Springer Series in Statistics, Parzen E, Tanabe K, and Kitagawa G, eds. (Springer), pp. 199–213. 10.1007/978-1-4612-1694-0_15.

