# Supplementary material for: Single-molecule quantitation of RNA-binding protein occupancy and stoichiometry defines a role for Yra1 (Aly/REF) in nuclear mRNP organization
Source: Cell Rep. Author manuscript; Available in PMC 2024 Feb 5. (PMC10841842; doi:10.1016/j.celrep.2023.113415)
Supplement: 1 [file NIHMS1948227-supplement-1.pdf]

Cell Reports, Volume *42*

Supplemental information

Single-molecule quantitation of RNA-binding protein occupancy and stoichiometry defines a role for Yra1 (Aly/REF) in nuclear mRNP organization

Ryuta Asada, Andrew Dominguez, and Ben Montpetit

Figure S1

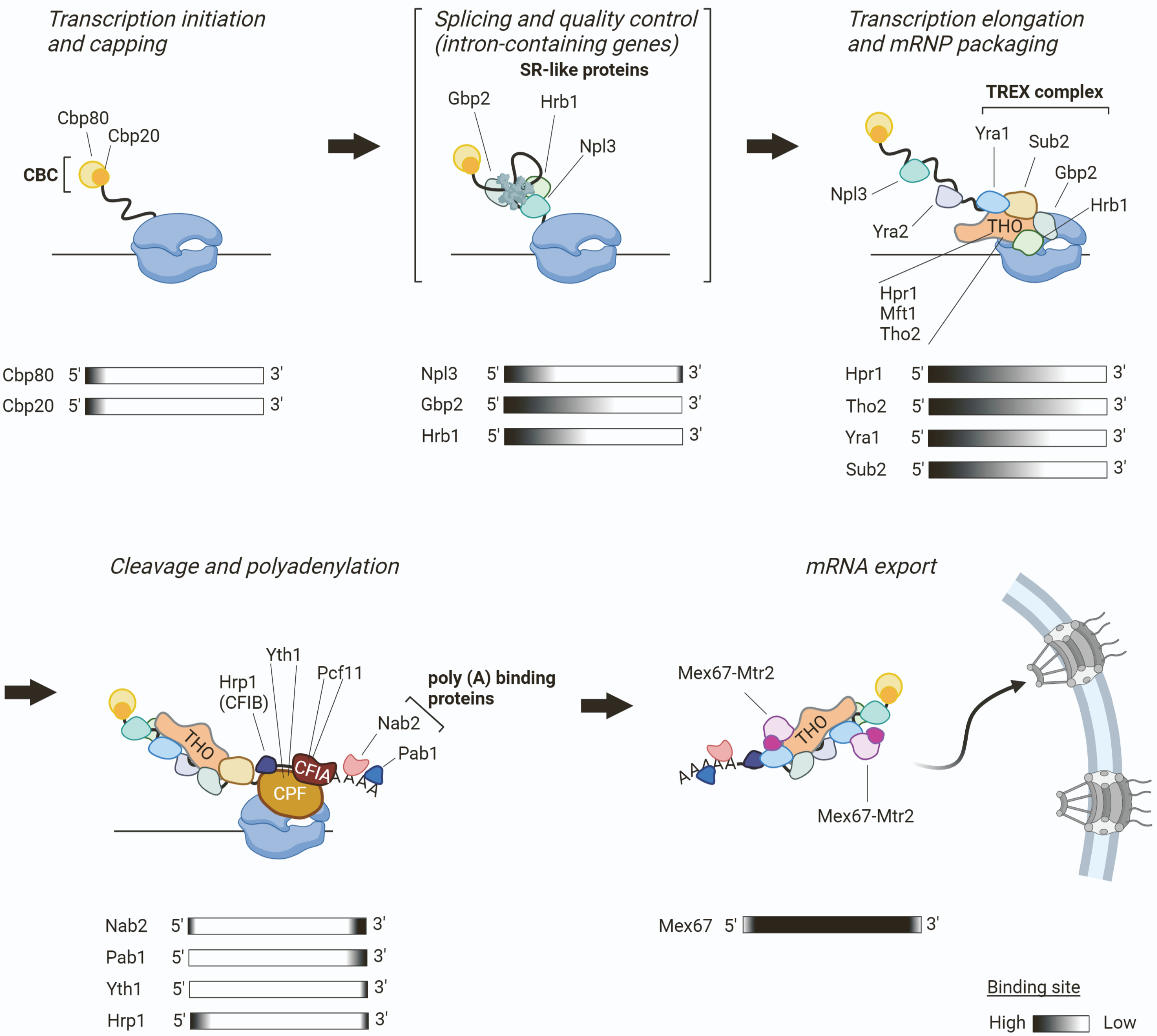

Transcription initiation and capping
Cbp80
Cbp20
CBC
Splicing and quality control (intron-containing genes)
SR-like proteins
Gbp2
Hrb1
Npl3
Transcription elongation and mRNP packaging
TREX complex
Yra1
Sub2
Gbp2
Hrb1
Npl3
Yra2
THO
Hpr1
Mft1
Tho2
Cbp80 5' 3'
Cbp20 5' 3'
Npl3 5' 3'
Gbp2 5' 3'
Hrb1 5' 3'
Hpr1 5' 3'
Tho2 5' 3'
Yra1 5' 3'
Sub2 5' 3'
Cleavage and polyadenylation
Yth1
Hrp1 (CFIB)
Pcf11
poly (A) binding proteins
Nab2
Pab1
THO
CFIA
CPF
mRNA export
Mex67-Mtr2
THO
Mex67-Mtr2
Nab2 5' 3'
Pab1 5' 3'
Yth1 5' 3'
Hrp1 5' 3'
Mex67 5' 3'
Binding site
High
Low

Fig. S1. Currently proposed model for RBP recruitment and mRNP assembly in the mRNA biogenesis and export in yeast, related to introduction. Upon transcription, the nuclear cap binding complex (CBC) composed of a Cbp80 and Cbp20 heterodimer [S1] recognizes the 5′ cap early in transcription to protect the mRNA and further influence downstream events [S2]. The multi-functional SR-like protein, Npl3, promotes splicing, transcription elongation, 3′ end formation, influences recruitment and binding of other RBPs during nuclear mRNP assembly [S3–9]. The other SR-like proteins, Gbp2 and Hrb1, are involved in splicing quality control and facilitate the export of properly spliced mRNAs [S10]. The THO complex (composed of Tho2, Hpr1, Mft1, Thp2 and Tex1) is recruited in response to phosphorylation of the RNA polymerase II (Pol II) c-terminal domain (CTD) to facilitate transcription elongation and recruitment of the DEAD-box ATPase Sub2 and mRNA export adaptor protein Yra1 to form the TRanscription and EXport (TREX) complex [S11–17]. SR-like proteins, Gbp2 and Hrb1, are also recruited to the mRNA via the interaction with TREX complex [S18]. Yra1 has a paralog, Yra2, which can complement the lethality of Yra1 deletion when overexpressed [S19]. Events of 3′ processing are coordinated by the cleavage and polyadenylation factor (CPF) complex, cleavage factor IA (CFIA) complex and cleavage factor IB (CFIB, Hrp1) [S20]. The poly(A)-RNA binding proteins Nab2 and Pab1 are further involved in polyA-tail length control during this reaction [S21]. Of these, TREX components (Hpr1 and Yra1), the SR-like proteins (Npl3, Gbp2 and Hrb1), and Nab2 act as adaptor proteins to recruit the conserved Mex67-Mtr2 (NXF1/TAP-p15 in humans) mRNA export receptor that is responsible for mRNP export through NPCs [S10,16,22–27]. Bars at the bottom of the cartoon represents binding location averaged across the transcriptome as detected by CRAC and PAR-CLIP data [S28,29]. Although model proposed basic factors and their binding sites, it is unclear the actual composition of individual mRNPs and the compositional variation among the genes and different conditions.

Figure S2

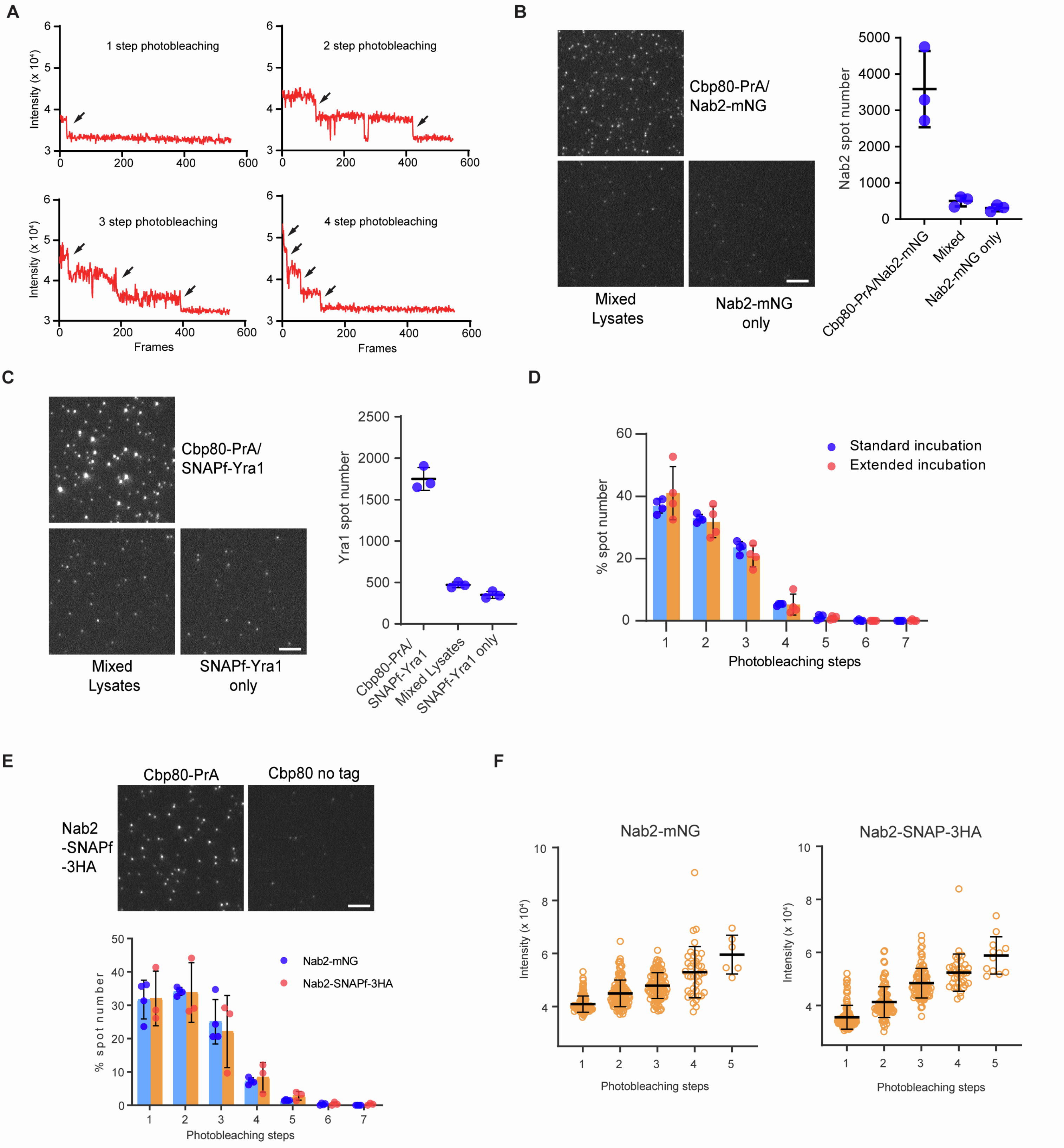
A
1 step photobleaching
2 step photobleaching
3 step photobleaching
4 step photobleaching
Intensity (x 10⁴)
Frames
B
Cbp80-PrA/
Nab2-mNG
Mixed
Lysates
Nab2-mNG
only
Nab2 spot number
Cbp80-PrA/Nab2-mNG
Mixed
Nab2-mNG only
C
Cbp80-PrA/
SNAPf-Yra1
Mixed
Lysates
SNAPf-Yra1
only
Yra1 spot number
Cbp80-PrA/
SNAPf-Yra1
Mixed Lysates
SNAPf-Yra1 only
D
Standard incubation
Extended incubation
% spot number
Photobleaching steps
E
Cbp80-PrA
Cbp80 no tag
Nab2
-SNAPf
-3HA
% spot number
Nab2-mNG
Nab2-SNAPf-3HA
Photobleaching steps
F
Nab2-mNG
Nab2-SNAP-3HA
Intensity (x 10⁴)
Photobleaching steps

Fig. S2. Validation of mRNP-SiMPull as a method to characterize *in vivo* mRNP architectures, related to Figure 1. (**A**) Examples of spot intensity traces during photobleaching step analysis with photobleaching events indicated by black arrows. (**B**) Representative TIRF images of Nab2-mNG obtained by mRNP-SiMPull using input sample from a strain that co-expresses Cbp80-PrA and Nab2-mNG, a Nab2-mNG strain (untagged CBP80), and input made from mixing lysate from two strains individually tagged for Cbp80-PrA and Nab2-mNG. Pulldown was performed by IgG-beads followed by mRNP capturing via mNG antibody on the glass surface. Graph shows the number of detected spots in triplicate experiments with mean and standard deviation (error bar). (**C**) Representative TIRF images of SNAPf-Yra1 obtained by mRNP-SiMPull as described in B. Pulldown was performed by IgG-beads followed by mRNP capturing via Yra1 antibody on the glass surface. Graph shows the number of detected spots in triplicate experiments with mean and standard deviation (error bar). (**D**) Photobleaching step analysis of Nab2-mNG spots detected in a normally processed sample (~50 min from lysis to imaging) versus a sample subject to an extended incubation. Graph displays the percent of spots detected for each photobleaching step in triplicate experiments with mean and standard deviation (error bar). (**E**) Representative TIRF images of Nab2-SNAPf-3HA obtained by mRNP-SiMPull. Pulldown was performed by IgG-beads followed by mRNP capturing via HA antibody on the glass surface. Graph shows photobleaching step analysis of Nab2-mNG (also shown in Fig. 3C) vs. Nab2-SNAPf-3HA in a Cbp80 pulldown. Bars show mean data with standard deviation with dots showing individual data points in triplicate experiments. (**F**) Graphs display spot intensity measurements for Nab2-mNG and Nab2-SNAFf-3HA spots that were determined by photobleaching step analysis to have 1 – 5 molecules present. Mean and standard deviation are shown. Image scale bars, 5 µm.

Figure S3

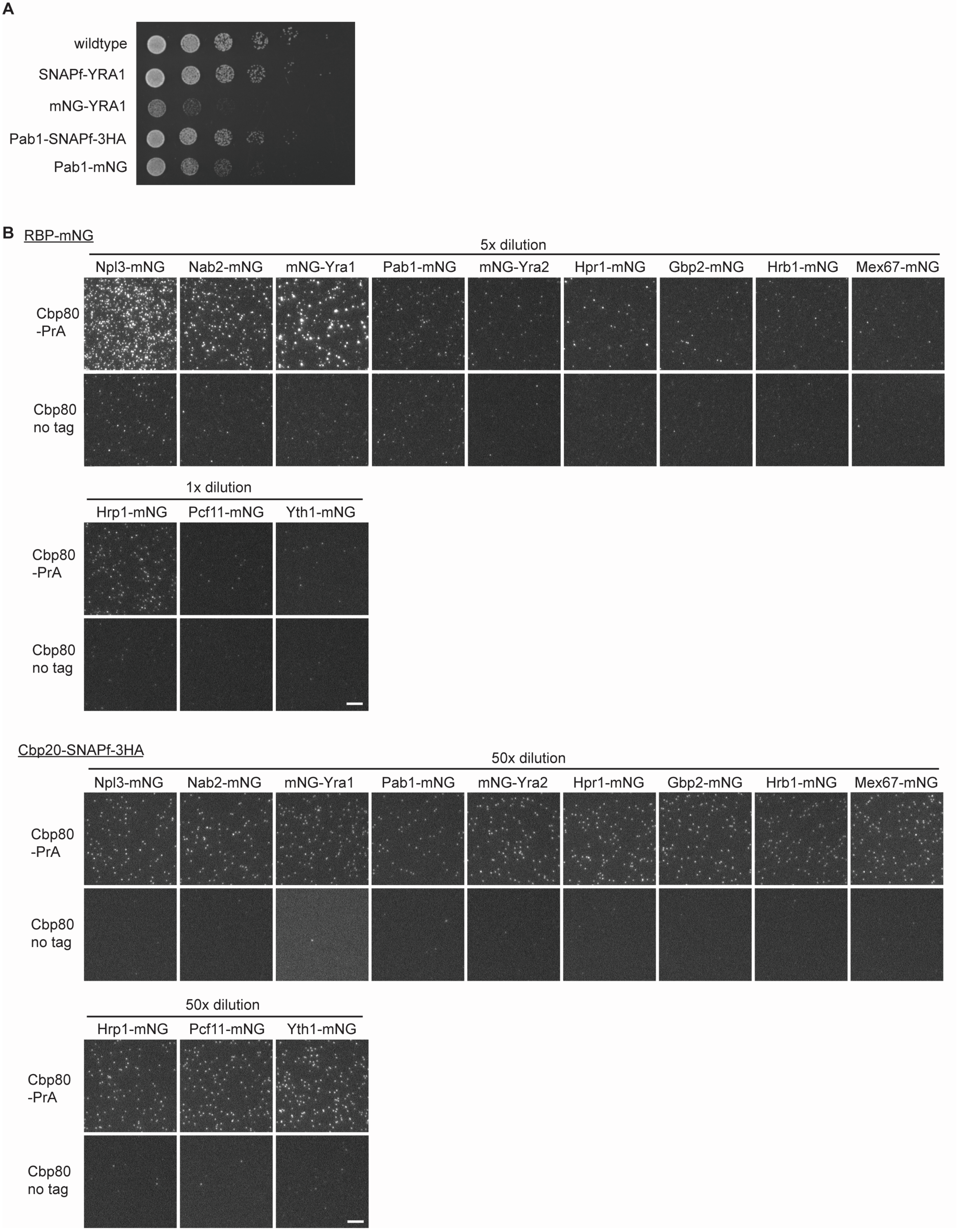
A
wildtype
SNAPf-YRA1
mNG-YRA1
Pab1-SNAPf-3HA
Pab1-mNG
B
RBP-mNG
5x dilution
Npl3-mNG
Nab2-mNG
mNG-Yra1
Pab1-mNG
mNG-Yra2
Hpr1-mNG
Gbp2-mNG
Hrb1-mNG
Mex67-mNG
Cbp80 -PrA
Cbp80 no tag
1x dilution
Hrp1-mNG
Pcf11-mNG
Yth1-mNG
Cbp80 -PrA
Cbp80 no tag
Cbp20-SNAPf-3HA
50x dilution
Npl3-mNG
Nab2-mNG
mNG-Yra1
Pab1-mNG
mNG-Yra2
Hpr1-mNG
Gbp2-mNG
Hrb1-mNG
Mex67-mNG
Cbp80 -PrA
Cbp80 no tag
50x dilution
Hrp1-mNG
Pcf11-mNG
Yth1-mNG
Cbp80 -PrA
Cbp80 no tag

Fig. S3. Occupancy rate analysis of mNG tagged RBPs in Cbp80 pulldown by mRNP-SiMPull, related to Figure 2. (**A**) Yeast spot growth assay showing growth effects caused by fusion of mNG to Yra1 and Pab1 in comparison to SNAP tagging. (**B**) Representative TIRF images used to determine the frequency of target RBP containing mRNPs in the population of total Cbp80 bound mRNPs. mRNP-SiMPull was performed with tagged Cbp80-PrA and untagged Cbp80 strains that have the indicated mNG tagged RBP and SNAPf-3HA tagged Cbp20. Indicated dilution rate of IP-elution was loaded on the glass slide in two independent lanes which were coated with mNG and HA antibody, respectively. Scale bar, 5 µm.

Figure S4

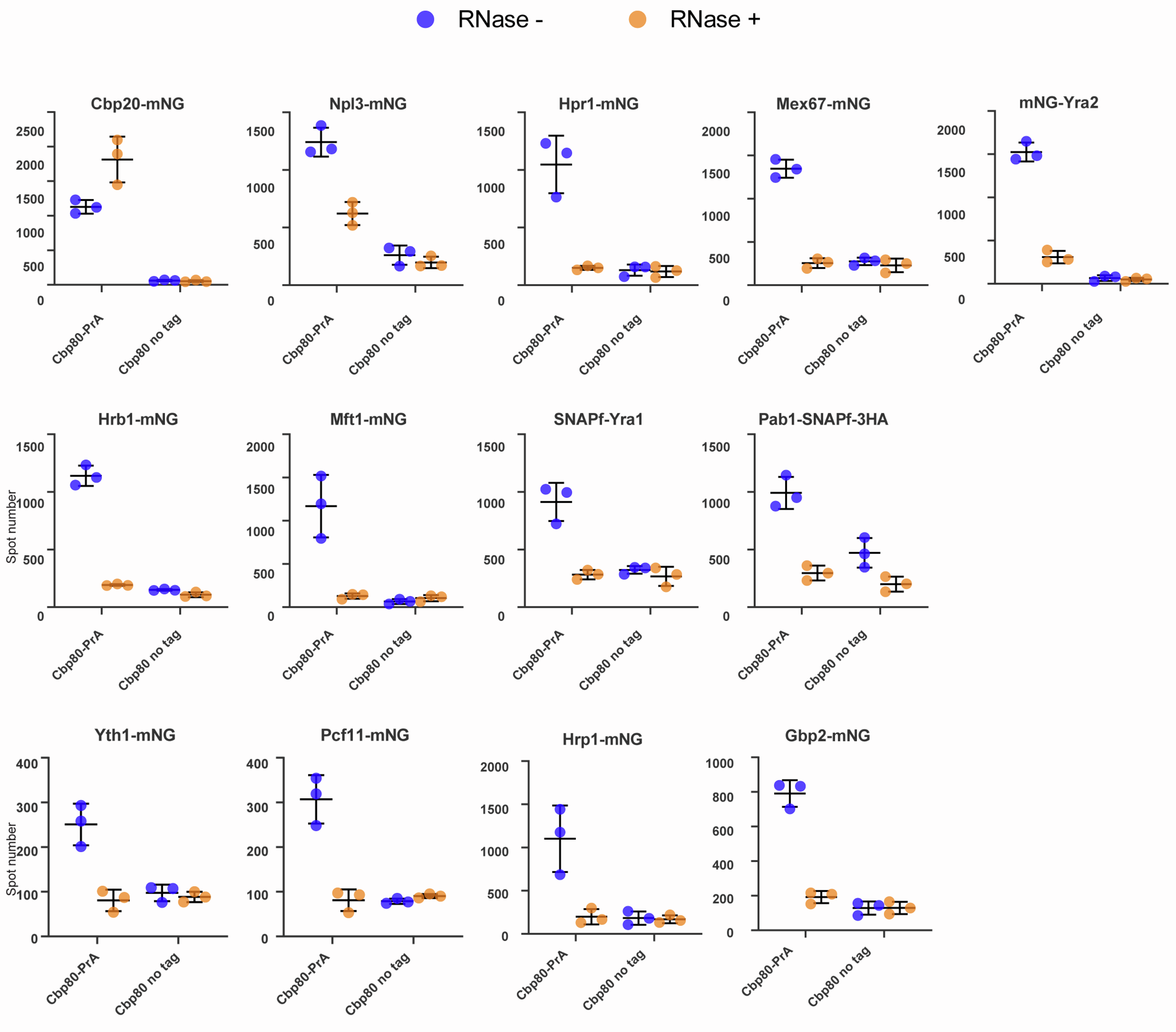
RNase -
RNase +
Cbp20-mNG
Npl3-mNG
Hpr1-mNG
Mex67-mNG
mNG-Yra2
Hrb1-mNG
Mft1-mNG
SNAPf-Yra1
Pab1-SNAPf-3HA
Yth1-mNG
Pcf11-mNG
Hrp1-mNG
Gbp2-mNG
Spot number
Cbp80-PrA
Cbp80 no tag

Fig. S4. RNase sensitivity of mRNP components, related to Figure 3. Graph shows the number of detected spots by mRNP-SiMPull for the indicated RBP with tagged and untagged Cbp80 in triplicate experiments with and without RNase A treatment. Mean and standard deviation (error bar) are indicated by the black bars.

Figure S5

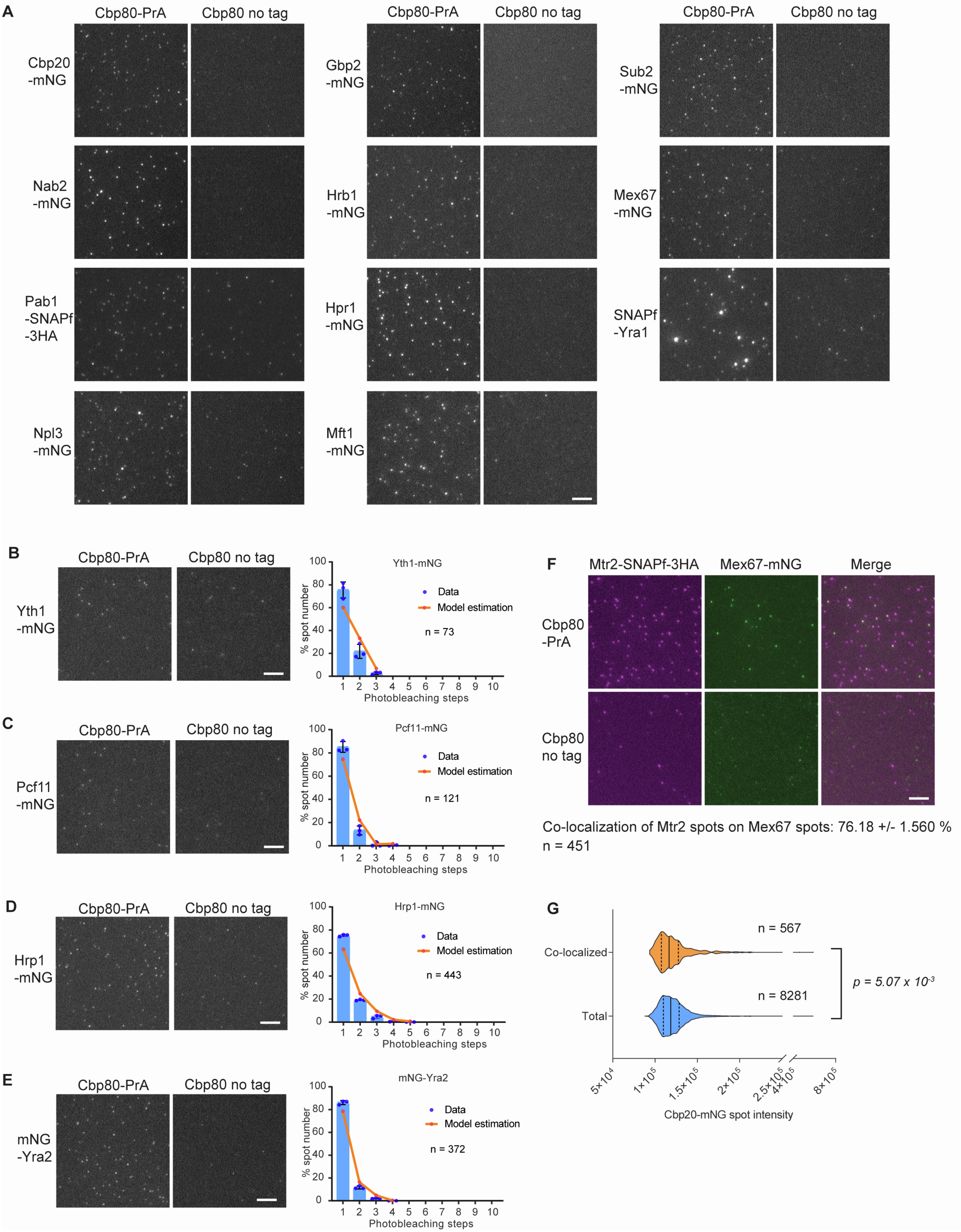
A
Cbp80-PrA
Cbp80 no tag
Cbp20 -mNG
Nab2 -mNG
Pab1 -SNAPf -3HA
Npl3 -mNG
Gbp2 -mNG
Hrb1 -mNG
Hpr1 -mNG
Mft1 -mNG
Sub2 -mNG
Mex67 -mNG
SNAPf -Yra1
B
Yth1 -mNG
Yth1-mNG
Data
Model estimation
n = 73
% spot number
Photobleaching steps
C
Pcf11 -mNG
Pcf11-mNG
n = 121
D
Hrp1 -mNG
Hrp1-mNG
n = 443
E
mNG -Yra2
mNG-Yra2
n = 372
F
Mtr2-SNAPf-3HA
Mex67-mNG
Merge
Cbp80 -PrA
Cbp80 no tag
Co-localization of Mtr2 spots on Mex67 spots: 76.18 +/- 1.560 %
n = 451
G
Co-localized
Total
n = 567
n = 8281
p = 5.07 x 10-3
Cbp20-mNG spot intensity

Fig. S5. Nuclear mRNP composition by photobleaching and co-localization analysis, related to Figure 3. (**A**) Representative TIRF images of target RBPs obtained by mRNP-SiMPull from cell lysates co-expressing Cbp80-PrA (also shown in Figure 3) or an untagged Cbp80. (**B-E**) Stoichiometry analysis of cleavage and polyadenylation factors Yth1, Pcf11 and Hrp1 and Yra1 paralog Yra2 as described in Fig. 3. Average number (n) of spots analyzed per replicate experiment is indicated on each graph. (**F**) Single molecule co-localization analysis between Mex67-mNG and Mtr2-SNAPf-3HA in Cbp80 pulldown. Mean and standard deviation for percent co-localization of Mtr2 spots with all detected Mex67 spots in three replicates are shown. n indicates average number of spots analyzed per replicate experiment. (**G**) Comparison of Cbp20 spot intensities for Yra1 co-localized Cbp20 vs. all Cbp20 spots from imaging data shown in Fig. 4. Pooled triplicate data is shown in the violin plot with solid and dotted lines showing the median and quartile, respectively. n indicates pooled spot number analyzed. The statistically significant (Wilcoxon's rank sum test) reduction in Cbp20-mNG spot intensity for Yra1 co-localized population may reflect fluorescence resonance energy transfer (FRET) between the mNG and SNAP dye. Image scale bars, 5 μm.

Figure S6

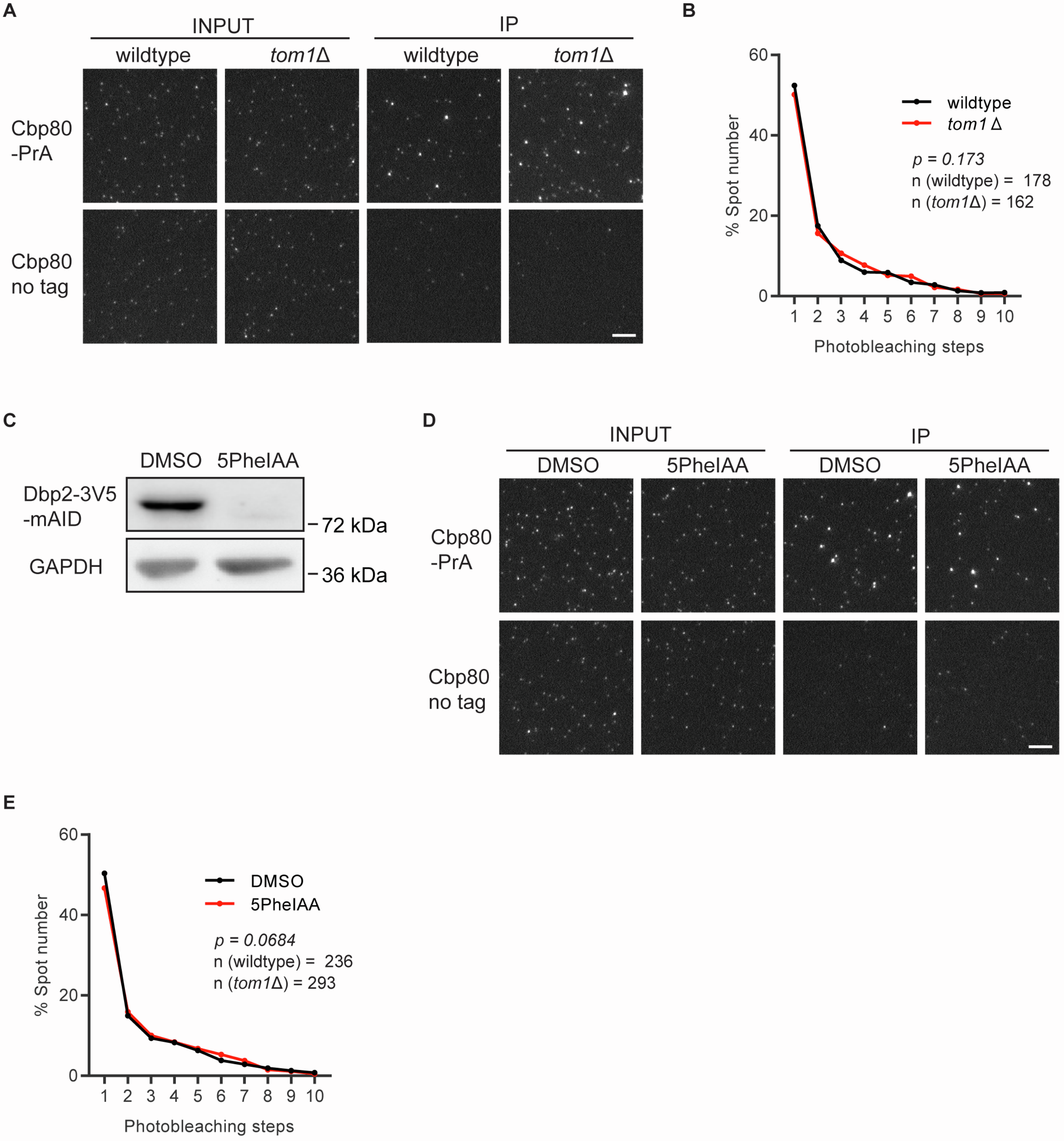
A
INPUT
IP
wildtype
tom1Δ
wildtype
tom1Δ
Cbp80
-PrA
Cbp80
no tag
B
60
40
20
0
% Spot number
1 2 3 4 5 6 7 8 9 10
Photobleaching steps
wildtype
tom1 Δ
p = 0.173
n (wildtype) = 178
n (tom1Δ) = 162
C
DMSO
5PheIAA
Dbp2-3V5
-mAID
GAPDH
72 kDa
36 kDa
D
INPUT
IP
DMSO
5PheIAA
DMSO
5PheIAA
Cbp80
-PrA
Cbp80
no tag
E
60
40
20
0
% Spot number
1 2 3 4 5 6 7 8 9 10
Photobleaching steps
DMSO
5PheIAA
p = 0.0684
n (wildtype) = 236
n (tom1Δ) = 293

Fig. S6. Stoichiometry measurement of SNAPf-Yra1 in *tom1*Δ and Dbp2 depletion strains, related to Figure 3. Representative TIRF images of SNAPf-Yra1 used for stoichiometry analysis in (**A**) wildtype vs. *tom1*Δ and (**D**) control vs. Dbp2 depletion strains from mRNP-SiMPull. Images of INPUT (cell lysate) and IP (PrA pulldown) samples are shown. Line graphs show uncorrected raw mean photobleaching step data from triplicate experiments in (**B**) wildtype vs. *tom1*Δ and (**E**) control vs. Dbp2 depletion strains. p-values were calculated by a non-parametric Kolomogorov-Smirnoff [KS] two-sample test. Average number (n) of spots analyzed per replicate experiment is indicated on each graph. Image scale bars, 5 μm. (**C**) Western blotting with anti-V5 and anti-GAPDH demonstrates depletion of Dbp2-3V5-mAID protein upon addition of 1 μM 5PheIAA for 2 hours as compared to control (DMSO). Protein size marker position is indicated at right side.

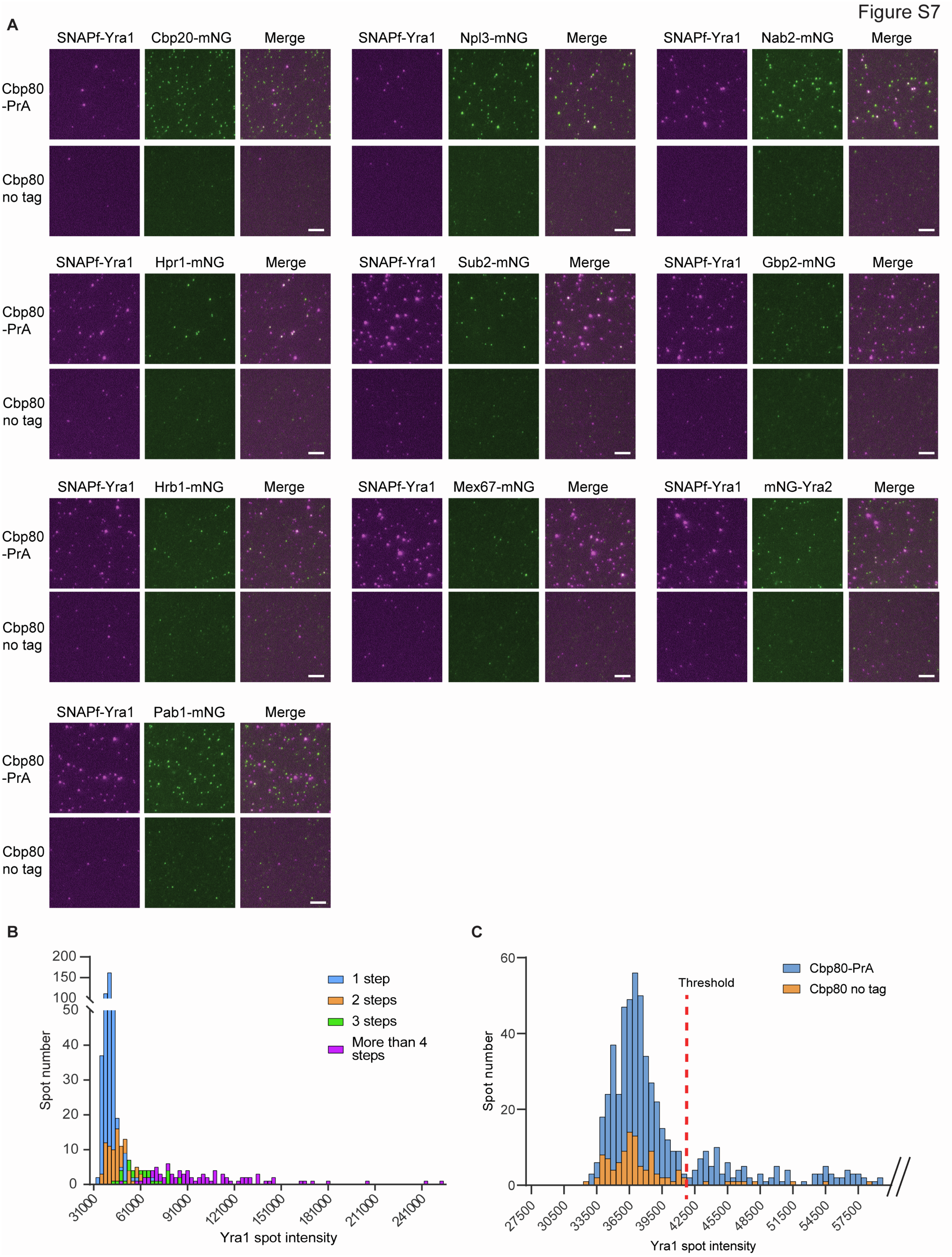
Figure S7
A
SNAPf-Yra1
Cbp20-mNG
Merge
Npl3-mNG
Nab2-mNG
Hpr1-mNG
Sub2-mNG
Gbp2-mNG
Hrb1-mNG
Mex67-mNG
mNG-Yra2
Pab1-mNG
Cbp80 -PrA
Cbp80 no tag
B
1 step
2 steps
3 steps
More than 4 steps
Spot number
Yra1 spot intensity
C
Threshold
Cbp80-PrA
Cbp80 no tag
Spot number
Yra1 spot intensity

Fig. S7. Determination of one vs. multiple Yra1 containing spots for RBP co-localization analysis, related to Figure 4. (**A**) Representative TIRF images used for co-localization analysis between SNAPf-Yra1 and other mNG-tagged RBPs with mRNP-SiMPull showing both Cbp80 pulldown (also shown in Figure 4) and no tag controls. Image scale bars, 5 µm. (**B**) Histogram of Yra1 spot intensity in the first frame of imaging color coded by the stoichiometry determined by photobleaching step analysis. More than 90% of spots in the low intensity peak are one step photobleached molecules. From these distributions, it is possible to separate "mostly one" and "multiple" Yra1 spots based on spot intensity. (**C**) Example of thresholding to separate one and multiple Yra1 spots. Histogram shows spot intensity distribution of total Yra1 spots in Cbp80-PrA and Cbp80 no tag control. Thresholding to separate low intensity peak from higher intensity tail allows for identification of spots with "mostly one" and "multiple" copies of Yra1.

Figure S8

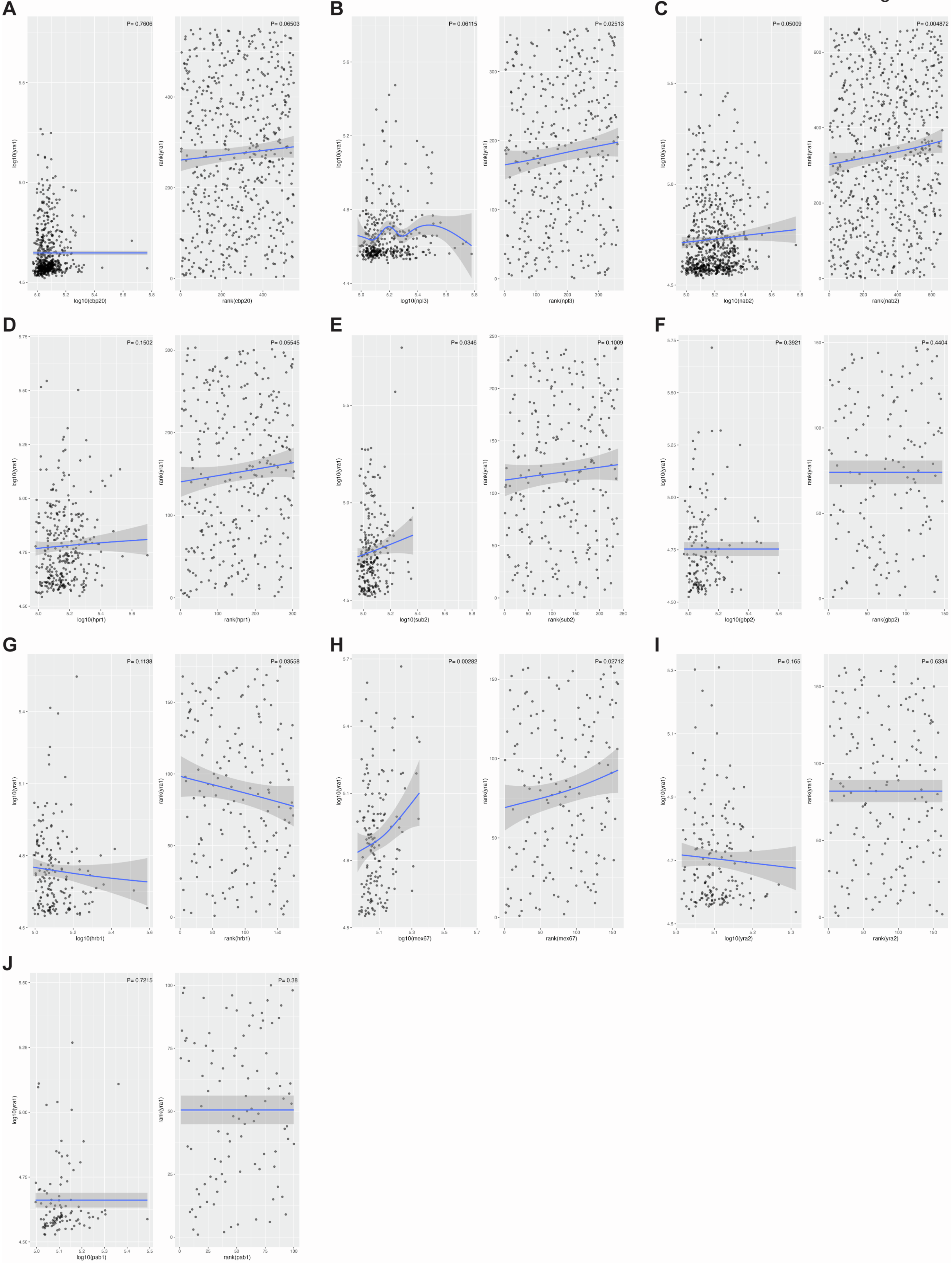

A
P= 0.7606
log10(yra1)
log10(cbp20)
P= 0.06503
rank(yra1)
rank(cbp20)
B
P= 0.06115
log10(yra1)
log10(npl3)
P= 0.02513
rank(yra1)
rank(npl3)
C
P= 0.05009
log10(yra1)
log10(nab2)
P= 0.004872
rank(yra1)
rank(nab2)
D
P= 0.1502
log10(yra1)
log10(hpr1)
P= 0.05545
rank(yra1)
rank(hpr1)
E
P= 0.0346
log10(yra1)
log10(sub2)
P= 0.1009
rank(yra1)
rank(sub2)
F
P= 0.3921
log10(yra1)
log10(gbp2)
P= 0.4404
rank(yra1)
rank(gbp2)
G
P= 0.1138
log10(yra1)
log10(hrb1)
P= 0.03558
rank(yra1)
rank(hrb1)
H
P= 0.00282
log10(yra1)
log10(mex67)
P= 0.02712
rank(yra1)
rank(mex67)
I
P= 0.165
log10(yra1)
log10(yra2)
P= 0.6334
rank(yra1)
rank(yra2)
J
P= 0.7215
log10(yra1)
log10(pab1)
P= 0.38
rank(yra1)
rank(pab1)

Fig. S8. Intensity correlation of co-localized SNAPf-Yra1 and RBP-mNG spots, related to Figure 4. The relationships between Yra1 and other RBPs were investigated within the confines of the data generated by two color mRNP-SiMPull, which relies on the capture and detection of Yra1. Hence, intensity information is known and considered for captured complexes where (i) Yra1 is detected and the other RBP is detected and (ii) Yra1 is detected but the other RBP is not detected. These data would not contain information on complexes of other types (e.g., Yra1 is not detected but the other RBP is detected). This limitation on observable combinations induces statistical dependence between the two measured intensities even if they were independent. Therefore, a generalized additive regression model (GAM) of Yra1 with each RBP was generated to test for a significant regression effect. For completeness, two GAM models were employed: one with a log and the other with a rank transformation applied to the data. Plots shows log (left panel) and rank (right panel) transformed spot intensity of co-localized SNAPf-Yra1 and RBP-mNG (A: Cbp20, B: Npl3, C: Nab2, D: Hpr1, E: Sub2, F: Gbp2, G: Hrb1, H: Mex67, I: Yra2 and J: Pab1) using pooled data from three replicates. The total numbers (n) of spots analyzed for Cbp20, Npl3, Nab2, Hpr1, Sub2, Gbp2, Hrb1, Mex67, Yra2 and Pab1 are 550, 362, 662, 303, 239, 147, 175, 158, 168 and 100, respectively.

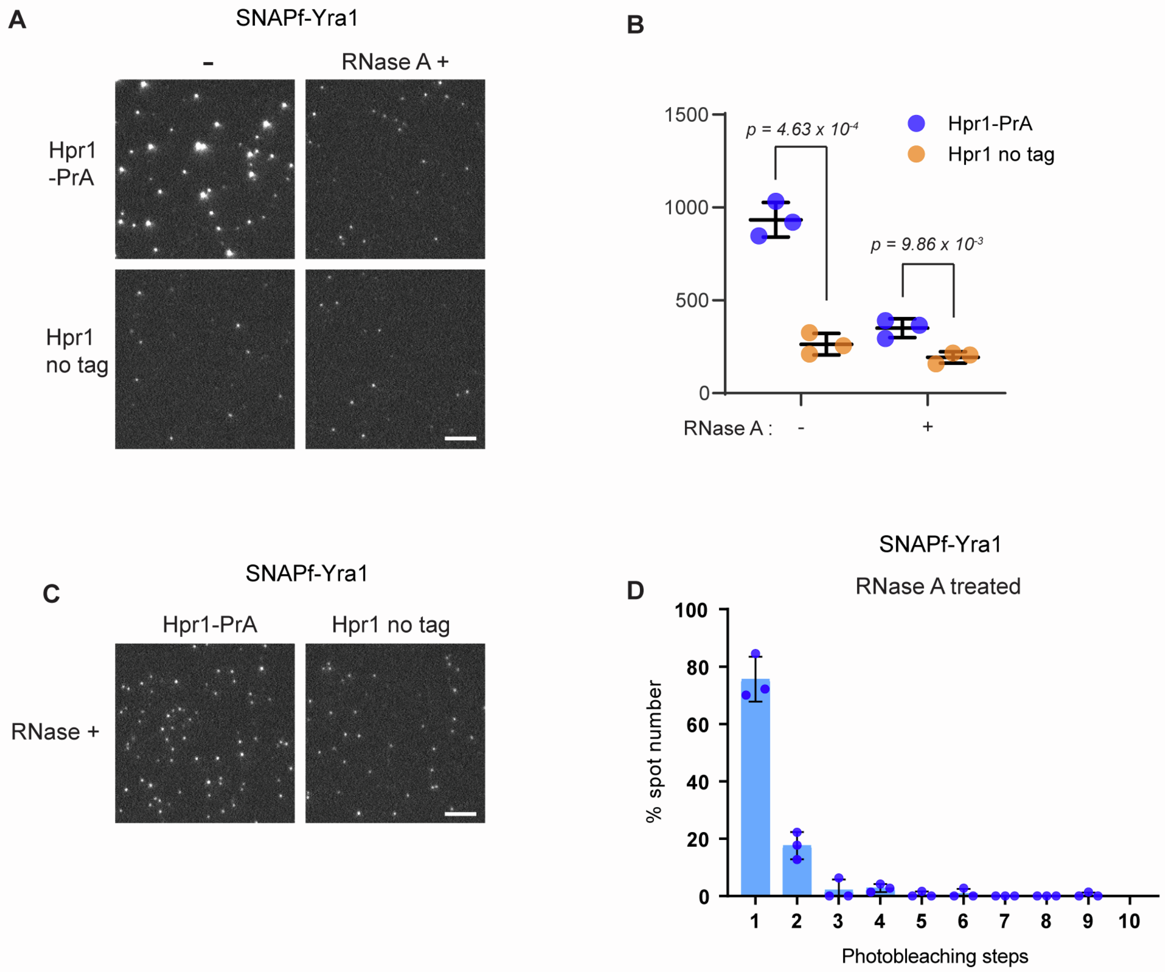
A
SNAPf-Yra1
-
RNase A +
Hpr1
-PrA
Hpr1
no tag
B
1500
1000
500
0
p = 4.63 x 10-4
p = 9.86 x 10-3
Hpr1-PrA
Hpr1 no tag
RNase A :
-
+
C
SNAPf-Yra1
Hpr1-PrA
Hpr1 no tag
RNase +
D
SNAPf-Yra1
RNase A treated
% spot number
100
80
60
40
20
0
1
2
3
4
5
6
7
8
9
10
Photobleaching steps

Fig. S9. Analysis of an RNase resistant Hpr1-Yra1 complex, related to Figure 5. (**A**) Representative TIRF images of SNAPf-Yra1 obtained by mRNP-SiMPull from cell lysates expressing Hpr1-PrA or untagged Hpr1 (no tag) with or without RNase A treatment. (**B**) Graph shows the number of detected spots in triplicate experiments with mean and standard deviation (error bar). p-values are calculated by Student's t-test. (**C**) Representative TIRF images of SNAPf-Yra1 obtained by mRNP-SiMPull in the presence of RNase A. (**D**) Graph shows photobleaching step analysis of RNase resistant SNAPf-Yra1 spots. Bars show mean data with standard deviation with dots showing individual data points in triplicate experiments. Image scale bars, 5 μm.

Figure S10

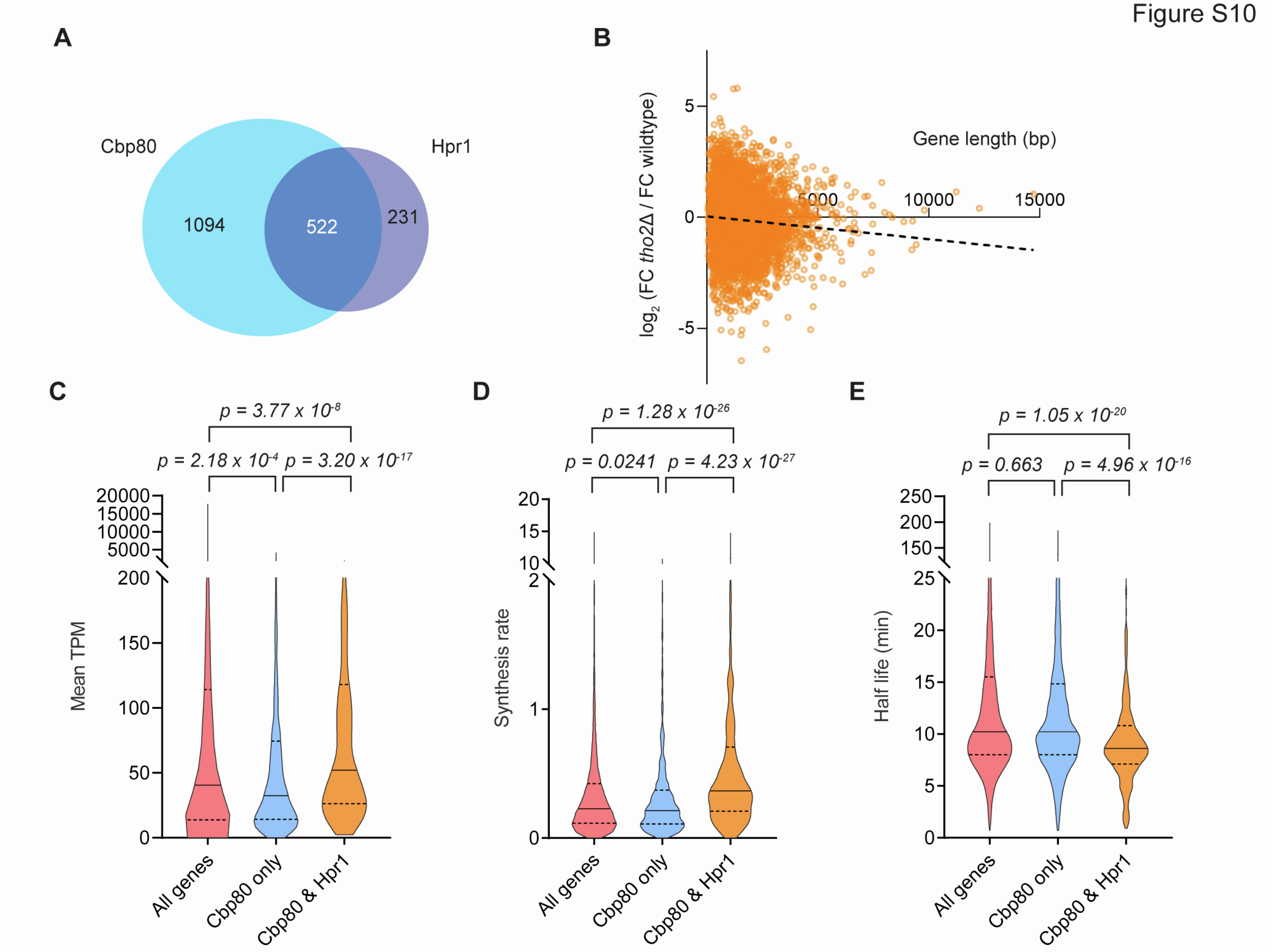

F

	Total genes	Intron containing genes	% Intron containing genes
All genes	6016	271	4.50
Cbp80 only	1094	37	3.38
Cbp80 & Hpr1	522	6	1.15

All vs Cbp80 only: *p-value* = 0.110
All vs Cbp80 & Hpr1: *p-value* = 4.04×10^{-4}
Cbp80 only vs Cbp80 & Hpr1: *p-value* = 0.0146

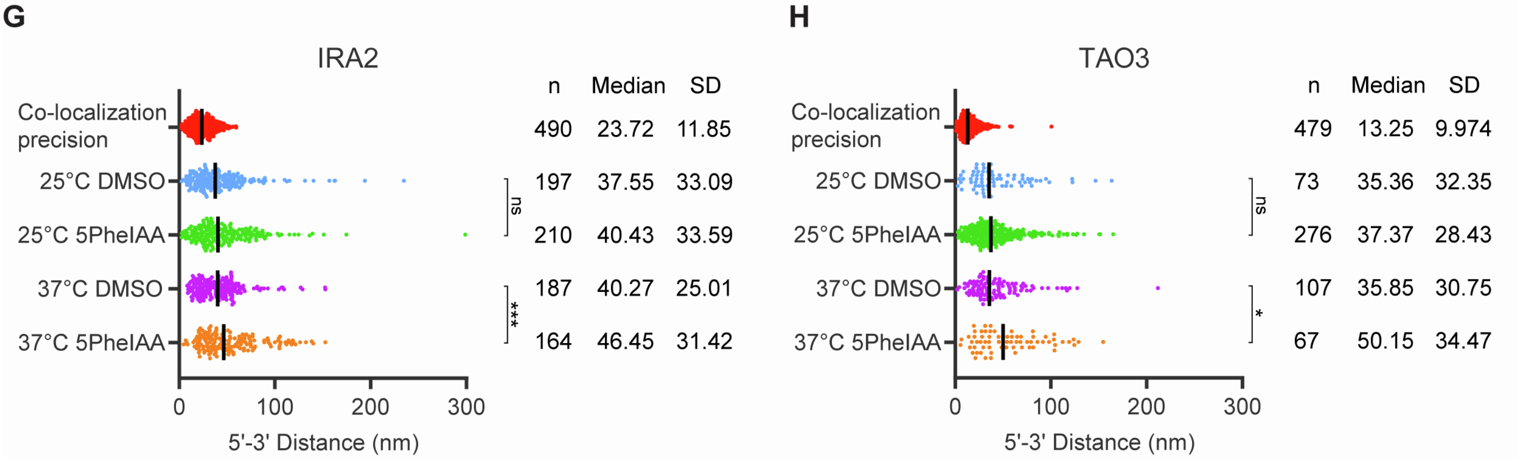

Fig. S10. mRNP compositions with different Yra1 occupancy associated with specific transcripts features and Yra1 function in mRNP compaction, related to Figure 6. (**A**) Venn diagram of gene transcripts determined to be enriched by RNA-seq in Fig. 6 and their overlap between Cbp80/Yra1 and Hpr1/Yra1 two-step pulldowns. (**B**) Scatter plot of gene length and the fold change ratio between *tho2*Δ and wildtype values in Cbp80-PrA/Yra1 pulldowns for each gene. Linear regression line is shown as black dotted line. (**C-E**) Violin plots comparing transcript features among all annotated yeast gene and genes significantly enriched in only Cbp80/Yra1 or in both Cbp80/Yra1 and Hpr1/Yra1 pulldowns, including expression level (TPM value obtained from total RNA sequencing), transcript synthesis rates, and mRNA half-life. Medians and quartiles are shown as solid and dot lines, respectively. p-values were calculated by Wilcoxon's rank sum test. (**F**) Fraction of intron containing genes within all genes in comparison to pulldown enriched populations. p-value was calculated by chi-squared test. (**G, H**) Dot plot of another replicate for distance measurement of IRA2 (G) and TAO3 (H) 5'-3' region as described in Fig 6F and G. Median and SD (standard deviation) are shown in nm. p-values were calculated using Kolmogorov-Smirnov test. ns, not sensitive. * $p < 0.05$. *** $p < 0.001$.

Figure S11

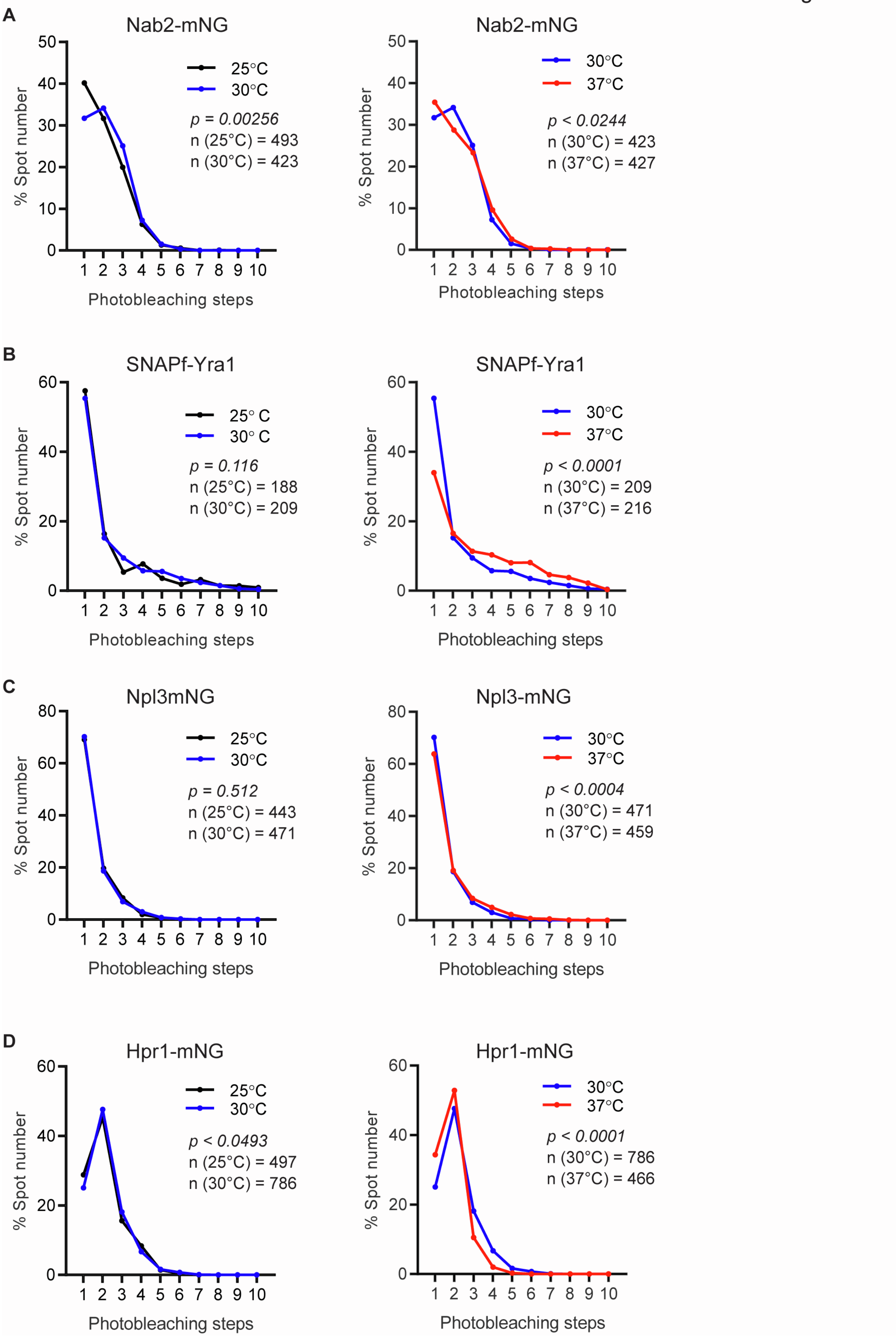

A
Nab2-mNG
25°C
30°C
p = 0.00256
n (25°C) = 493
n (30°C) = 423
% Spot number
Photobleaching steps
Nab2-mNG
30°C
37°C
p < 0.0244
n (30°C) = 423
n (37°C) = 427
B
SNAPf-Yra1
25° C
30° C
p = 0.116
n (25°C) = 188
n (30°C) = 209
SNAPf-Yra1
30°C
37°C
p < 0.0001
n (30°C) = 209
n (37°C) = 216
C
Npl3mNG
25°C
30°C
p = 0.512
n (25°C) = 443
n (30°C) = 471
Npl3-mNG
30°C
37°C
p < 0.0004
n (30°C) = 471
n (37°C) = 459
D
Hpr1-mNG
25°C
30°C
p < 0.0493
n (25°C) = 497
n (30°C) = 786
Hpr1-mNG
30°C
37°C
p < 0.0001
n (30°C) = 786
n (37°C) = 466

Fig. S11. RBP stoichiometry comparison in different cell growth temperature, related to Figure 7. Line graphs showing pairwise comparison of RBPs stoichiometry distribution (**A**: Nab2-mNG, **B**: SNAPf-Yra1, **C**: Npl3-mNG and **D**: Hpr1-mNG) by comparing uncorrected raw mean photobleaching step data from triplicate samples in different growth temperature (25°C vs 30°C and 30°C vs 37°C). p-values were calculated by a non-parametric Kolomogorov-Smirnoff [KS] two-sample tests. Average number (n) of spots analyzed per replicate experiment is indicated on each graph.

Figure S12

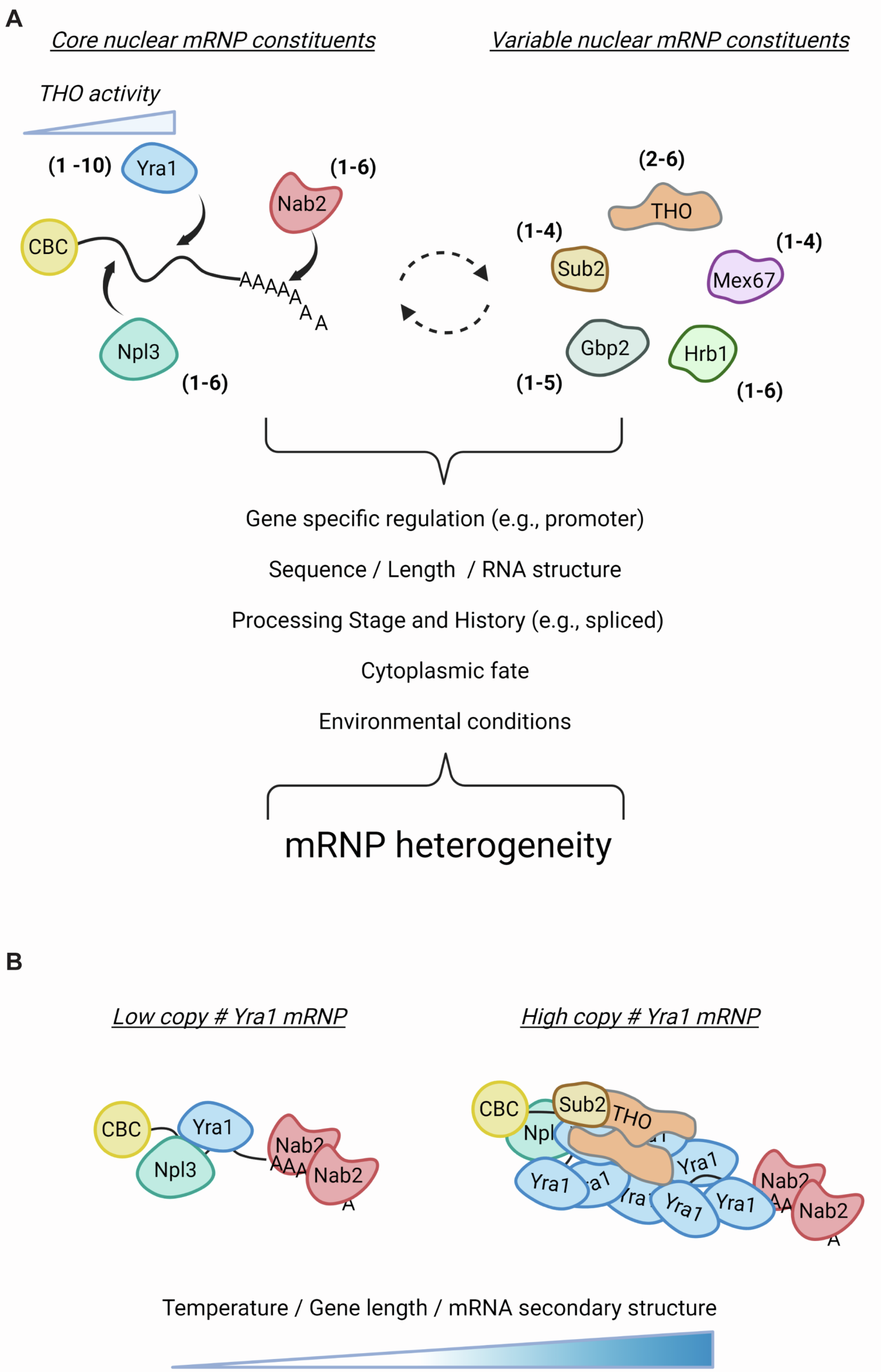
A
Core nuclear mRNP constituents
Variable nuclear mRNP constituents
THO activity
(1 -10)
Yra1
Nab2
(1-6)
CBC
AAAAAAA
Npl3
(1-6)
(2-6)
THO
(1-4)
Sub2
(1-4)
Mex67
Gbp2
Hrb1
(1-5)
(1-6)
Gene specific regulation (e.g., promoter)
Sequence / Length / RNA structure
Processing Stage and History (e.g., spliced)
Cytoplasmic fate
Environmental conditions
mRNP heterogeneity
B
Low copy # Yra1 mRNP
High copy # Yra1 mRNP
CBC
Yra1
Npl3
Nab2
Nab2
Sub2
THO
Temperature / Gene length / mRNA secondary structure

Fig. S12. mRNP heterogeneity as defined by mRNP-SiMPull, related to conclusion. (**A**) CBC capped mRNPs are composed of core components (CBC, Npl3, Nab2 and Yra1) and variable components with both fixed and varied stoichiometries. It is expected that this heterogeneity results from gene-specific regulation (e.g. promoter), transcript features (e.g., length, secondary structure), biogenesis and processing history (e.g., splicing and polyadenylation status), cytoplasmic fate (e.g., translation, transport/localization, storage), and environmental inputs (e.g., temperature). (**B**) Yra1 stoichiometry is altered by co-occupied THO complex function. It is expected that Yra1 stoichiometry is gene and condition dependent, acting within a protein-protein and protein-RNA interaction network to organize and compact mRNPs for nuclear export.

Table S1: Summary of mRNP SiMPull data. Related to Figure 2, 3, S5. Third column "Protein abundance" is from Ho et al 2018 *Cell. Syst* [S30]. The fourth column "Relative frequency of RBP bound mRNPs" is the value of fold enrichment of detected each RBP-mNG spots over the Cbp20-SNAPf-3HA spots in the same sample by mRNP-SiMPull shown in Fig. 2C. The fifth column "RBP stoichiometry on an mRNP (major fraction)" represents stoichiometries observed for an RBP above 1% from the statistical model-corrected photobleaching step data shown in Fig. 3B-L and Fig. S5B-E.

RBP	Description	Abundance (molecule/cell)	Relative fraction of RBP normalized to Cbp20	Stoichiometries observed (>1% of population)
Cbp20	Nuclear cap binding complex	6111	-	1-3 molecule
Nab2	Nuclear poly(A)-RNA binding protein	8716	0.205	1-6 molecule
Pab1	Cytoplasmic poly(A)-RNA binding protein	62422	0.141	1-3 molecule
Npl3	SR-like protein	34936	0.933	1-6 molecule
Gbp2	SR-like protein	3171	0.0233	1-5 molecule
Hrb1	SR-like protein	5191	0.0311	1-6 molecule
Yra1	mRNA export adapter, member TREX complex	26822	0.14	1-10 molecule
Yra2	Homolog of Yra1	3470	0.0423	1-3 molecule
Sub2	DEAD-box RNA helicase, member TREX complex	29329	-	1-4 molecule
Hpr1	THO/TREX complex member	2556	0.0272	2-6 molecule
Mft1	THO/TREX complex member	3969	-	2-6 molecule
Mex67	mRNA export receptor	6514	0.0112	1-4 molecule
Yth1	Cleavage and polyadenylation factor complex (CPF)	1922	0.00186	1-3 molecule
Pcf11	Cleavage factor IA (CFIA)	2800	0.00166	1-4 molecule
Hrp1	Cleavage factor IB (CFIB)	13717	0.0211	1-4 molecule

Table S2: Differentially expressed genes at different growth temperatures. Related to Figure 7 and S11.

25°C vs 30°C	30°C vs 37°C	25°C vs 37°C
None	HSP26, HSP12, HXK1, GSC2, TPO2, BNA2, BAG7, GPH1, STE2, LEU1, BAT1	HSP26, SSE2, CHA1, HXK1, GSC2, TDH1, UBI4, NCW2, GSY2, HOR7, DIA1, CMK2, YOR385W, HTA2, ILS1, RPS11B, RPL21A, KRR1, RPL31A, RPP2B, RPS8B, LEU1, NOP7, YHB1, SBP1, GAR1, THS1, FAR1, RPL15A, RPS0B, CBF5, YEF3, RPL31B, RPS1A, RPS18B, RPL6A, YNL134C, RPP2A, ABP140, SUI3

Table S3: see provided supplemental file.

Table S4: List of plasmids used in this study. Related to STAR Methods.

Plasmid #	Description
pBM836	pFA6a-SNAPf-3HA-hphMX
pBM925	pFA6a-mNG-HIS3MX
pBM1002	pFA6a-PPX-PrA-NatMX
pBM1021	SNAPf-YRA1 integration (URA3 pop out)
pBM1114	mNG-YRA1-HIS3MX integration
pBM1134	mNG-SNU13-HIS3MX integration
pBM1224	pADH1-OsTIR1(F74G)-HIS3 integration
pBM1233	mAID-YRA1-hphMX integration
pBM1250	pFA6a-m3V5-mAID-KanMX
pBM1327	pRS316-pADE3-PrA-PPX-mNG-GST-SNAPf-3HA (URA3/CEN plasmid)
pBM1356	mNG-YRA2-HIS3MX integration

Table S5: List of oligo DNAs used in this study. Related to STAR Methods.

#	Sequence	Description
BMO292	AACCAGTTTTACGCACCAAGAACAAGATACGGAA ATGAACCGGATCCCCGGGTTAATTAA	Forward primer for C-terminal tagging of NAB2
BMO293	TGATGTGTCATGTTCAAAAAGCTTTGAATAGGTGT CTTCCGAATTCGAGCTCGTTTAAAC	Reverse primer for C-terminal tagging of NAB2
BMO150	GAGAACAATTTCGAATTGGATTCAAGAAACAAAG GAAGTTCGGATCCCCGGGTTAATTAA	Forward primer for C-terminal tagging of CBP80
BMO151	AGCGGAGTGATAACGAATGTAGTCCATCCTCCGA ATCTTTGAATTCGAGCTCGTTTAAAC	Reverse primer for C-terminal tagging of CBP80

BMO1799	GAAGGAAAAGAAGTCCAAGAAAGAGAAGAAAGA GAAGAAACGGATCCCCGGGTTAATTAA	Forward primer for C-terminal tagging of NOP58
BMO1800	CGAGGGGTCACTAATTATTAAAATGTAAAATGCA TCCTTAGAATTCGAGCTCGTTTAAAC	Reverse primer for C-terminal tagging of NOP58
BMO1460	TAAAAAGGAAAAGAAGGATAAAAAGAAGAAAAG TAAGGATCGGATCCCCGGGTTAATTAA	Forward primer for C-terminal tagging of NOP56
BMO1461	AAGATGGGATATACTTTATTTCGATTCATTGTTCC TTTTAGAATTCGAGCTCGTTTAAAC	Reverse primer for C-terminal tagging of NOP56
BMO1556	TGGTGGTTGTAGTTTACAGATCTCTTATGCTAGAC GTGATCGGATCCCCGGGTTAATTAA	Forward primer for C-terminal tagging of GBP2
BMO734	CATAAAGTACACAGGTCATGGTTCGGTTGGTGCTT AGGAAGAATTCGAGCTCGTTTAAAC	Reverse primer for C-terminal tagging of GBP2
BMO153	TTTCGATGAAGAAAGAGAAGATGATAACTACGTA CCTCAGCGGATCCCCGGGTTAATTAA	Forward primer for C-terminal tagging of CBP20
BMO154	TCTGTGTGTAGAATCTTTCTCAGATATAAATTGAT TGATTGAATTCGAGCTCGTTTAAAC	Reverse primer for C-terminal tagging of CBP20
BMO1728	TGGGGGTTGTGATTTGGATATATCGTACGCTAAAC GCCTCCGGATCCCCGGGTTAATTAA	Forward primer for C-terminal tagging of HRB1
BMO732	AGATCCAATAGGTGAGAAAGTATATAGATCGAGA GTAGTTGAATTCGAGCTCGTTTAAAC	Reverse primer for C-terminal tagging of HRB1
BMO1617	TTCGAACATTTCTAATGGTTCATCTACCCAAGATA TGAAACGGATCCCCGGGTTAATTAA	Forward primer for C-terminal tagging of HPR1
BMO1618	TTTCTTATCAGTTTAAAATTTCTATTAAGAGGATA ATTTAGAATTCGAGCTCGTTTAAAC	Reverse primer for C-terminal tagging of HPR1
BMO601	CAGAACCAGAGATGCTCCACGTGAAAGATCACCA ACCAGGCGGATCCCCGGGTTAATTAA	Forward primer for C-terminal tagging of NPL3
BMO602	TAATTTCTCCTTTTTTTTTCTCAACTATATAAATGG CTTAGAATTCGAGCTCGTTTAAAC	Reverse primer for C-terminal tagging of NPL3
BMO128	TAGCATGAATGGCATCCCTAGAGAAGCATTTGTG CAGTTCCGGATCCCCGGGTTAATTAA	Forward primer for C-terminal tagging of MEX67
BMO129	AAAGCATGCTGCTGTTCGGAACGTGACGAAAACA GTAGCTGAATTCGAGCTCGTTTAAAC	Reverse primer for C-terminal tagging of MEX67
BMO1099	GTCTTTCAAAAAGGAGCAAGAACAACAAACTGAG CAAGCTCGGATCCCCGGGTTAATTAA	Forward primer for C-terminal tagging of PAB1
BMO1100	GTTTGTTGAGTAGGGAAGTAGGTGATTACATAGA GCATTAGAATTCGAGCTCGTTTAAAC	Reverse primer for C-terminal tagging of PAB1

BMO2106	CAAGGTCGGTTTGGATGACTTAAAGAAATTGGTCACAAAACGGATCCCCGGGTTAATTAA	Forward primer for C-terminal tagging of PCF11
BMO2107	AGTTATTAAATTTAAATGTATATATGCAGTTCTGCTCTTAGAATTCGAGCTCGTTTAAAC	Reverse primer for C-terminal tagging of PCF11
BMO2234	AAAGGAAAGGCGTTTAAACGCAATTATAAACGGTGAAGTTCGGATCCCCGGGTTAATTAA	Forward primer for C-terminal tagging of YTH1
BMO2235	ATGTCTATGAAATCGAAAACCCCCAGCCATGCATAGATCAGAATTCGAGCTCGTTTAAAC	Reverse primer for C-terminal tagging of YTH1
BMO2230	ATACAATAGACGTAATAATGGCTACCATCCATATAATAGGCGGATCCCCGGGTTAATTAA	Forward primer for C-terminal tagging of HRP1
BMO2231	AAAACTTTTCTCTAGTTTTCTACACTTTTCTTTTTTTTTAGAATTCGAGCTCGTTTAAAC	Reverse primer for C-terminal tagging of HRP1
BMO1911	AAGCGATTTTAGTGCGTCTTCCTCTGTTGAAGAAGTAAAACGGATCCCCGGGTTAATTAA	Forward primer for C-terminal tagging of MFT1
BMO1912	CCTTTTCTATTTAGTAAGAGCTATGCATTATACGTGGTCAGAATTCGAGCTCGTTTAAAC	Reverse primer for C-terminal tagging of MFT1
BMO669	CCCAGAAGAAGGCATTGATCCGTCCACTTATTTGAATAATCGGATCCCCGGGTTAATTAA	Forward primer for C-terminal tagging of SUB2
BMO670	TATATAATCTATATAAAAACGTATCTTTTTTCCTTTATTAGAATTCGAGCTCGTTTAAAC	Reverse primer for C-terminal tagging of SUB2
BMO874	GAGAGATGGTGGCTGGGGTAACAGAGGTCGTTCAAACTATCGGATCCCCGGGTTAATTAA	Forward primer for C-terminal tagging of DBP2
BMO875	TTATTAATAGAGATGAATGAATTGAATCACTTTGACTTCAGAATTCGAGCTCGTTTAAAC	Reverse primer for C-terminal tagging of DBP2
BMO1953	GGGCAGTGCATGAAACTG	Forward primer for amplifying tom1Δ::KanMX fragment from the tho2Δ strain in deletion collection
BMO1954	CCAGAGTCTCCTCCACAATC	Reverse primer for amplifying tom1Δ::KanMX fragment from the tho2Δ strain in deletion collection
BMO2088	CCACTGTTCTCGTGTAGTGC	Forward primer for amplifying tho2Δ::KanMX fragment from the tho2Δ strain in deletion collection
BMO1626	GGGTCGTATCGCTAAGAAC	Reverse primer for amplifying tho2Δ::KanMX fragment from the tho2Δ strain in deletion collection

Table S6: see provided supplemental file.

Supplemental References:

[S1]. Fortes, P., Kufel, J., Fornerod, M., Polycarpou-Schwarz, M., Lafontaine, D., Tollervey, D., and Mattaj, I.W. (1999). Genetic and physical interactions involving the yeast nuclear cap-binding complex. Mol Cell Biol *19*, 6543–6553. 10.1128/MCB.19.10.6543.

[S2]. Rambout, X., and Maquat, L.E. (2020). The nuclear cap-binding complex as choreographer of gene transcription and pre-mRNA processing. Genes Dev *34*, 1113–1127. 10.1101/gad.339986.120.

[S3]. Lee, M.S., Henry, M., and Silver, P.A. (1996). A protein that shuttles between the nucleus and the cytoplasm is an important mediator of RNA export. Genes Dev *10*, 1233–1246. 10.1101/gad.10.10.1233.

[S4]. Dermody, J.L., Dreyfuss, J.M., Villén, J., Ogundipe, B., Gygi, S.P., Park, P.J., Ponticelli, A.S., Moore, C.L., Buratowski, S., and Bucheli, M.E. (2008). Unphosphorylated SR-like protein Npl3 stimulates RNA polymerase II elongation. PLoS One *3*, e3273. 10.1371/journal.pone.0003273.

[S5]. Kress, T.L., Krogan, N.J., and Guthrie, C. (2008). A single SR-like protein, Npl3, promotes pre-mRNA splicing in budding yeast. Mol Cell *32*, 727–734. 10.1016/j.molcel.2008.11.013.

[S6]. Sandhu, R., Sinha, A., and Montpetit, B. (2021). The SR-protein Npl3 is an essential component of the meiotic splicing regulatory network in Saccharomyces cerevisiae. Nucleic Acids Res *49*, 2552–2568. 10.1093/nar/gkab071.

[S7]. Bucheli, M.E., He, X., Kaplan, C.D., Moore, C.L., and Buratowski, S. (2007). Polyadenylation site choice in yeast is affected by competition between Npl3 and polyadenylation factor CFI. RNA *13*, 1756–1764. 10.1261/rna.607207.

[S8]. Holmes, R.K., Tuck, A.C., Zhu, C., Dunn-Davies, H.R., Kudla, G., Clauder-Munster, S., Granneman, S., Steinmetz, L.M., Guthrie, C., and Tollervey, D. (2015). Loss of the Yeast SR Protein Npl3 Alters Gene Expression Due to Transcription Readthrough. PLoS Genet *11*, e1005735. 10.1371/journal.pgen.1005735.

[S9]. Keil, P., Wulf, A., Kachariya, N., Reuscher, S., Hühn, K., Silbern, I., Altmüller, J., Keller, M., Stehle, R., Zarnack, K., et al. (2023). Npl3 functions in mRNP assembly by recruitment of mRNP components to the transcription site and their transfer onto the mRNA. Nucleic Acids Res *51*, 831–851. 10.1093/nar/gkac1206.

[S10]. Hackmann, A., Wu, H., Schneider, U.-M., Meyer, K., Jung, K., and Krebber, H. (2014). Quality control of spliced mRNAs requires the shuttling SR proteins Gbp2 and Hrb1. Nat Commun *5*, 3123. 10.1038/ncomms4123.

[S11]. Schuller, S.K., Schuller, J.M., Prabu, J.R., Baumgärtner, M., Bonneau, F., Basquin, J., and Conti, E. (2020). Structural insights into the nucleic acid remodeling mechanisms of the yeast THO-Sub2 complex. Elife *9*, e61467. 10.7554/eLife.61467.

[S12]. Chávez, S., Beilharz, T., Rondón, A.G., Erdjument-Bromage, H., Tempst, P., Svejstrup, J.Q., Lithgow, T., and Aguilera, A. (2000). A protein complex containing Tho2, Hpr1, Mft1 and a novel protein, Thp2, connects transcription elongation with mitotic recombination in Saccharomyces cerevisiae. EMBO J *19*, 5824–5834. 10.1093/emboj/19.21.5824.

[S13]. Meinel, D.M., Burkert-Kautzsch, C., Kieser, A., O'Duibhir, E., Siebert, M., Mayer, A., Cramer, P., Söding, J., Holstege, F.C.P., and Sträßer, K. (2013). Recruitment of TREX to the transcription machinery by its

direct binding to the phospho-CTD of RNA polymerase II. PLoS Genet *9*, e1003914. 10.1371/journal.pgen.1003914.
[S14]. Strässer, K., Masuda, S., Mason, P., Pfannstiel, J., Oppizzi, M., Rodriguez-Navarro, S., Rondón, A.G., Aguilera, A., Struhl, K., Reed, R., et al. (2002). TREX is a conserved complex coupling transcription with messenger RNA export. Nature *417*, 304–308. 10.1038/nature746.
[S15]. Strässer, K., and Hurt, E. (2001). Splicing factor Sub2p is required for nuclear mRNA export through its interaction with Yra1p. Nature *413*, 648–652. 10.1038/35098113.
[S16]. Stutz, F., Bachi, A., Doerks, T., Braun, I.C., Séraphin, B., Wilm, M., Bork, P., and Izaurralde, E. (2000). REF, an evolutionary conserved family of hnRNP-like proteins, interacts with TAP/Mex67p and participates in mRNA nuclear export. RNA *6*, 638–650. 10.1017/s1355838200000078.
[S17]. Zenklusen, D., Vinciguerra, P., Wyss, J.-C., and Stutz, F. (2002). Stable mRNP formation and export require cotranscriptional recruitment of the mRNA export factors Yra1p and Sub2p by Hpr1p. Mol Cell Biol *22*, 8241–8253. 10.1128/MCB.22.23.8241-8253.2002.
[S18]. Hurt, E., Luo, M.-J., Röther, S., Reed, R., and Strässer, K. (2004). Cotranscriptional recruitment of the serine-arginine-rich (SR)-like proteins Gbp2 and Hrb1 to nascent mRNA via the TREX complex. Proc Natl Acad Sci U S A *101*, 1858–1862. 10.1073/pnas.0308663100.
[S19]. Zenklusen, D., Vinciguerra, P., Strahm, Y., and Stutz, F. (2001). The yeast hnRNP-Like proteins Yra1p and Yra2p participate in mRNA export through interaction with Mex67p. Mol Cell Biol *21*, 4219–4232. 10.1128/MCB.21.13.4219-4232.2001.
[S20]. Kumar, A., Clerici, M., Muckenfuss, L.M., Passmore, L.A., and Jinek, M. (2019). Mechanistic insights into mRNA 3'-end processing. Curr Opin Struct Biol *59*, 143–150. 10.1016/j.sbi.2019.08.001.
[S21]. Turtola, M., Manav, M.C., Kumar, A., Tudek, A., Mroczek, S., Krawczyk, P.S., Dziembowski, A., Schmid, M., Passmore, L.A., Casañal, A., et al. (2021). Three-layered control of mRNA poly(A) tail synthesis in Saccharomyces cerevisiae. Genes Dev *35*, 1290–1303. 10.1101/gad.348634.121.
[S22]. Strässer, K., and Hurt, E. (2000). Yra1p, a conserved nuclear RNA-binding protein, interacts directly with Mex67p and is required for mRNA export. EMBO J *19*, 410–420. 10.1093/emboj/19.3.410.
[S23]. Gwizdek, C., Iglesias, N., Rodriguez, M.S., Ossareh-Nazari, B., Hobeika, M., Divita, G., Stutz, F., and Dargemont, C. (2006). Ubiquitin-associated domain of Mex67 synchronizes recruitment of the mRNA export machinery with transcription. Proc Natl Acad Sci U S A *103*, 16376–16381. 10.1073/pnas.0607941103.
[S24]. Iglesias, N., Tutucci, E., Gwizdek, C., Vinciguerra, P., Von Dach, E., Corbett, A.H., Dargemont, C., and Stutz, F. (2010). Ubiquitin-mediated mRNP dynamics and surveillance prior to budding yeast mRNA export. Genes Dev *24*, 1927–1938. 10.1101/gad.583310.
[S25]. Gilbert, W., and Guthrie, C. (2004). The Glc7p nuclear phosphatase promotes mRNA export by facilitating association of Mex67p with mRNA. Mol Cell *13*, 201–212. 10.1016/s1097-2765(04)00030-9.
[S26]. Segref, A., Sharma, K., Doye, V., Hellwig, A., Huber, J., Lührmann, R., and Hurt, E. (1997). Mex67p, a novel factor for nuclear mRNA export, binds to both poly(A)+ RNA and nuclear pores. EMBO J *16*, 3256–3271. 10.1093/emboj/16.11.3256.

[S27]. Strässer, K., Bassler, J., and Hurt, E. (2000). Binding of the Mex67p/Mtr2p heterodimer to FXFG, GLFG, and FG repeat nucleoporins is essential for nuclear mRNA export. J Cell Biol *150*, 695–706. 10.1083/jcb.150.4.695.
[S28]. Tuck, A.C., and Tollervey, D. (2013). A transcriptome-wide atlas of RNP composition reveals diverse classes of mRNAs and lncRNAs. Cell *154*, 996–1009. 10.1016/j.cell.2013.07.047.
[S29]. Baejen, C., Torkler, P., Gressel, S., Essig, K., Söding, J., and Cramer, P. (2014). Transcriptome maps of mRNP biogenesis factors define pre-mRNA recognition. Mol Cell *55*, 745–757. 10.1016/j.molcel.2014.08.005.
[S30]. Ho, B., Baryshnikova, A., and Brown, G.W. (2018). Unification of Protein Abundance Datasets Yields a Quantitative Saccharomyces cerevisiae Proteome. Cell Syst *6*, 192-205.e3. 10.1016/j.cels.2017.12.004.
